# Activation of AMPK ameliorates acute severe pancreatitis by suppressing pancreatic acinar cell necroptosis in obese mice models

**DOI:** 10.1038/s41420-023-01655-z

**Published:** 2023-09-30

**Authors:** Kunlei Wang, Anbang Zhao, Dilinigeer Tayier, Kai Tan, Wenjing Song, Qian Cheng, Xinyin Li, Zhinan Chen, Qifeng Wei, Yufeng Yuan, Zhiyong Yang

**Affiliations:** 1 Department of Hepatobiliary and Pancreatic Surgery, Zhongnan Hospital of Wuhan University, Wuhan, China; 2 Pancreatic Surgery Center, Zhongnan Hospital of Wuhan University, Wuhan, China; 3 Clinical Medicine Research Center for Minimally Invasive Procedure of Hepatobiliary & Pancreatic Diseases of Hubei Province, Wuhan, China

**Keywords:** Necroptosis, Pharmacodynamics, Acute pancreatitis

## Abstract

Obese people with acute pancreatitis (AP) have an increased risk of developing severe acute pancreatitis (SAP), which prolongs the length of hospital stay and increases mortality. Thus, elucidation of the mechanisms through which SAP occurs in obese individuals will provide clues for possible treatment targets. Differences in early events in obese or lean patients with AP have not been conclusively reported. We selected C57BL/6 mice as lean mice models, ob/ob mice or diet induced obese (DIO) mice as obese mice models and then induced experimental AP in mice via injections of caerulein. There were suppressed p-AMPK expressions in the pancreas of obese mice, compared with same-age lean C57BL/6 mice, which were further reduced in AP mice models. Obese AP mice were treated using AICAR, a direct AMPK agonist, which prevented pancreatic damage and cell death, suppressed pancreatic enzyme levels in serum, reduced the areas of fat saponification in the peritoneal cavity, prevented injury in other organs and decreased mice mortality rate. Further assays showed that AICAR activates p-AMPK to stabilize pro-caspase-8. Pro-caspase-8 enhances RIPK3 degradation, inhibits pancreatic acinar cell necroptosis, and downregulates the release of pancreatic enzymes. Thus, activation of AMPK by AICAR alleviates pancreatic acinar cell necroptosis and converts SAP to mild acute pancreatitis in obese mice.

## Introduction

Acute pancreatitis (AP) is a common pancreatic inflammatory disease that is associated with a morbidity rate of 34 cases per 100 000 person-years and 1.60 deaths per 100 000 person-years [1]. From 1961 to 2016, global incidences of AP increased at an average of 3.07% annual percent change [2]. While often mild and self-limiting, about 20% of patients progress to a more severe form with organ failure, pancreatic tissue necrosis, peripancreatic tissue necrosis and up to a 20-40% mortality rate [3]. Since there are no specific internationally licensed drugs to treat AP and prevent the associated complications, symptomatic support therapies and nutritional support therapies have been used for disease management [4]. Obesity is an established risk factor for AP and has been correlated with increasing incidences of inpatient hospitalization due to AP [5], development of SAP from AP with a higher probability of severe systemic inflammatory responses and organ failure [6–8], as well as increased mortality rate [8, 9]. The mortality rate for AP patients with morbid obesity has not exhibited any decrease while the overall AP-associated mortality rate has decreased in recent years [5].

The mechanism via which obesity leads to SAP has been a topic of study for ages. Hypertriglyceridemia is an independent risk factor for local complications and persistent organ failure [10, 11]. Pancreatic lipases can induce the lipolysis of adipocyte triglycerides to non-esterified free fatty acids (NEFA). Unsaturated fatty acids (UFAs) induce acinar cell injury, promotes local pancreatic injury and mediates lipotoxicity as well as progression to multisystem organ failure [12–15]. Obesity results in a low-grade pro-inflammatory state, and more pro-inflammatory cytokines are activated in AP [16–19]. These pathologic mechanisms are potential targets for specific treatments aimed at putative critical pathways, and experimental treatment drugs include the lipase inhibitor (orlistat) [20], the antidiabetic sulfonylurea drug (glyburide) [18], and the antioxidant carotenoid (astaxanthin) with anti-inflammatory properties [21]. These drugs have been shown to protect obese mice from AP. Therefore, understanding the key mechanisms involved in aggravation of AP to a much more severe type in obesity and identification of possible prevention and treatment targets are necessary.

Pancreatic acinar cells (PACs) are involved in exocrine secretion with high levels of protein metabolism and use of energy. They play a significant role in early events during the development of AP [22, 23]. Obesity and leanness differ with regards to energy metabolism levels, while obesity often results in impaired protein metabolism [24]. AMPK is often activated by phosphorylation of Thr172 of the α subunit and regulates synthesis or catabolism [25, 26]. There is a negative association between AMPK activities and obesity/inflammation [27, 28]. Consistently, there are very high energy need in PACs in pancreatitis [22]. There were changes in p-AMPK protein levels in mice and rats with caerulein-induced and L-arginine-induced pancreatitis, and high p-AMPK protein levels were observed in pancreas in mild pancreatitis [29, 30]. These findings suggest that AMPK activity levels may be further impaired in PACs of obese mice in AP and are associated with the severity of obesity-linked AP.

In AP, both apoptosis and necrosis patterns have been shown to occur in PACs [31]. Necroptosis is accompanied by damage to the plasma membrane and since apoptosis maintains a complete plasma membrane, there is more content release and inflammatory activation, when compared with apoptosis [32]. A series of events occur in cells as apoptosis transforms into necrosis, with intracellular ATP levels remaining low (which indicate impaired AMPK activities) [33]. Less acinar cell necrosis was shown in RIPK3 knockout littermates [34, 35] and MLKL^−/−^ littermates [36] in AP, compared with wild-type mice. This indicates that necroptosis is a highly regulated necrosis mode that is mediated by intrinsically defined mechanisms in PACs. It plays important roles in aggravating acinar cell damage in AP.

We used the AMPK agonist (5-Aminoimidazole-4-formamide ribonucleotide (AICAR)), which is phosphorylated in the cytosol by adenosine kinase and converted to AICAribotide (ZMP), mimics AMP and activates AMPK [37], and the AMPK inhibitor (Compound C dihydrochloride (CC)), which is a potent reversible inhibitor that competes with ATP [38]. We found that activation of AMPK increases protection in obese AP by suppressing pancreatic acinar cell necroptosis.

## Results

### Obese mice exhibited higher mortality rates and more severe pancreatic necrosis compared with lean WT mice

**Fig. 1:**
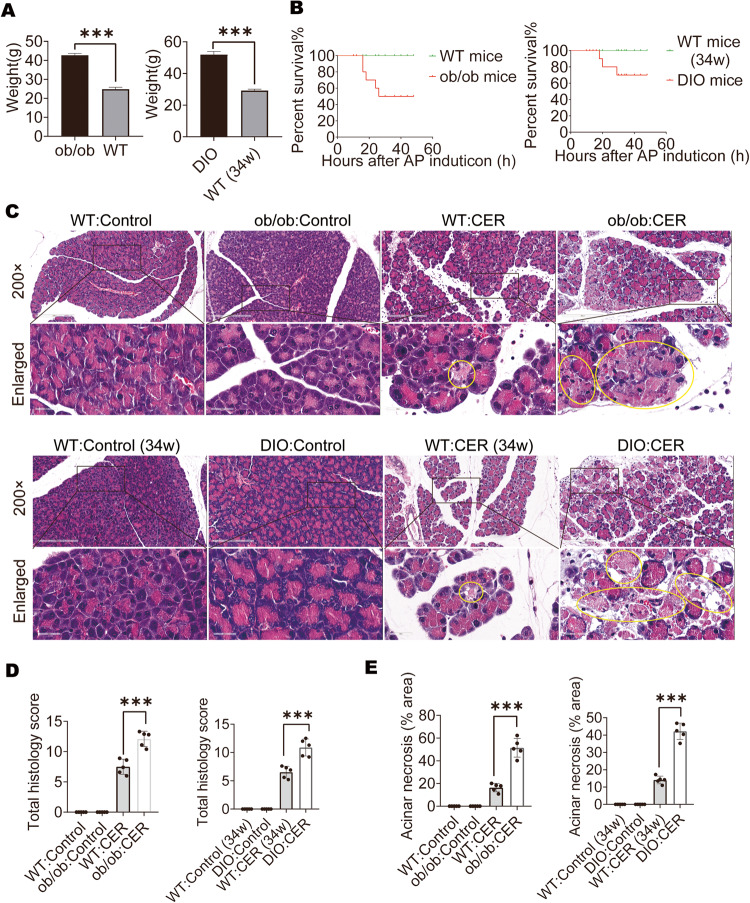
Obese mice exhibited higher mortality rates and more severe pancreatic necrosis compared with lean WT mice. **A** Weight of ob/ob mice, DIO mice, or C57BL/6 mice at the same age (*n* = 12). **B** Mortality of AP mice treated with CER (*n* = 10). **C**–**E** Representative images (×200) and enlarged images (×734) of H&E-stained pancreatic sections, the yellow outlines indicate acinar cell necrosis; the total H&E scores are shown and acinar cell necrosis are expressed as a percentage of total pancreas in AP (*n* = 5). Data are expressed as means ± SEM; *P < 0.05, ***P < 0.001.

The ob/ob mice or DIO mice weighed more than 70% of the C57BL/6 (lean) mice at the same age (Fig. 1A). The AP animal models were established via repeated ten hourly intraperitoneal injections of CER (100 µg/kg). There were 50% and 30% mortality rates in ob/ob mice and DIO mice at 48 h after CER administration, respectively. The lean mice did not exhibit any mortality (Fig. 1B). H&E-stained pancreatic sections from obese mice exhibited intense edema and inflammatory infiltrations as AP developed (Fig. 1C). Histological scores and acinar necrosis in obese mice exhibited severe damage, relative to lean mice of the same age (Fig. 1D, E). These data show that AP in obese mice is more severe than in lean mice.

### Activation of AMPK inhibited pancreatic damage and improved survival outcomes in obese mice

**Fig. 2:**
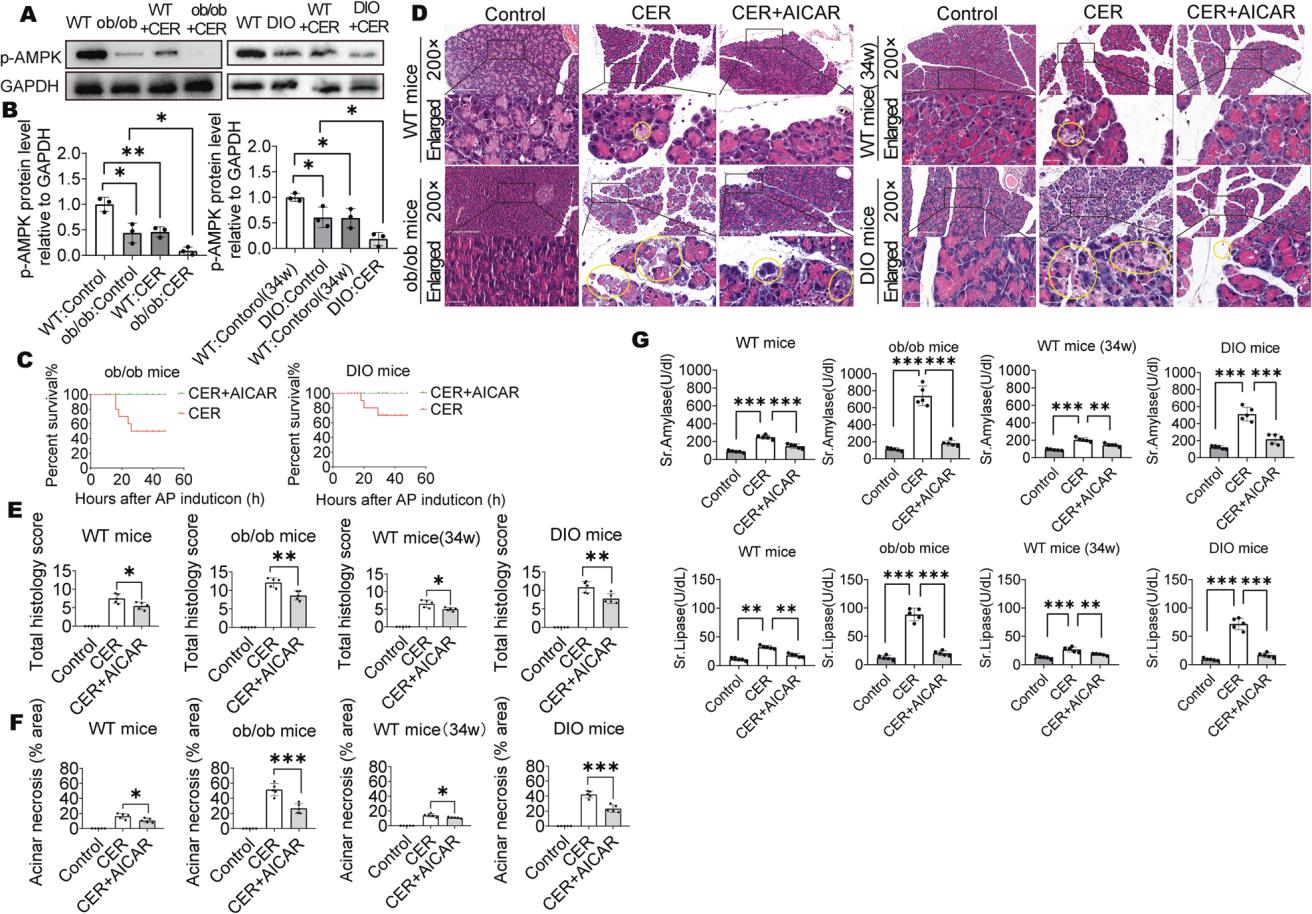
Activation of AMPK inhibited pancreatic damage and improved survival outcomes in obese mice. **A**, **B** WB analysis of p-AMPK in mice pancreas (*n* = 3). **C** Mortality of AP mice treated with or without AICAR (*n* = 10). **D**–**F** Representative images (×200) and enlarged images (×734) of H&E-stained pancreatic sections, the yellow outlines indicate acinar cell necrosis; the total H&E scores and percentage of acinar cell necrosis area are shown (*n* = 5). **G** Serum amylase and lipase (*n* = 5). Data are expressed as the means ± SEM, *P < 0.05, **P < 0.01, ***P < 0.001.

To confirm that AMPK activation levels in obese mice were lower than in lean mice, we collected mice pancreatic tissues and evaluated p-AMPK expressions by Western blot. p-AMPK expressions were suppressed in obese mice, relative to lean WT mice at the same age, and they were further reduced during caerulein-induced AP development (Fig. 2A, B). These results imply a protective possibility for treating obese mice with AP using the AMPK agonist, AICAR. At 48 h, there were 50% and 30% mortality rates in ob/ob mice and DIO mice in caerulein-induced AP. Treatment of both ob/ob and DIO mice with the AMPK agonist (AICAR) markedly reduced the mortality rates to 0% (Fig. 2C). Moreover, AICAR also reduced local pancreatic injury, especially in obese mice. The AICAR-treated obese mice group showed improved histologic scores and acinar necrosis, when compared with the non-AICAR-treated obese mice group (Fig. 2D–F). Serum amylase and lipase levels exhibited decreasing trends after AICAR treatment of obese mice during caerulein-induced AP development (Fig. 2G). These findings suggest that AICAR can decrease acinar cell necrosis as well as histology scores in the pancreas, and finally improve survival outcomes in caerulein-induced AP obese mice.

### Activated AMPK inhibited fat saponification, lung damage, and kidney damage in obese mice

**Fig. 3:**
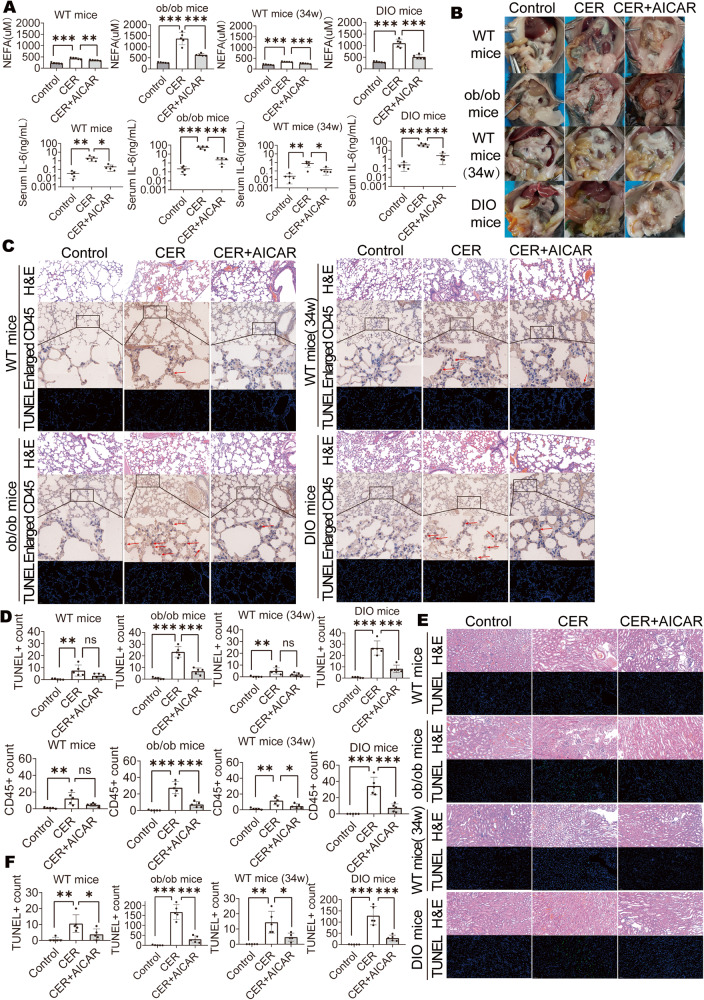
Activated AMPK inhibited fat saponification, lung damage and kidney damage in obese mice. **A** Serum NEFA and IL-6 (*n* = 5). **B** Representative photographs showing the abdominal fat saponification. **C**, **D** Representative H&E staining (×200), CD45 staining (×200 and ×734) and immunofluorescent TUNEL staining (×200) of lung, the red arrows indicate CD45+ cells; Quantification for CD45+ cell and TUNEL+ cell (*n* = 5). **E**, **F** Representative H&E staining (×200) and immunofluorescent TUNEL staining (×200) of kidney; Quantification for TUNEL+ cell (*n* = 5). Data are expressed as means ± SEM, *P < 0.05, **P < 0.01, ***P < 0.001.

Then, we assessed the damage to surrounding organs. Serum NEFA levels and IL-6 levels exhibited decreasing trends after AICAR treatment of obese mice during caerulein-induced AP development (Fig. 3A). Lipolysis was assessed by detecting abdominal fat necrosis in the abdominal cavity [12, 18, 20]. There was obvious abdominal fat necrosis in obese mice in AP, but was decreased in AICAR-treated mice (Fig. 3B). There were obvious lung inflammatory cell infiltrations and cell death in obese mice in AP, but was decreased in AICAR-treated mice (Fig. 3C, D). TUNEL staining showed obvious kidney damage in obese mice in AP, but was decreased in AICAR-treated mice (Fig. 3E, F). These findings imply that injury in organs and tissues other than the pancreas was also decreased after AICAR treatment of obese mice.

### Inhibition of AMPK activation promoted acinar cell death

**Fig. 4:**
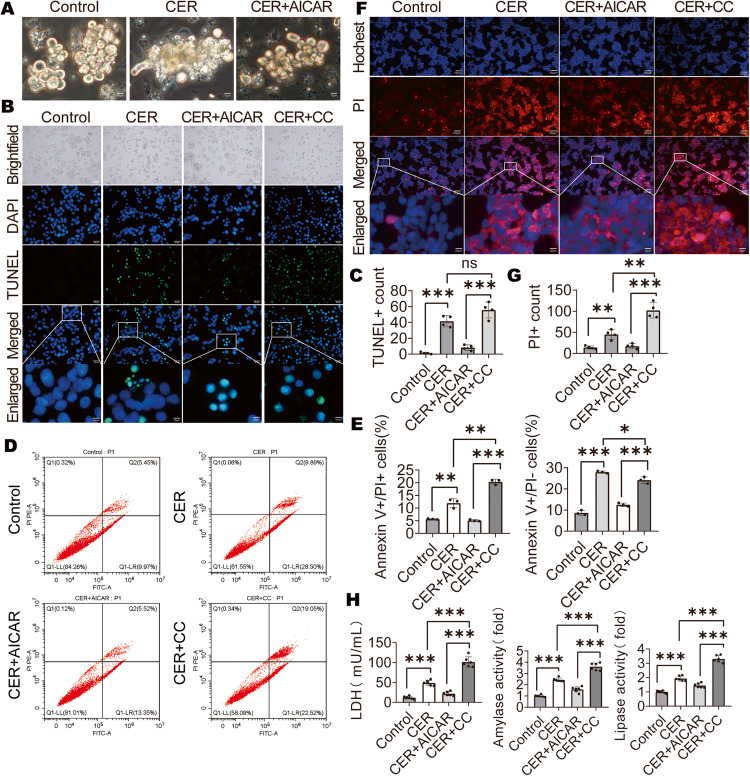
Inhibition of AMPK activation promoted pancreatic acinar cell death. **A** Isolated acinar cells stimulated with CER (1 μM) for 6 h and examined under a microscope. **B**, **C** Representative images and quantification of TUNEL staining (×400) for AR42J cells (*n* = 4). **D**, **E** Flow cytometry and quantification of apoptotic or necrotic cells (n = 3). **F**, **G** Representative images and quantification of Hochest 33342 (blue) and PI (red) staining (×200) for AR42J cells (*n* = 4). **H** LDH, amylase activity and lipase activity in supernatants of AR42J cells (*n* = 6). Data are expressed as means ± SEM, *P < 0.05; **P < 0.01, ***P < 0.001.

Pancreatic acini from obese mice exhibited an injured phenotype after treatment with CER but were improved after treatment with AICAR (Fig. 4A). To verify that AICAR improves obese AP by decreasing acinar necrosis, AR42J were used in in vitro experiments and divided into four groups: i. Control group with no treatment; ii. CER + AICAR group treated with CER and AICAR; iii. CER group treated with CER only and iv. CER + CC group treated with CER and CC. Among the four groups, the CER + CC group exhibited the maximum number of TUNEL-positive cells, while the CER + AICAR group had the lowest, except control group (Fig. 4B, C). The TUNEL assay cannot distinguish between apoptosis and necrosis [39]. Apoptosis manifests by a decrease in cell volume, while necrosis usually manifests by cell swelling [32]. The TUNEL-positive cells in the CER + AICAR group were mostly reduced in volume while those in the CER + CC group were mostly increased (Fig. 4B). Moreover, the CER + AICAR group showed a decrease in late apoptotic and necrotic cells (Annexin V + /PI+ cells) and early apoptotic cells (Annexin V + /PI− cells) while the CER + CC group showed an increase in late apoptotic and necrotic cells (Annexin V + /PI+ cells) (Fig. 4D, E). Hochest 33342 (blue) and PI (red) staining and PI+ count of PACs showed that necrotic cells (Hochest + /PI +) were mostly increased in the CER + CC group, while those in the CER + AICAR group were the least (Fig. 4F, G). Released LDH indicated cell membrane damage in dead cells. Released LDH levels and amylase activities as well as lipase activities in the CER + CC group were more than those of the CER + AICAR group (Fig. 4H). These findings suggest that low activated AMPK levels are accompanied by high rates of cell necrosis, indicating the possibility that p-AMPK can regulate cell death.

### Activation of AMPK inhibited necroptosis by regulating Caspase8

**Fig. 5:**
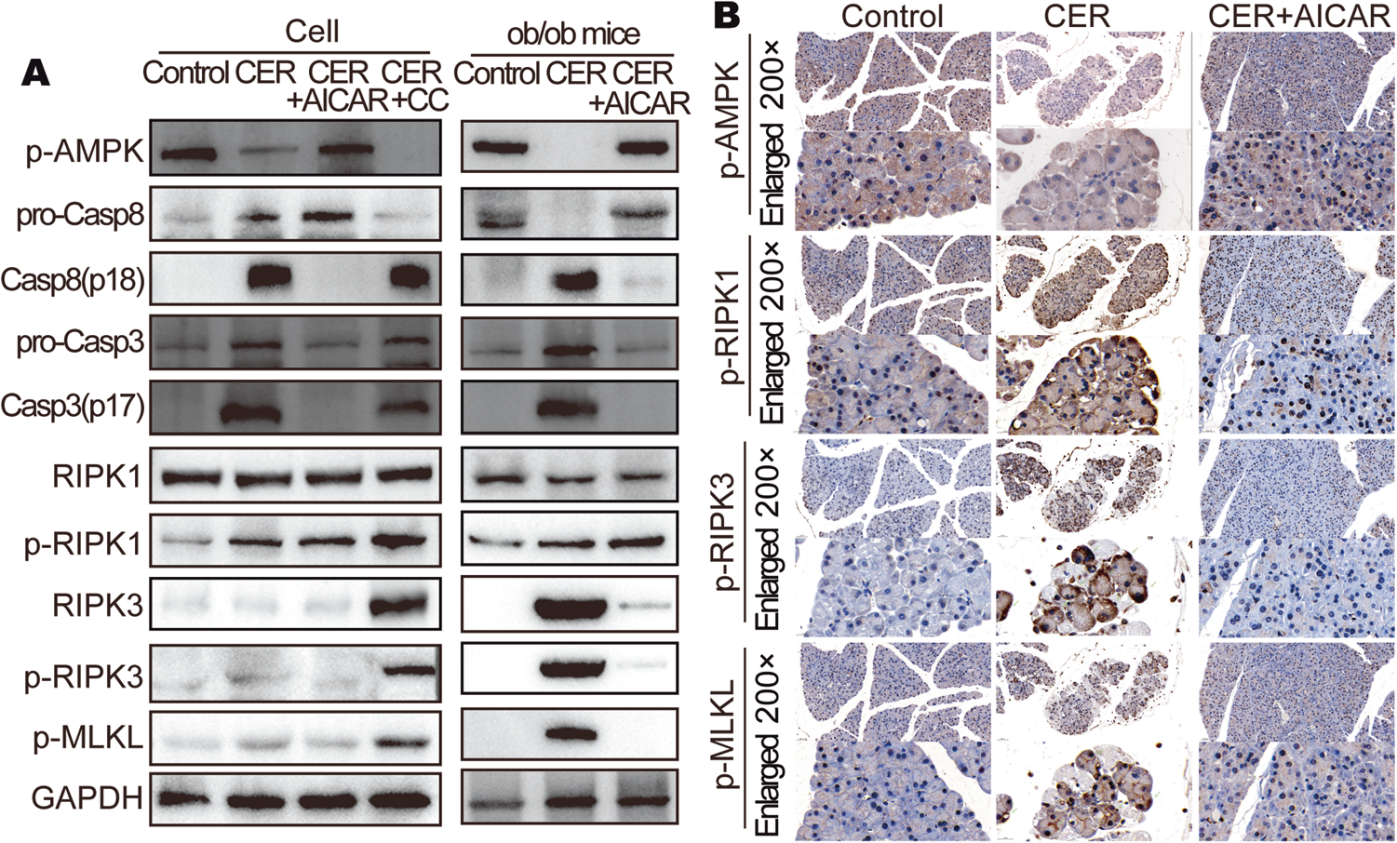
Activation of AMPK inhibited necroptosis in pancreatic acinar cell. **A** WB analysis of p-AMPK, Casp8, Casp3, RIPK1, RIPK3, p-RIPK1, p-RIPK3, and p-MLKL of ob/ob mice pancreas and AR42J cells. **B** Representative images (200×) and enlarged images (734×) of serial sections stained with p-AMPK, p-RIPK1, p-RIPK3, and p-MLKL of ob/ob mice pancreas.

Western blot showed higher expression level of pro-caspase-8 (pro-Casp8) in CER + AICAR group obese mice in AP and CER + AICAR group cell, while the opposite trend was observed in the CER group obese mice and CER + CC group cell (Fig. 5A and Fig. S2A). Casp8 can cleave RIPK1 [40] or RIPK3 [41]. We noted equal RIPK1 levels and suppressed RIPK3 levels while pro-Casp8 levels were elevated, suggesting that pro-Casp8 can cleave RIPK3 in PACs. p-RIPK1 and p-RIPK3 levels were comparable to those of RIPK1 and RIPK3, excluding the possibility that p-AMPK or CASP8 are involved in phosphorylation of RIPK1 or RIPK3 (Fig. S2B). p-MLKL, which causes necroptosis, is activated when both RIPK1 and RIPK3 levels are elevated [32]. While RIPK3 was suppressed in CER + AICAR group, necroptosis was inhibited (Fig. 5A). These outcomes imply that higher levels of p-AMPK and pro-Casp8 inactivated the necroptosis pathway-associated proteins (RIPK3 and p-MLKL) in PACs during caerulein-induced AP development. p-MLKL was observed to aggregate into the cell membrane in damaged PACs in obese AP (Fig. 5B).

**Fig. 6:**
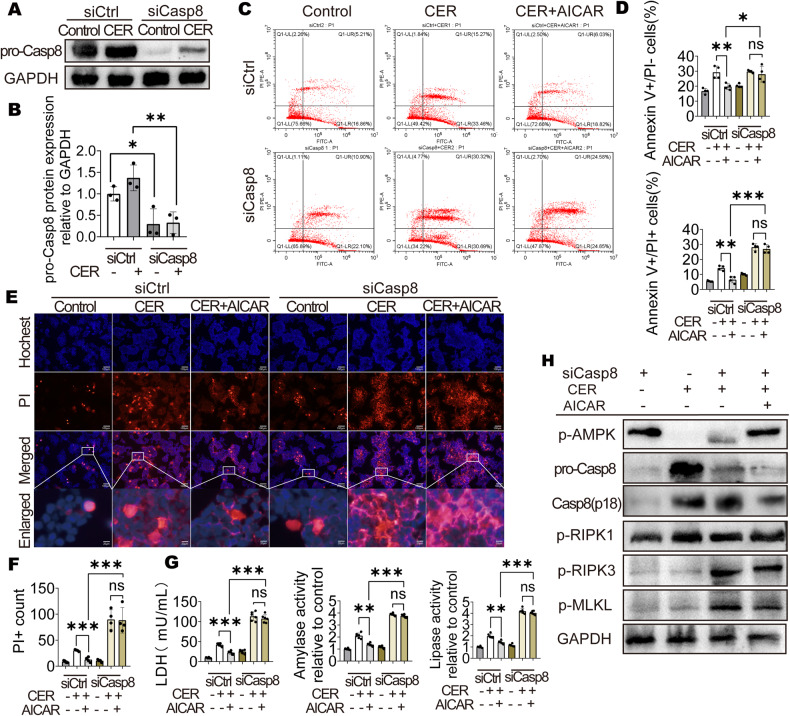
Activation of AMPK inhibited necroptosis by regulating Casp8. **A**, **B** WB analysis of pro-Casp8 in AR42J cells (*n* = 3). **C**, **D** Flow cytometry result and number of apoptotic or necrotic cells (*n* = 3). **E**, **F** Representative images and quantification of Hochest 33342 (blue) and PI (red) staining (200×) for AR42J cells (*n* = 4). **G** LDH, amylase activity and lipase activity in supernatant of AR42J cells (*n* = 6). **H** WB analysis of p-AMPK, Casp8, p-RIPK1, p-RIPK3, and p-MLKL in AR42J cells. Data are expressed as means ± SEM; *P < 0.05; **P < 0.01, ***P < 0.001.

To confirm that there were interactions between p-AMPK and Casp8, Casp8 knockdown was performed using siRNA (Fig. 6A, B). AICAR was ineffective in improving cell death after Casp8 knockdown (Fig. 6C–F). After Casp8 knockdown, released LDH levels as well as amylase activities and lipase activities were not suppressed in the CER + AICAR group (Fig. 6G). Moreover, although AMPK had been activated, RIPK3 and p-MLKL levels were elevated after Casp8 knockdown (Fig. 6H and Fig. S3A). These results confirm that Casp8 plays a key role in AMPK-mediated improvement of necroptosis.

### p-AMPK enhanced pro-Casp8 stability

**Fig. 7:**
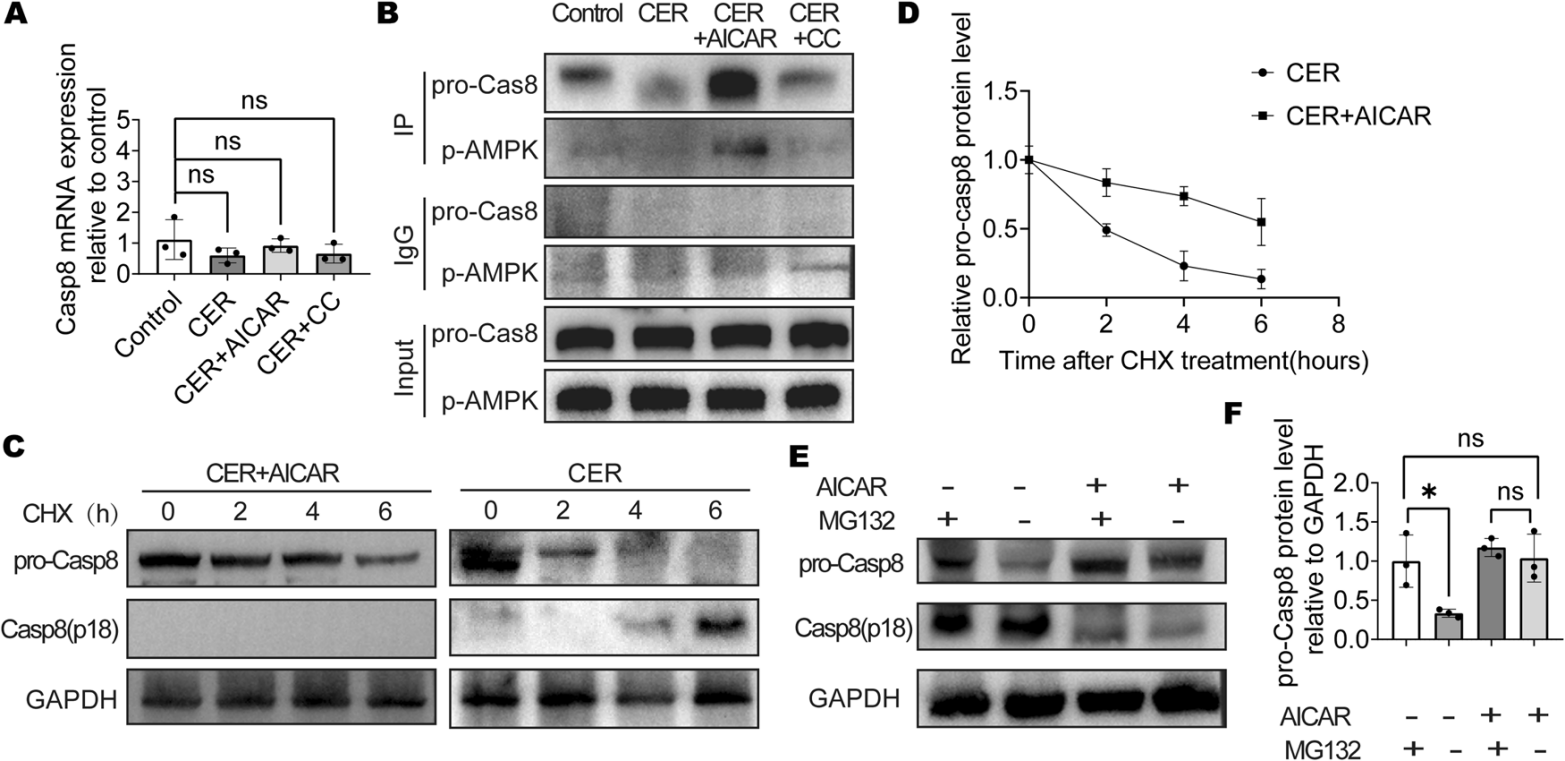
p-AMPK enhanced pro-Casp8 stability. **A** qRT-PCR analysis of Casp8 expression in AR42J cells. **B** Co-IP assay results in AR42J cells. **C, D** AR42J was treated with cycloheximide (CHX, 10 μg/ml) 1 h before addition of CER. WB analysis of Casp8 of AR42J cells. **E**, **F** AR42J was treated with 10nmol/L MG132 8 h before the addition of CER, WB analysis of pro-Casp8 of AR42J cells.

**Fig. 8:**
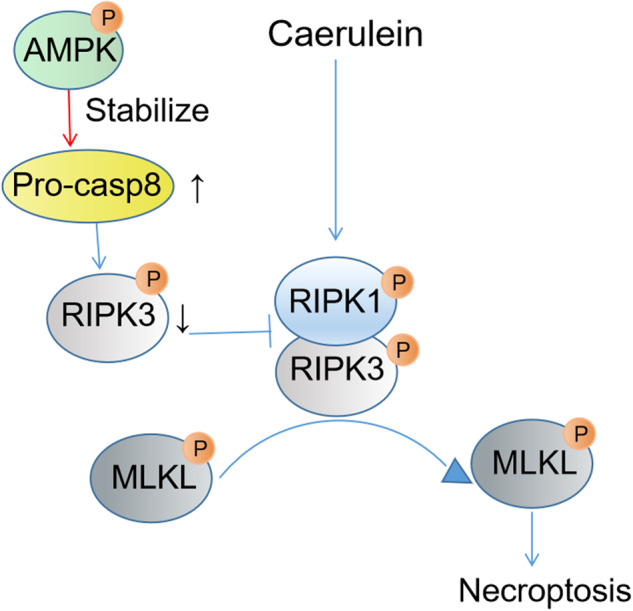
The proposed mechanism by which AMPK activation reduces acinar cells necroptosis.

Differences in Casp8 mRNA expressions among the groups did not exhibit marked differences (Fig. 7A). The Co-IP assay revealed that Casp8 and p-AMPK can directly bind, especially in the AMPK-activated group (Fig. 7B). Since p-AMPK enhanced pro-Casp8 expression levels but not work in protein synthesis, we aimed at establishing whether p-AMPK is involved in the degradation of pro-Casp8. Analysis of pro-Casp8 protein stability using cycloheximide (CHX) confirmed that p-AMPK enhanced po-Casp8 stability (Fig. 7C, D). The proteasome inhibitor (MG132) reversed the decrease in pro-Casp8 levels, suggesting that p-AMPK upregulates pro-Casp8 levels by inhibiting its proteasomal degradation (Fig. 7E, F). Thus, activated AMPK reduces acinar cell necroptosis by regulating pro-Casp8 stability (Fig. 8). In conclusion, p-AMPK enhances pro-Casp8 stability and inhibits necroptosis.

## Discussion

In this study, we aimed at elucidating on the mechanisms involved in the development of SAP and to identify potential prevention and treatment targets. The p-AMPK protein levels were significantly differentially expressed between the lean and obese mice, and impaired p-AMPK levels were associated with severe pancreatic necrosis in cerulean-induced AP. AICAR-activated AMPK markedly suppressed the levels of pro-Casp8 in obese mice pancreas, suppressed necroptosis-pathway activation in AP and reduced acinar cell necrosis. Then, it reduced the release of cell contents, including pancreatic enzymes (serum lipase and amylase) and non-esterified free fatty acids, decreased the areas of abdominal fat necrosis, inhibited lung injury, decreased the renal tubular vacuoles, and finally, significantly improved the survival rate of obese mice.

There was a higher mortality rate in obese mice in caerulein-induced AP, relative to lean mice under the same dose of CER. By measuring pancreatic p-AMPK protein levels, we found that p-AMPK levels in obese mice were significantly lower than those in lean mice, which were further inhibited in caerulein-induced AP. Pro-Casp8 levels were higher and acinar cell necroptosis was less in mice with high p-AMPK levels in caerulein-induced AP. Casp8 is a key protein in the regulated cell death pathway. The key role of pro-Casp8 is inhibiting necroptosis, while the key role of activated Casp8 (cleaved Casp8) is activating the apoptotic pathway, therefore, Casp8 is the key hub in apoptosis and necroptosis [42]. Pro-Casp8 can cleave the corresponding sites on RIPK1 and RIPK3 [43], thereby blocking the necroptotic pathway, while on the other hand, pro-Casp8 can activate subsequent apoptotic pathways by cleaving itself, thereby inducing apoptosis [44, 45]. Thus, suppression of pro-Casp8 leads to weakening of its inhibitory effects on necroptosis, so that cells are more prone to the necroptosis pathway when they are stimulated.

The late events in SAP individuals are complex. Amplified inflammatory response, increased UFA and lipotoxicity and hypertriglyceridemia all contribute to peripheral organ injury and are difficult to distinguish which is the original event in organ damage, for they also can strengthen each other and two or more events may happen in one organ injury [10–19, 46, 47]. We observed increased fat necrosis, lung injury, kidney damage in obese mice. And most important, we found there was increased acinar necrosis in obese mice. These results indicated the feasibility of decreasing acinar cell necroptosis to prevent other organs injury by activating AMPK. The study of Patel et al. [20] showed that lipolysis effects of pancrelipase on fat, leading to lipotoxicity of elevated free fatty acids, is very obvious and crucial to disease severity. In comparison, there were no marked differences in the proportions of pancreatic necrotic acinar cells with regards to severe and mild pancreatitis (the difference in necrotic area <10%). According to Oliveira’s research, they found it is pancreatic triglyceride lipase mediates lipotoxic instead of adipocyte triglyceride lipase in obese AP [48]. There were more studies indicated the increased pancreatic necrosis area is highly associated with multiple organ injury and progression to a much more severe type in AP patients [49–51]. Moreover, compared with lean patients (BMI < 30), the areas of necrotic acinar cell and NEFA levels were found to be increased in pancreatic tissue sections of obese AP patients (BMI > 30) [12]. This confirms that pancreatic necrosis and released pancreatic triglyceride lipase are essential for lipotoxicity and organ injury in obese AP and decrease pancreatic cell death is meaningful in improving obese AP outcomes.

Activation and leakage of digestive enzymes in PACs are the key pathogenic mechanisms and early events [22]. Both apoptosis and necrosis occur in PACs, and it is clear that differences between different cell death modes (necrosis and apoptosis) will influence the subsequent release of cell contents and induction of inflammation, which is determined by the characteristics of apoptosis and necrosis. Apoptosis maintains membrane integrity and is associated with decreased cellular volume (pyknosis), while necrosis is characterized by increased cell volume (oncosis) and plasma membrane disruption [52]. We found that the form of necrosis in acinar cells is mainly necroptosis, which is a death form that relies on MLKL-initiated membrane perforations. Membrane pore sizes have been estimated to be 4 nm in size, thereby allowing ion-selective damage. This channel impacts intracellular osmolarity, causes cell swelling, subsequent osmolysis and disrupts cells [53, 54]. The necrosis-related properties of necroptosis make it easier for more cell contents to be released to the surroundings, which provides a theoretical possibility for more pancreatic enzyme leakage and heavier pancreatic cell injury. We verified it by checking the area of pancreatic pathological damage as well as the degree of inflammatory cell infiltrations and serum pancreatic enzyme as well as NEFA levels.

The severity of AP may be directly related to the degree of necrosis in pancreatic cells [31]. Studies have explored strategies for decreasing the proportion of necrotic acinar cell in pancreatitis as an avenue for treating AP. In knockout mice models, including RIPK3^-/-^ [34] and MLKL^-/-^ [36], which had milder AP compared with wild-type mice under the same dose of CER, it was found that necroptosis played a role in pancreatic cells damage and development of AP. Other research reported that the RIPK1 inhibitor Nec-1 had no effect on the pathological damage associated with AP in mice. In contrast, the use of the apoptosis inhibitor TAT-crmA or the simultaneous use of Nec-1 and pan-caspase inhibitor zVAD aggravated the pathological damage of the pancreas, and increased the content of serum amylase and lipase. The author suggests that the effects of pharmacologically blocking and genetic deficiency in the key proteins in the regulated cell death network are different [55]. According to the recommendations of the Cell Death Nomenclature Committee in 2018 [32], interventions to counteract crisis or the causes in cell death rather than epiphenomena may achieve true cytoprotection. Apoptosis transforms into necrosis in cells while intracellular ATP levels remain low (which indicates impaired AMPK activities) [56, 57]. Activation of AMPK can stabilize Casp8. Casp8 is an upstream and key protein in the regulation and initiation of programmed cell death [42]. Both apoptosis and necroptosis are important pathways involved in the regulation of cell death networks [58]. In our study, activation of AMPK by AICAR increased pro-Casp8 expression to regulate cell death by targeting apoptosis and necroptosis. This has also been proven to improve outcomes in SAP in mice. Wittkopf et al. [59] reported that extrinsic signals activated Casp8 to regulate the apoptosis or necrosis of intestinal epithelial cells. The regulatory mechanism of Casp8 is very complex, Phosphorylation of different sites or promoting pro-Casp8 expression all can regulate the apoptotic pathway or necroptotic pathway [41, 42, 44, 60]. We further found that activated AMPK decreased cell death by regulating Casp8 and AMPK bound to po-Casp8 to increase the stability of pro-Casp8. Together, these outcomes provide ideas for the treatment of regulated cell death in the future.

In summary, our findings lead to the following conclusions: (1) There are lower pancreatic p-AMPK protein levels, higher pancreatic acinar cell death and higher mortality rate in obese AP mice while compared with lean mice. (2) Activation of AMPK increase pro-Casp8 protein levels to enhance RIPK3 degradation and inhibit the necroptotic pathway in PACs in caerulein-induced AP. (3) Activation of AMPK improves caerulein induced AP by decreasing pancreatic acinar cell death in the early stage of AP in obese mice. It also decreases inflammatory response and serum lipase, hydrolysis of unsaturated fats, lung injury, kidney damage and mice mortality rate. These findings suggest that p-AMPK plays a protective role in caerulein-induced obese AP. Considering the difficulty of obtaining clinical AP samples, we did not verify our findings in clinical samples.

## Materials and methods

### Animal studies

We used 6–8 weeks old male ob/ob (obese) mice or 4–8 weeks old male C57BL/6 (lean) mice that had been obtained from Beijing HFK Bio-Technology Co. LTD. Mice were allowed to acclimatize to the facility environment for 1 week before inducing AP with caerulein (CER). All mice were raised in the SPF animal facility of the Center for Animal Experiment, Wuhan University under sterile conditions in air-filtered containers. Rodent diet with 60% kcal fat was purchased from Jiangsu Xietong pharmaceutical and Biological Engineering Co. Ltd and fed to the diet-induced obese mice (DIO mice), while other mice were fed on standard laboratory chow. DIO mice were fed on rodent diet with 60% kcal fat for 30 weeks from week 4, and were 70%-90% heavier than C57BL/6 mice that had been fed on standard laboratory chow at the same age. All animal experiments were approved by IACUC of the Wuhan University Center for Animal Experiment (Wuhan, China), and all efforts were made to minimize animal suffering.

Induction of AP in C57BL/6 mice, ob/ob mice and DIO mice was achieved via ten hourly intraperitoneal administrations of 100 μg/kg CER (17650-98-5, Yuanye Biotechnology, China) after mice were fasted overnight. Mice were injected with 100 mg/kg AICAR (HY-13417, MedChemExpress, China) or 20 mg/kg CC (HY-13418, MedChemExpress, China) or the same dose of normal saline 1 h before the start of CER injection. Mice were observed after CER administration. At 24 h or 48 h after first injections of CER, mice were anesthetized and euthanized by cervical dislocation, after which their tissues were collected for analyses. Blood samples were collected at 24 h after the first injection of CER. All mice were grouped randomly.

### Cell culture

AR42J (CL-0025, Procell, China) were kindly provided by Procell Life Sciences & Technology Co. Ltd and cultured in Ham’s F-12 K medium (Procell) supplemented with 20% fetal bovine serum (10099-141, Gibco, USA). AR42J cells were confirmed for Mycoplasma negativity. AR42J cells were plated into 6 well plates, incubated for 24 h and treated with 1μmol/L CER. At 1 h prior to CER treatment, the culture medium was supplemented with 1 mmol/L AICAR (AMPK agonist group) or 10μmol/L CC (AMPK inhibitor group). Then, culture supernatants and cells were obtained at 6 h after CER treatment for further analyses.

At 1 h before CER treatment, the F12K complete medium was supplemented with 10 μg/ml Actinomycin D (CHX, A9415, Sigma-Aldrich, USA) to assess protein stability in AR42J cells. Proteins were extracted at 0, 2, 4, and 6 h after CER treatment for Western Blot analysis. At 2 h before CER treatment, the F12K complete medium was supplemented with 10 nM MG132 (HY-13259, MedChemExpress, China).

### Acinar cell preparation

Acinar cells were isolated from 6- to 8-week-old ob/ob mice and prepared as previously described [61]. Mice were killed by cervical dislocation after anesthesia, and their pancreas collected. The pancreas was sliced into small pieces and digested using Collagenase IV solution (HBSS 1×containing 10 mM HEPES, 200 U/ml collagenase IV, and 0.25 mg/ml trypsin inhibitor) under shaking (60 rpm/min, 40 min, 37°C). The enzymatic reactions were stopped by the addition of 20% FBS F12K complete medium after which the solution was filtered through a 200-µm filter. The sample was centrifuged twice for 5 min at 500×g to obtain isolated acini. The extracted samples were cultured in complete medium at 37 °C in a 5%CO_2_ atmosphere.

### Western Blot

Protein lysates were obtained from AR42J cells or mice pancreas and lysed in a RIPA buffer that had been supplemented with proteinase and phosphatase inhibitors. The BCA protein assay kit (PC0020, Solarbio, China) was used to measure protein lysates. About 20 μg protein lysates were loaded on a 10% polyacrylamide gel. Antibodies against cleaved Caspase-8 (9429, Cell Signaling Technology), caspase-8 (4790, Cell Signaling Technology), caspase-8 (13423-1-AP, Proteintech, USA), caspase-3 (9662, Cell Signaling Technology), p-AMPKα (2535, Cell Signaling Technology), RIPK1 (3493, Cell Signaling Technology), RIPK3 (15828, Cell Signaling Technology), p-RIPK1 (38662, Cell Signaling Technology), p-RIPK3 (ab222320, Abcam), p-MLKL (37333, Cell Signaling Technology), GAPDH (10494-1-AP, Proteintech, USA) were used, Goat Anti-Rabbit IgG(H + L, SA00001-2, Proteintech, USA) was used as the secondary antibody. Images of immune complexes were obtained by the enhanced chemiluminescence (ECL) detection system (TANON SCIENCE & TECHONLOGY CO, Shanghai, China). The assays were performed in triplicate.

### H&E staining, immunohistochemistry and immunofluorescence

All tissues were collected and fixed in 4% paraformaldehyde overnight. Fixed tissues were paraffin-embedded, sliced into 5μm thick sections and stained with haematoxylin and eosin (H&E). Pancreatic histopathological scores were evaluated by inflammatory cell infiltrations, hemorrhage and necrosis, from 0 to 4 [62]. Ten randomly selected high-power fields (200×) were assessed per slide. Pancreatic histopathological scores were carefully scored by two researchers who were blinded to the group allocation during the experiment independently according to scoring criteria. Immunohistochemistry was performed using the avidin-biotin-peroxidase complex method. TUNEL staining was performed using the TUNEL kit (G1504, Servicebio, China), as instructed by the manufacturer.

### Evaluation of necrotic and apoptotic cell death

The AR42J cells were fixed in 4% buffered formaldehyde for 30 min and their apoptosis assessed using the TUNEL kit (11684817910, Roche), as instructed by the manufacturer. The Hoechst 33342/PI Kit (BB-4131, BestBio, China) was used to evaluate AR42J cell necrosis. The acinar cells were treated with a staining buffer, stained with Hoechst 33342 for the nuclear stain and PI for assessing plasma membrane rupture as a marker of necrotic cell death pathway activation. Images were acquired by fluorescence microscopy (Olympus, Tokyo, Japan).

### Biochemical measurements

Serum or supernatant amylase, lipase, non-esterified free fatty acid (NEFA) as well as lactate dehydrogenase (LDH) levels were measured using their respective kits (Nanjing Jiancheng Bioengineering Institute, China). Serum IL-6 levels were measured using their respective kits (EK206, Multi Science, China). Amylase activities of acinar cells were measured using kits from Sigma-Aldrich (MAK009).

### Flow cytometry

AR42J cells were digested using trypsin, harvested, and washed twice using 1× PBS. Cell apoptosis was evaluated using the Annexin V-FITC/PI apoptosis kit (70-AP101-100, Multisciences, China), as instructed by the manufacturer. Analyses were performed using a cytoflex flow cytometer (Beckman, China).

### RNA isolation and qRT-PCR analysis

EASYspinPlus (RN2802, Aidlab Biotechnologies Co. Ltd, China) was used for isolation of total RNA from AR42J cells, according to the manufacturers’ instructions. cDNA was synthesized using A HiScript® II Q RT SuperMix for qPCR (+gDNA wiper) (R223-01, Vazyme Biotech Co. Ltd, China). qRT-PCR was performed using the ChamQ Universal SYBR qPCR Master Mix (Q711, Vazyme Biotech Co. Ltd, China). The primers were listed as follows: 18s, 5′-CAGCCACCCGAGATTGAGCA-3′ and 3′-TAGTAGCGACGGGCGGTGTG-5′, caspase-8 5′-TCTACGGAACGGATGGGAAGGAAG-3′ and 3′-CACATCATAGTTCACGCCAGTCAGG-5′. Each 96-well system contained 500 ng cDNA and specific reverse as well as forward primers in 20 μL qRT-PCR reaction volume. The internal control gene was 18 s. Relative mRNA expressions were analyzed by the 2^−ΔΔCt^ method.

### siRNA

siRNAs were obtained from Tsingke Biotechnology Co., Ltd. (Beijing, China) and used to knockdown caspase-8 RNA in AR42J cells. Sicaspase-8: 5′-GAGGATTCATCATCTTACA-3′(Target sequance), 5′-GAGGAUUCAUCAUCUUACATT-3′(Sense), 5′-UGUAAGAUGAUGAAUCCUCTT-3′(Antisense). Cells were transfected with 200pmol siRNAs using the GenMuteTM siRNA Transfection Reagent (SL100568, signagen, USA), as instructed by the manufacturer.

### Co-IP

The AR42J cells were harvested from 4 petri dishes for each Co-IP reaction group and washed twice with 1×PBS. After centrifugation, sediments were resuspended in IP lysis buffer that had been supplemented with a protease inhibitor cocktail (MB2678-1, Meilunbio, China) for 30 min. After centrifugation, the supernatant of cell lysate was collected and used 10% of protein supernatant as input. Magnetic beads protein A/G (HY-K0202, MedChemExpress, China) were used to isolated non-specific binding proteins in the rest supernatant. Normal rabbit IgG (sa00001-2, Proteintech, USA) was used in the negative control IP reaction. The remaining protein supernatant was incubated in a beads-antibody complex in IP buffer at 4 °C overnight. The IP buffer was washed thrice, the beads were collected, resuspended in a loading buffer (5×) and heated at 95 °C for 10 min. The mixture was then centrifuged at 12,000*g* at 4 °C for 1 min. Western blot analyses were conducted as described above.

### Statistical analysis

Data are expressed as means ± SEM and were analyzed using Graphpad prism 8 (GraphPad Software Inc., La Jolla, CA, USA). Between group comparisons of means were performed using the Student’s *t* test. Significant levels were set as: ns: *p* > 0.0.5; *p < 0.05; **p < 0.01; ***p < 0.001.

### Supplementary information

Original western blots

Supplemental Materials and legends

## Data Availability

All data supporting the findings of this study are available within the manuscript and its supplementary information files.

## References

[1] Xiao, Tan MLY AY, Wu LM, Asrani VM, Windsor JA, Yadav D (2016). Global incidence and mortality of pancreatic diseases: a systematic review, meta-analysis, and meta-regression of population-based cohort studies. Lancet Gastroenterol Hepatol.

[2] Iannuzzi JP, King JA, Leong JH, Quan J, Windsor JW, Tanyingoh D (2022). Global incidence of acute pancreatitis is increasing over time: a systematic review and meta-analysis. Gastroenterology.

[3] Boxhoorn L, Voermans RP, Bouwense SA, Bruno MJ, Verdonk RC, Boermeester MA (2020). Acute pancreatitis. Lancet.

[4] Szatmary P, Grammatikopoulos T, Cai W, Huang W, Mukherjee R, Halloran C (2022). Acute pancreatitis: diagnosis and treatment. Drugs.

[5] Krishna SG, Hinton A, Oza V, Hart PA, Swei E, El-Dika S (2015). Morbid obesity is associated with adverse clinical outcomes in acute pancreatitis: a propensity-matched study. Am J Gastroenterol.

[6] Premkumar R, Phillips AR, Petrov MS, Windsor JA (2015). The clinical relevance of obesity in acute pancreatitis: targeted systematic reviews. Pancreatology.

[7] Yoon SB, Choi MH, Lee IS, Lim CH, Kim JS, Cho YK (2017). Impact of body fat and muscle distribution on severity of acute pancreatitis. Pancreatology.

[8] Dobszai D, Matrai P, Gyongyi Z, Csupor D, Bajor J, Eross B (2019). Body-mass index correlates with severity and mortality in acute pancreatitis: A meta-analysis. World J Gastroenterol.

[9] Blaszczak AM, Krishna SG, Hart PA, Bradley D, Hsueh W, Lara LF (2020). Class III obesity rather than metabolic syndrome impacts clinical outcomes of acute pancreatitis: a propensity score weighted analysis. Pancreatology.

[10] Yang X, He J, Ma S, Wang T, Zhu Q, Cao F (2021). The role of comorbid hypertriglyceridemia and abdominal obesity in the severity of acute pancreatitis: a retrospective study. Lipids Health Dis.

[11] Nawaz H, Koutroumpakis E, Easler J, Slivka A, Whitcomb DC, Singh VP (2015). Elevated serum triglycerides are independently associated with persistent organ failure in acute pancreatitis. Am J Gastroenterol.

[12] Navina S, Acharya C, DeLany JP, Orlichenko LS, Baty CJ, Shiva SS (2011). Lipotoxicity causes multisystem organ failure and exacerbates acute pancreatitis in obesity. Sci Transl Med.

[13] Chang YT, Chang MC, Tung CC, Wei SC, Wong JM (2015). Distinctive roles of unsaturated and saturated fatty acids in hyperlipidemic pancreatitis. World J Gastroenterol.

[14] Khatua B, El-Kurdi B, Patel K, Rood C, Noel P, Crowell M (2021). Adipose saturation reduces lipotoxic systemic inflammation and explains the obesity paradox. Sci Adv.

[15] Noel P, Patel K, Durgampudi C, Trivedi RN, de Oliveira C, Crowell MD (2016). Peripancreatic fat necrosis worsens acute pancreatitis independent of pancreatic necrosis via unsaturated fatty acids increased in human pancreatic necrosis collections. Gut.

[16] Lee YH, Pratley RE (2005). The evolving role of inflammation in obesity and the metabolic syndrome. Curr Diab Rep.

[17] Yu Q, Xu T, Ding F, Ding Z, Lin R. Decreased infiltration of adipose tissue macrophages and amplified inflammation of adipose tissue in obese mice with severe acute pancreatitis. Pancreatology. 2021;19:S1424–3903. 10.1016/j.pan.2021.05.005 34088592

[18] York JM, Castellanos KJ, Cabay RJ, Fantuzzi G (2014). Inhibition of the nucleotide-binding domain, leucine-rich containing family, pyrin-domain containing 3 inflammasome reduces the severity of experimentally induced acute pancreatitis in obese mice. Transl Res.

[19] Perez S, Rius-Perez S, Finamor I, Marti-Andres P, Prieto I, Garcia R (2019). Obesity causes PGC-1alpha deficiency in the pancreas leading to marked IL-6 upregulation via NF-kappaB in acute pancreatitis. J Pathol.

[20] Patel K, Trivedi RN, Durgampudi C, Noel P, Cline RA, DeLany JP (2015). Lipolysis of visceral adipocyte triglyceride by pancreatic lipases converts mild acute pancreatitis to severe pancreatitis independent of necrosis and inflammation. Am J Pathol.

[21] Kwak MS, Lim JW, Kim H (2021). Astaxanthin inhibits Interleukin-6 expression in cerulein/resistin-stimulated pancreatic acinar cells. Mediators Inflamm.

[22] Saluja A, Dudeja V, Dawra R, Sah RP (2019). Early intra-acinar events in pathogenesis of pancreatitis. Gastroenterology.

[23] Yule DI (2010). Pancreatic acinar cells: molecular insight from studies of signal-transduction using transgenic animals. Int J Biochem Cell Biol.

[24] Guillet C, Masgrau A, Walrand S, Boirie Y (2012). Impaired protein metabolism: interlinks between obesity, insulin resistance and inflammation. Obes Rev.

[25] Garcia D, Shaw RJ (2017). AMPK: mechanisms of cellular energy sensing and restoration of metabolic balance. Mol Cell.

[26] Hasanvand A, Amini-Khoei H, Hadian MR, Abdollahi A, Tavangar SM, Dehpour AR (2016). Anti-inflammatory effect of AMPK signaling pathway in rat model of diabetic neuropathy. Inflammopharmacology.

[27] Yang Z, Kahn BB, Shi H, Xue BZ (2010). Macrophage alpha1 AMP-activated protein kinase (alpha1AMPK) antagonizes fatty acid-induced inflammation through SIRT1. J Biol Chem.

[28] O’Neill LA, Hardie DG (2013). Metabolism of inflammation limited by AMPK and pseudo-starvation. Nature.

[29] Piplani H, Marek-Iannucci S, Sin J, Hou J, Takahashi T, Sharma A (2019). Simvastatin induces autophagic flux to restore cerulein-impaired phagosome-lysosome fusion in acute pancreatitis. Biochim Biophys Acta Mol Basis Dis.

[30] Wang XD, Yu WL, Sun Y (2021). Activation of AMPK restored impaired autophagy and inhibited inflammation reaction by up-regulating SIRT1 in acute pancreatitis. Life Sci.

[31] Mareninova OA, Sung KF, Hong P, Lugea A, Pandol SJ, Gukovsky I (2006). Cell death in pancreatitis: caspases protect from necrotizing pancreatitis. J Biol Chem.

[32] Galluzzi L, Vitale I, Aaronson SA, Abrams JM, Adam D, Agostinis P (2018). Molecular mechanisms of cell death: recommendations of the Nomenclature Committee on Cell Death 2018. Cell Death Differ.

[33] Lemasters JJ (2005). Dying a thousand deaths: redundant pathways from different organelles to apoptosis and necrosis. Gastroenterology.

[34] He S, Wang L, Miao L, Wang T, Du F, Zhao L (2009). Receptor interacting protein kinase-3 determines cellular necrotic response to TNF-alpha. Cell.

[35] Louhimo J, Steer ML, Perides G (2016). Necroptosis is an important severity determinant and potential therapeutic target in experimental severe pancreatitis. Cell Mol Gastroenterol Hepatol.

[36] Wu J, Huang Z, Ren J, Zhang Z, He P, Li Y (2013). Mlkl knockout mice demonstrate the indispensable role of Mlkl in necroptosis. Cell Res.

[37] Corton JM, Gillespie JG, Hawley SA, Hardie DG (1995). 5-aminoimidazole-4-carboxamide ribonucleoside. A specific method for activating AMP-activated protein kinase in intact cells?. Eur J Biochem.

[38] Zhou G, Myers R, Li Y, Chen Y, Shen X, Fenyk-Melody J (2001). Role of AMP-activated protein kinase in mechanism of metformin action. J Clin Invest.

[39] Grasl-Kraupp B, Ruttkay-Nedecky B, Koudelka H, Bukowska K, Bursch W, Schulte-Hermann R (1995). In situ detection of fragmented DNA (TUNEL assay) fails to discriminate among apoptosis, necrosis, and autolytic cell death: a cautionary note. Hepatology.

[40] Lin Y, Devin A, Rodriguez Y, Liu ZG (1999). Cleavage of the death domain kinase RIP by caspase-8 prompts TNF-induced apoptosis. Genes Dev.

[41] Feng S, Yang Y, Mei Y, Ma L, Zhu DE, Hoti N (2007). Cleavage of RIP3 inactivates its caspase-independent apoptosis pathway by removal of kinase domain. Cell Signal.

[42] Tummers B, Green DR (2017). Caspase-8: regulating life and death. Immunol Rev.

[43] Yuan J, Najafov A, Py BF (2016). Roles of caspases in necrotic cell death. Cell.

[44] Hughes MA, Powley IR, Jukes-Jones R, Horn S, Feoktistova M, Fairall L (2016). Co-operative and hierarchical binding of c-FLIP and caspase-8: a unified model defines how c-FLIP isoforms differentially control cell fate. Mol Cell.

[45] Pop C, Oberst A, Drag M, Van Raam BJ, Riedl SJ, Green DR (2011). FLIP(L) induces caspase 8 activity in the absence of interdomain caspase 8 cleavage and alters substrate specificity. Biochem J.

[46] Yan MX, Li YQ, Meng M, Ren HB, Kou Y (2006). Long-term high-fat diet induces pancreatic injuries via pancreatic microcirculatory disturbances and oxidative stress in rats with hyperlipidemia. Biochem Biophys Res Commun.

[47] Ge P, Luo Y, Okoye CS, Chen H, Liu J, Zhang G (2020). Intestinal barrier damage, systemic inflammatory response syndrome, and acute lung injury: A troublesome trio for acute pancreatitis. Biomed Pharmacother.

[48] de Oliveira C, Khatua B, Noel P, Kostenko S, Bag A, Balakrishnan B (2020). Pancreatic triglyceride lipase mediates lipotoxic systemic inflammation. J Clin Invest.

[49] Isenmann R, Rau B, Beger HG (2001). Early severe acute pancreatitis: characteristics of a new subgroup. Pancreas.

[50] Fu CY, Yeh CN, Hsu JT, Jan YY, Hwang TL (2007). Timing of mortality in severe acute pancreatitis: experience from 643 patients. World J Gastroenterol.

[51] Habtezion A, Gukovskaya AS, Pandol SJ (2019). Acute pancreatitis: a multifaceted set of organelle and cellular interactions. Gastroenterology.

[52] Kroemer G, Galluzzi L, Vandenabeele P, Abrams J, Alnemri ES, Baehrecke EH (2009). Classification of cell death: recommendations of the Nomenclature Committee on Cell Death 2009. Cell Death Differ.

[53] Chen X, He WT, Hu L, Li J, Fang Y, Wang X (2016). Pyroptosis is driven by non-selective gasdermin-D pore and its morphology is different from MLKL channel-mediated necroptosis. Cell Res.

[54] Ros U, Pena-Blanco A, Hanggi K, Kunzendorf U, Krautwald S, Wong WW (2017). Necroptosis execution is mediated by plasma membrane nanopores independent of calcium. Cell Rep.

[55] Linkermann A, Brasen JH, De Zen F, Weinlich R, Schwendener RA, Green DR (2012). Dichotomy between RIP1- and RIP3-mediated necroptosis in tumor necrosis factor-alpha-induced shock. Mol Med.

[56] Eguchi Y, Shimizu S, Tsujimoto Y (1997). Intracellular ATP levels determine cell death fate by apoptosis or necrosis. Cancer Res.

[57] Tsujimoto Y (1997). Apoptosis and necrosis: intracellular ATP level as a determinant for cell death modes. Cell Death Differ.

[58] Vanden BT, Linkermann A, Jouan-Lanhouet S, Walczak H, Vandenabeele P (2014). Regulated necrosis: the expanding network of non-apoptotic cell death pathways. Nat Rev Mol Cell Biol.

[59] Wittkopf N, Gunther C, Martini E, He G, Amann K, He YW (2013). Cellular FLICE-like inhibitory protein secures intestinal epithelial cell survival and immune homeostasis by regulating caspase-8. Gastroenterology.

[60] Yang ZH, Wu XN, He P, Wang X, Wu J, Ai T (2020). A non-canonical PDK1-RSK signal diminishes pro-caspase-8-mediated necroptosis blockade. Mol Cell.

[61] Dolai S, Liang T, Orabi AI, Holmyard D, Xie L, Greitzer-Antes D (2018). Pancreatitis-induced depletion of syntaxin 2 promotes autophagy and increases basolateral exocytosis. Gastroenterology.

[62] Kusske AM, Rongione AJ, Ashley SW, McFadden DW, Reber HA (1996). Interleukin-10 prevents death in lethal necrotizing pancreatitis in mice. Surgery.

