# Supplementary material for: Activation of AMPK ameliorates acute severe pancreatitis by suppressing pancreatic acinar cell necroptosis in obese mice models
Source: Cell Death Discov. 2023 Sep 30;9:363. doi: 10.1038/s41420-023-01655-z (PMC10542799; doi:10.1038/s41420-023-01655-z)
Supplement: Supplementary file 1 — Original western blots [file 41420_2023_1655_MOESM1_ESM.pdf]

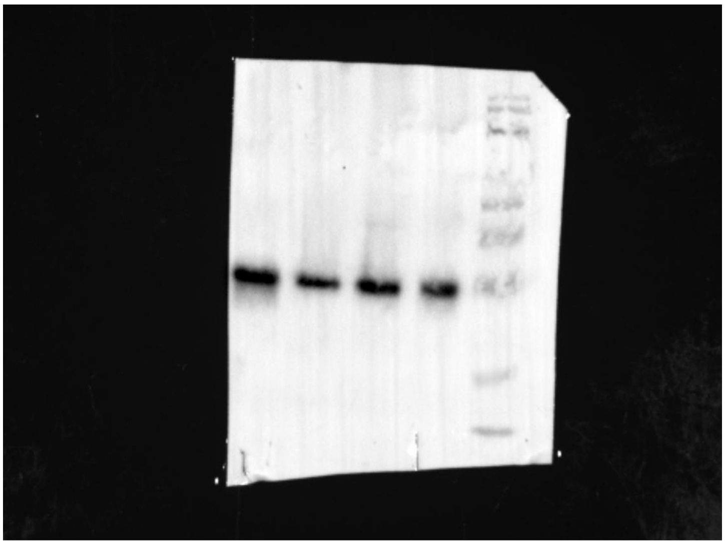

Full unedited gel for fig.2A C57 and DIO mcie GAPDH

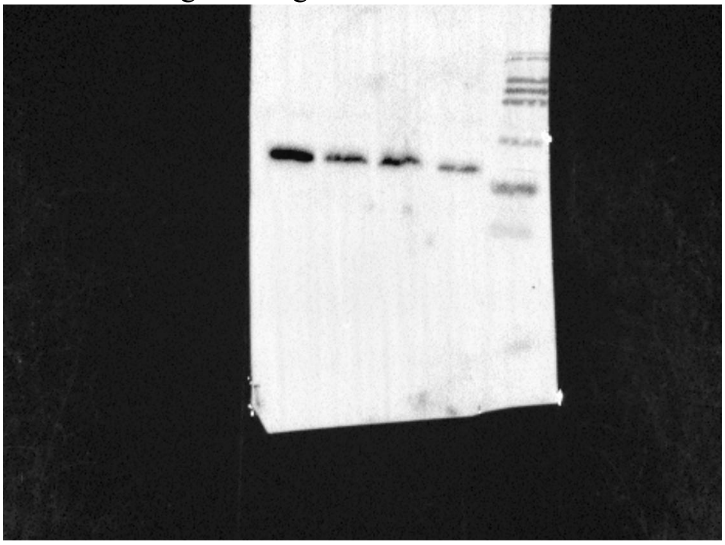

Full unedited gel for fig.2A C57 and DIO mcie p-AMPK

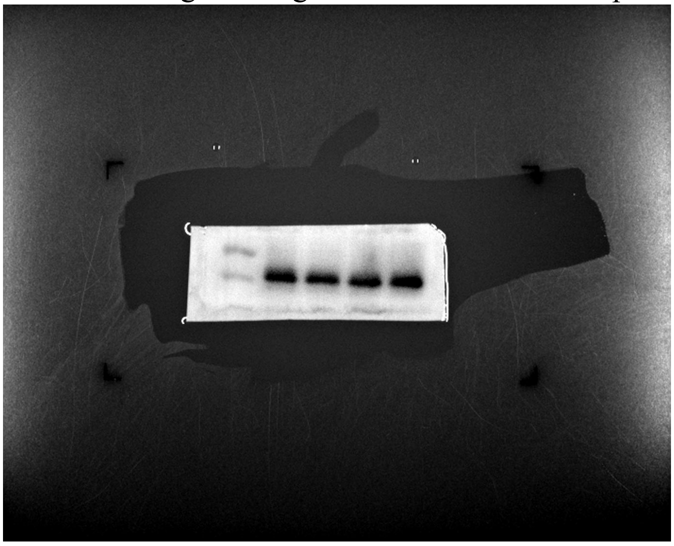

Full unedited gel for fig.2A C57 and ob mice GAPDH

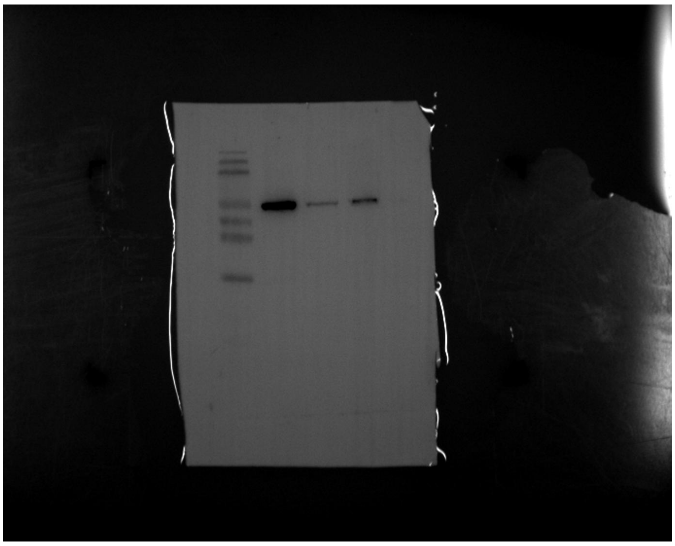

Full unedited gel for fig.2A C57 and ob mice p-AMPK

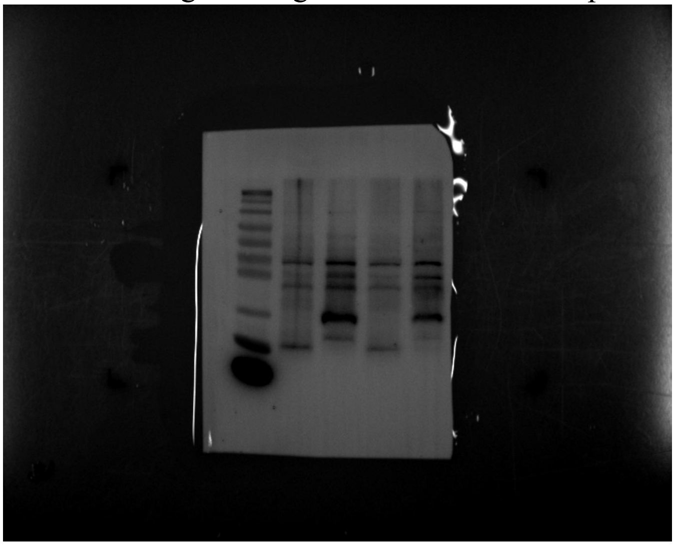

Full unedited gel for fig .5A   cell caspase-3

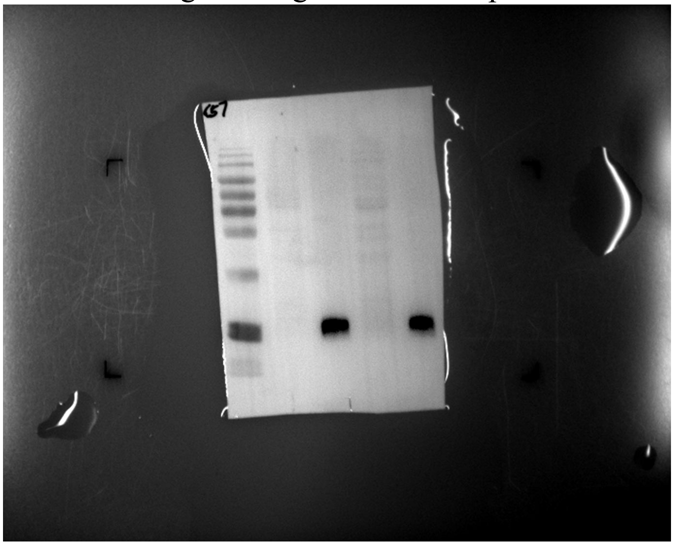

Full unedited gel for fig .5A   cell c-Caspase-8

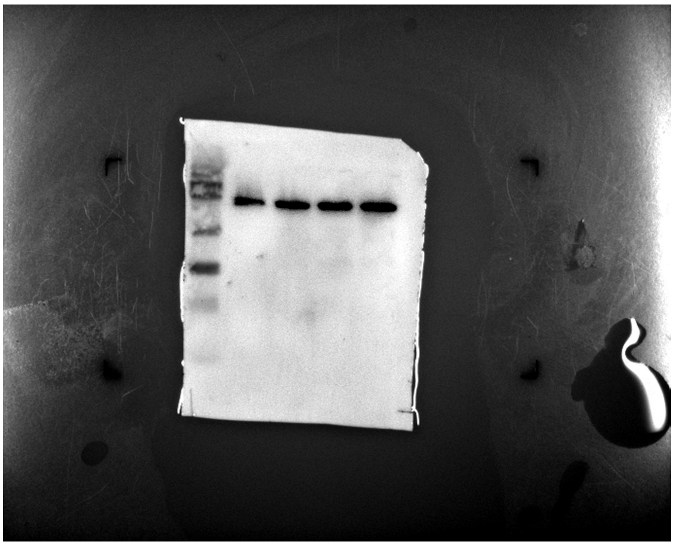

Full unedited gel for fig .5A   cell GAPDH

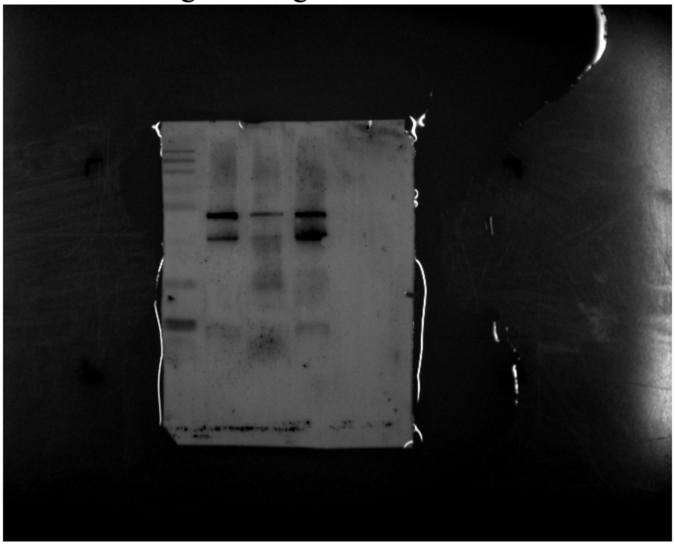

Full unedited gel for fig .5A   cell p-AMPK

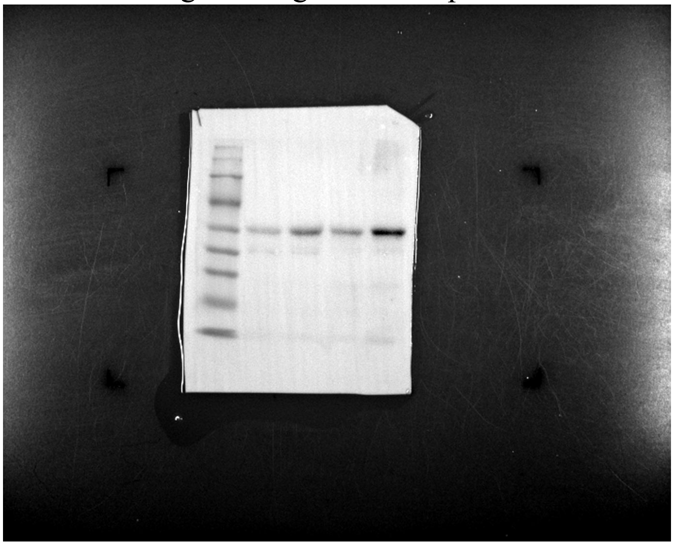

Full unedited gel for fig .5A   cell p-MLKL

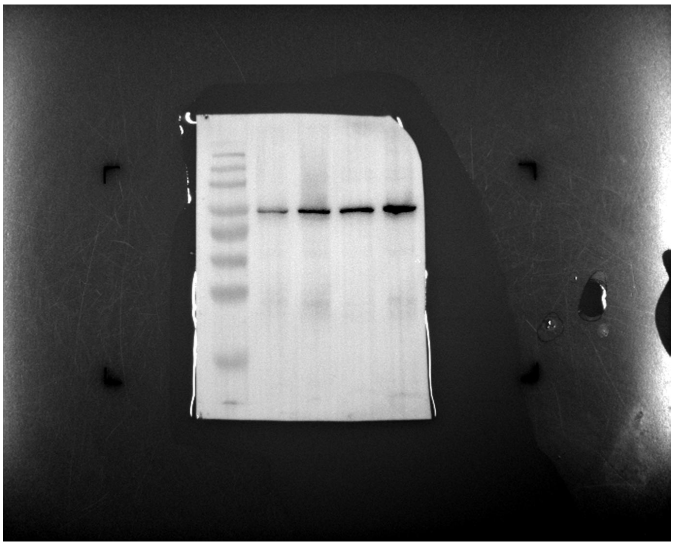

Full unedited gel for fig .5A cell p-RIPK1

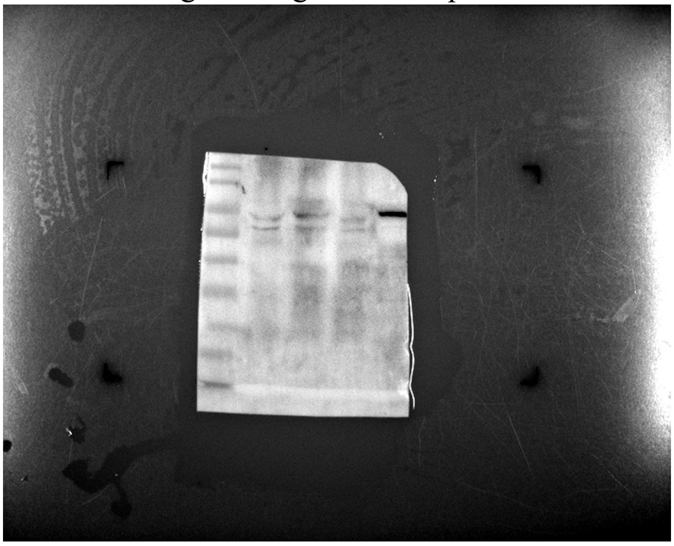

Full unedited gel for fig .5A cell p-RIPK3

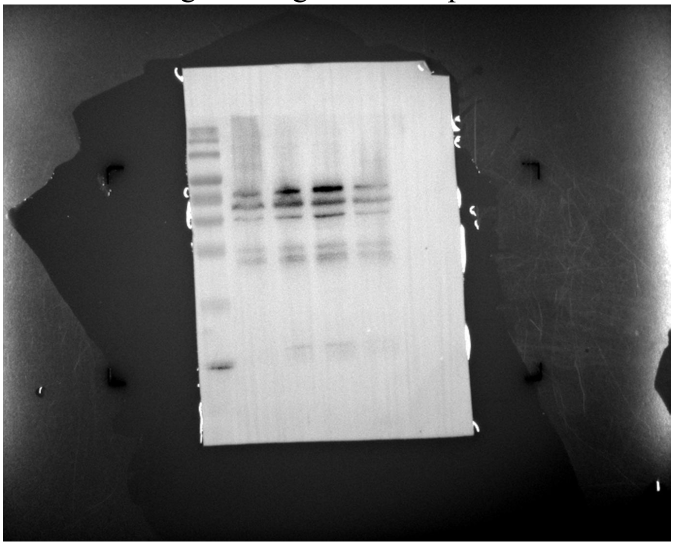

Full unedited gel for fig .5A cell pro-Caspase-8

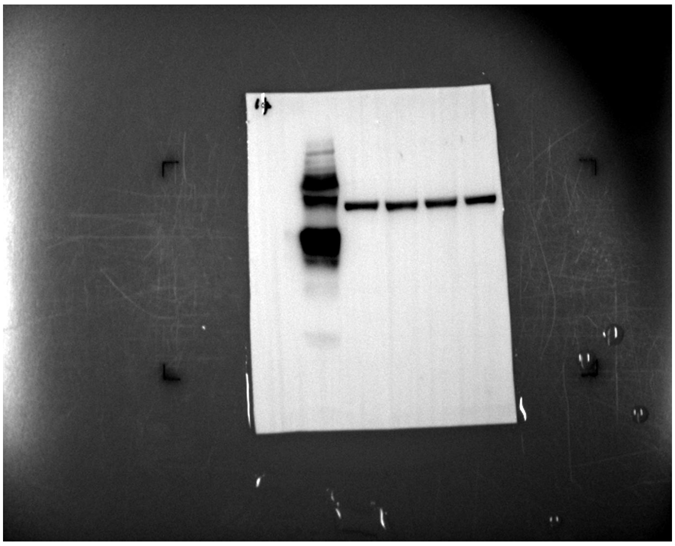

Full unedited gel for fig .5A cell RIPK1

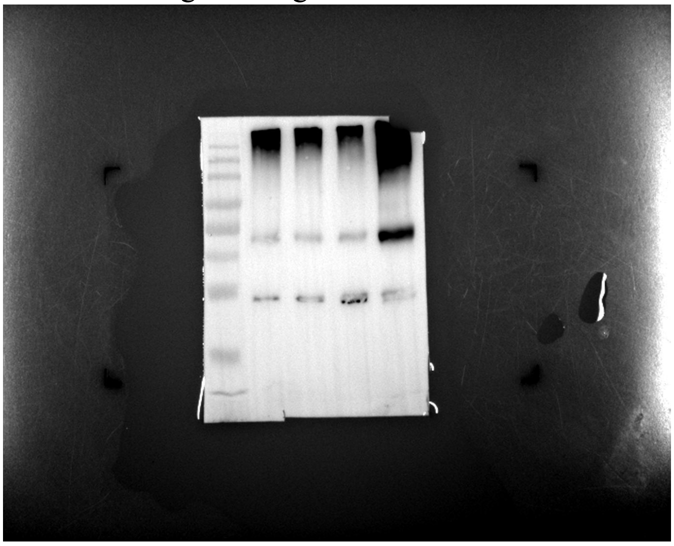

Full unedited gel for fig .5A cell RIPK3

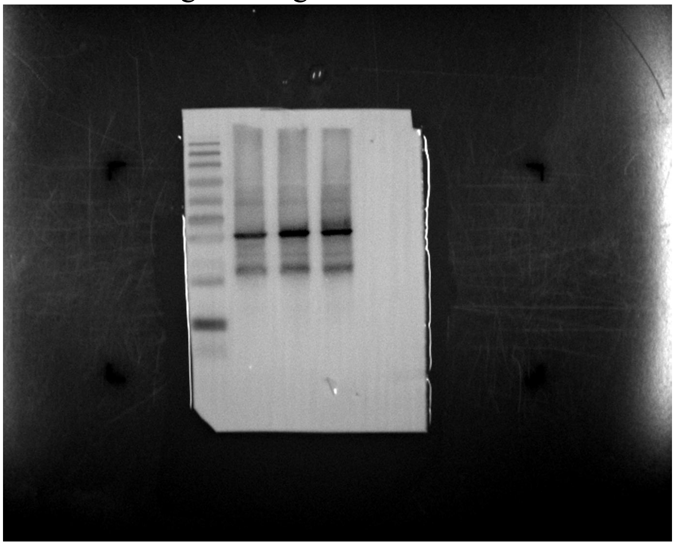

Full unedited gel for fig .5A ob mcie GAPDH

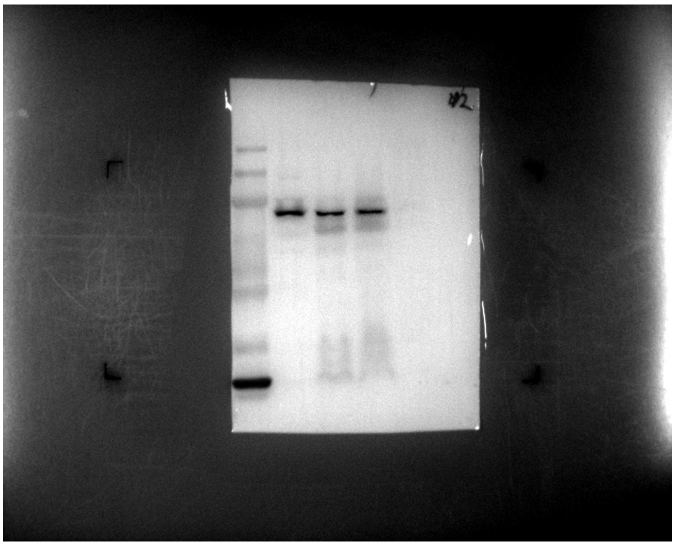

Full unedited gel for fig .5A ob mcie RIPK1

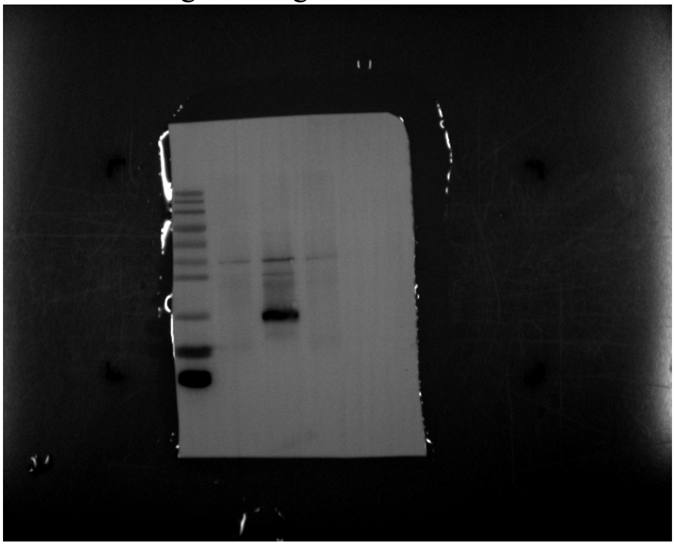

Full unedited gel for fig .5A ob mice Caspase-3(5s)

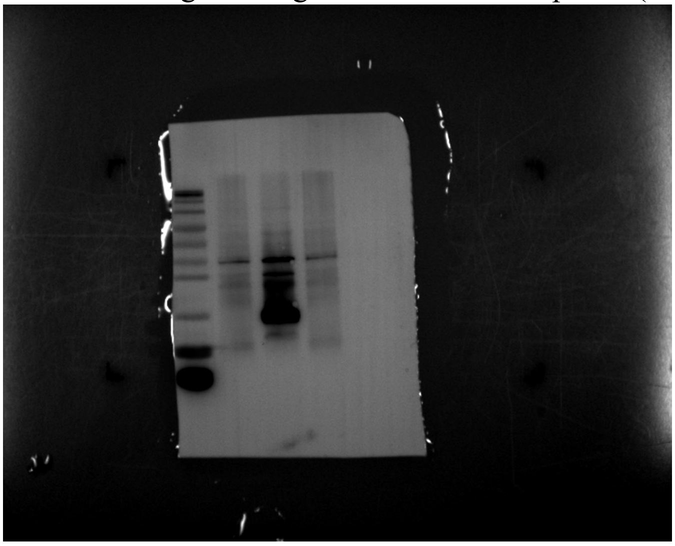

Full unedited gel for fig .5A ob mice Caspase-3(10s)

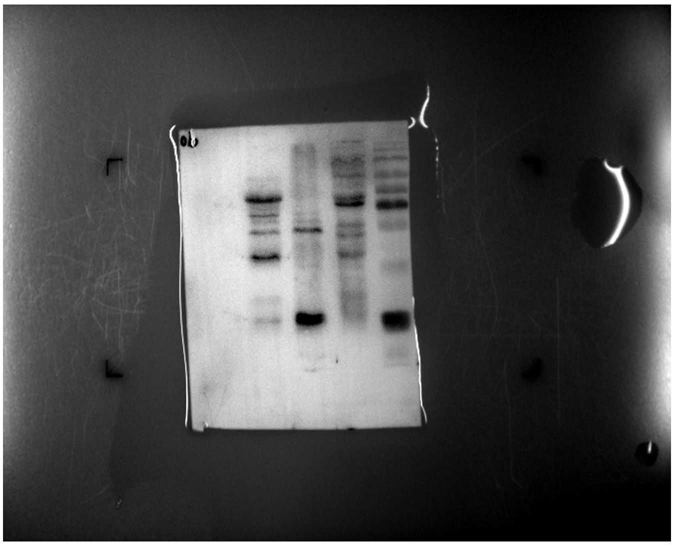

Full unedited gel for fig .5A   ob mice Caspase-8

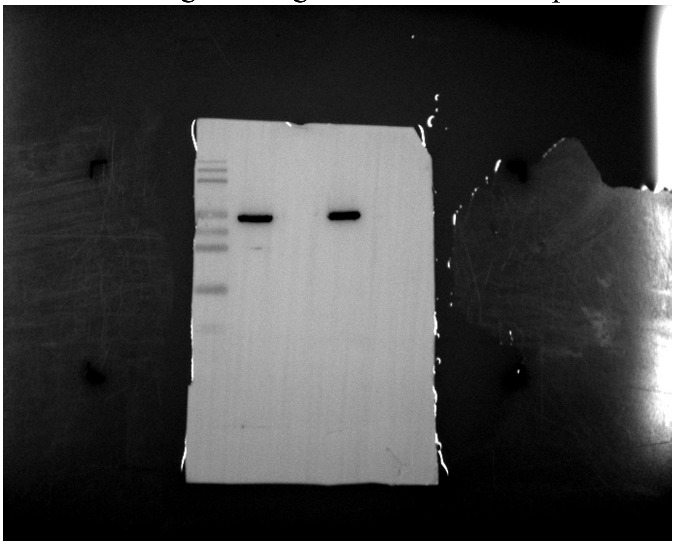

Full unedited gel for fig .5A   ob mice p-AMPK

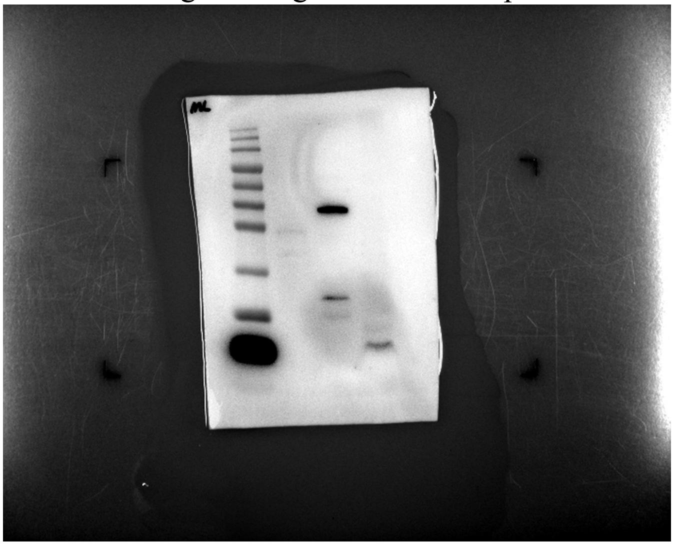

Full unedited gel for fig .5A   ob mice p-MLKL

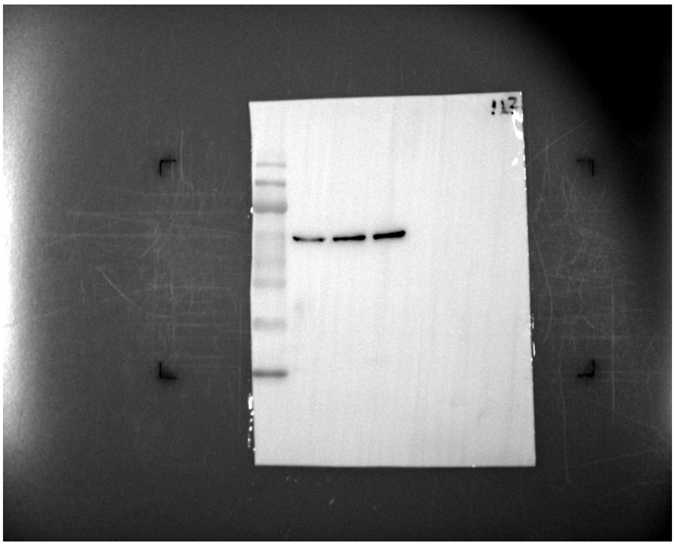

Full unedited gel for fig .5A   ob mice p-RIPK1

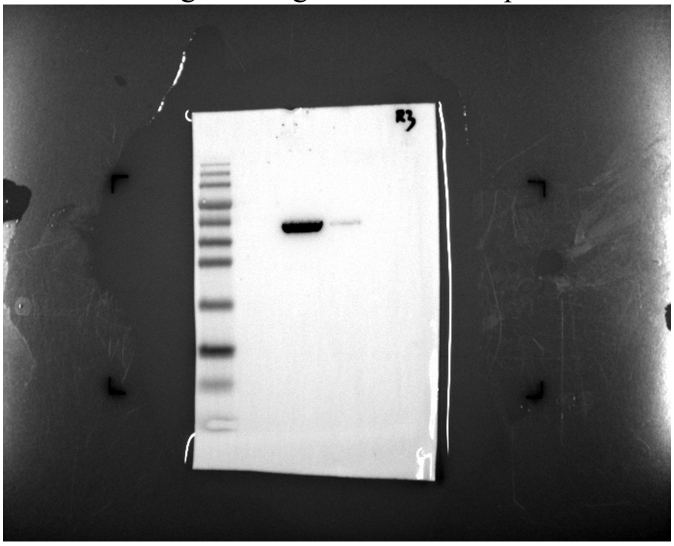

Full unedited gel for fig .5A   ob mice p-RIPK3

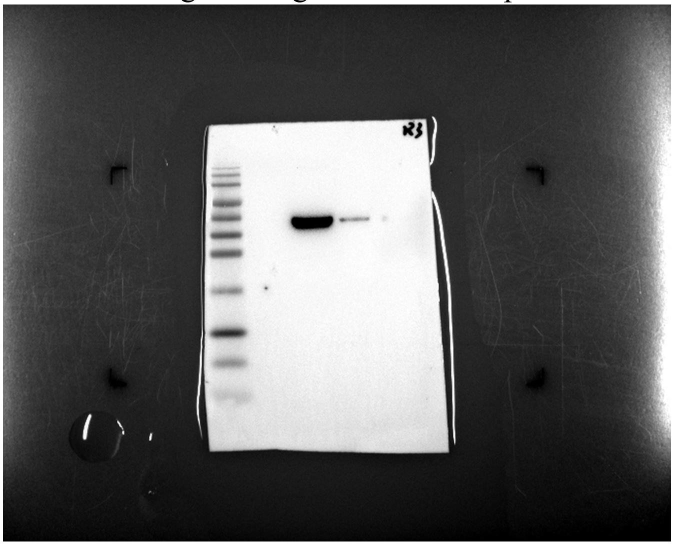

Full unedited gel for fig .5A   ob mice RIPK3

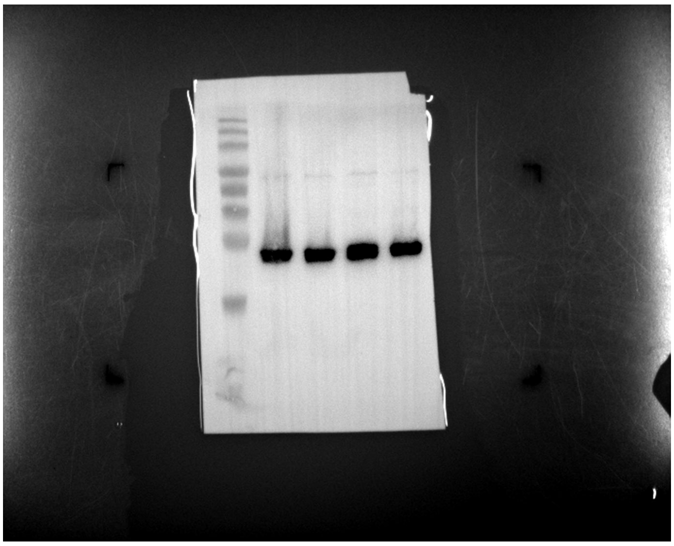
Full unedited gel for fig .6A sicaspase-8： GAPDH

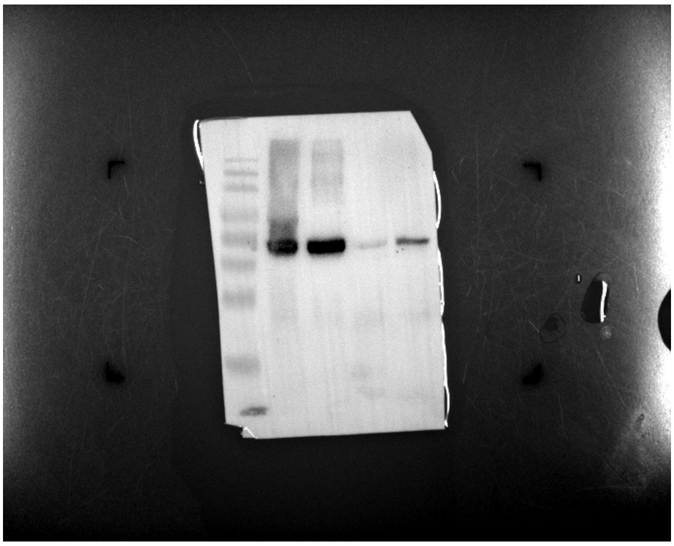
Full unedited gel for fig .6A sicaspase-8： pro-Caspase8

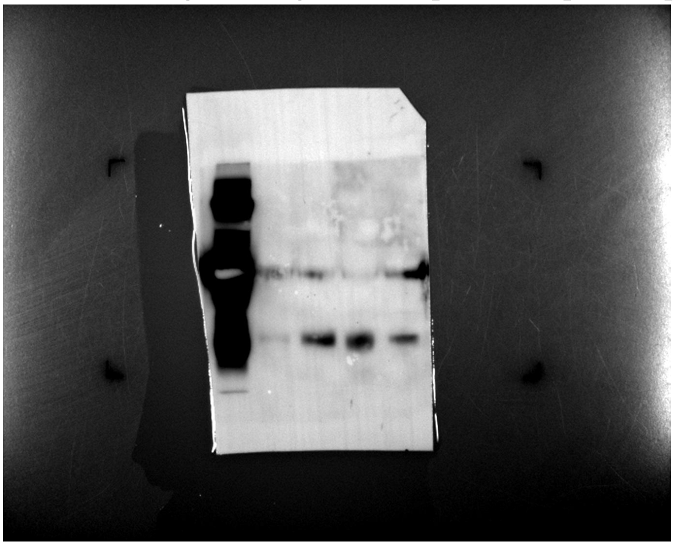
Full unedited gel for fig .6H sicaspase-8 c-Caspase-8

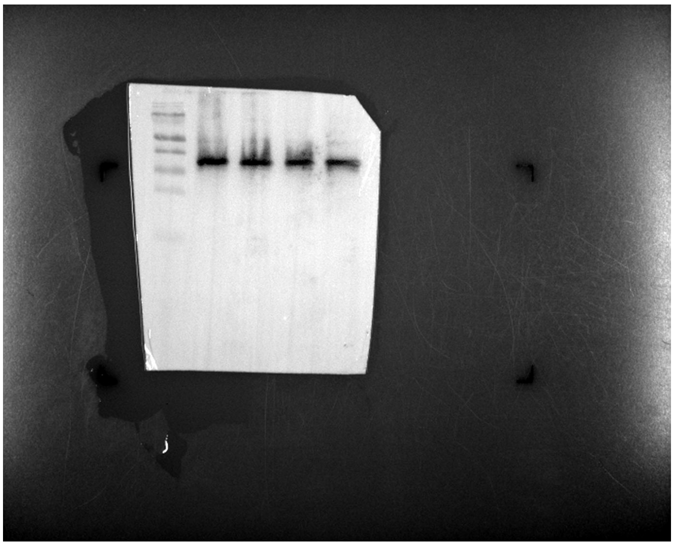

Full unedited gel for fig .6H sicaspase-8 GAPDH

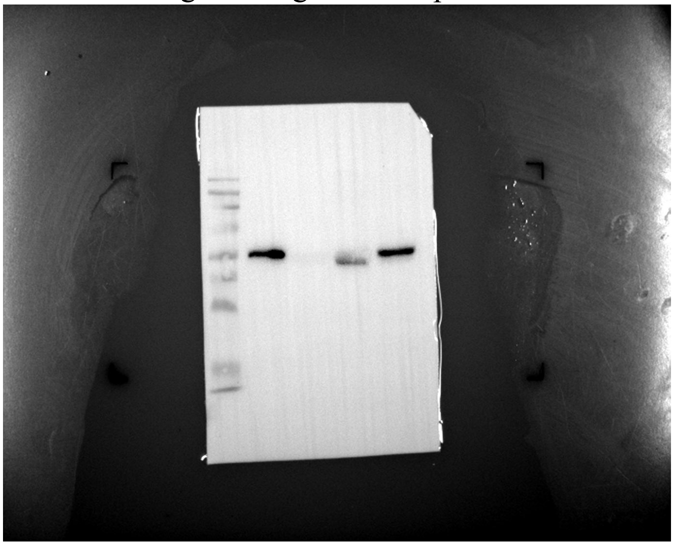

Full unedited gel for fig .6H sicaspase-8 p-AMPK

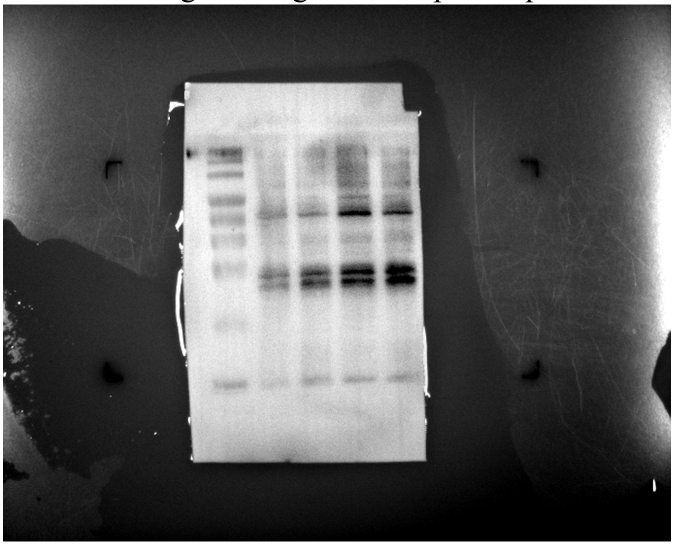

Full unedited gel for fig .6H sicaspase-8 p-MLKL

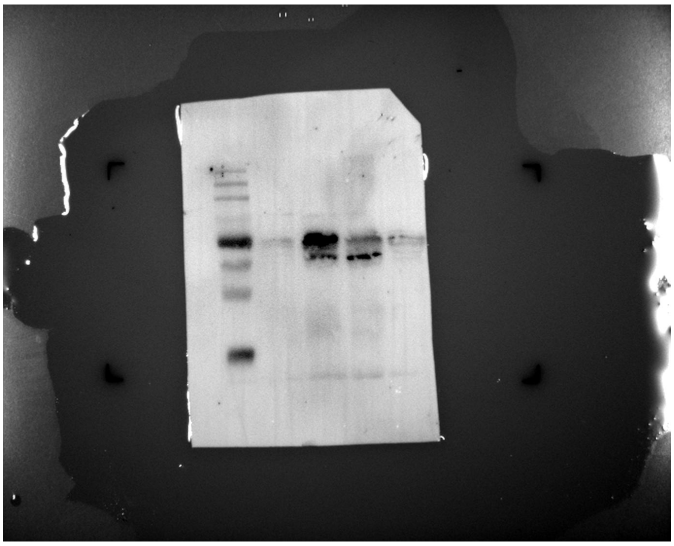

Full unedited gel for fig .6H sicaspase-8 pro-Caspase-8

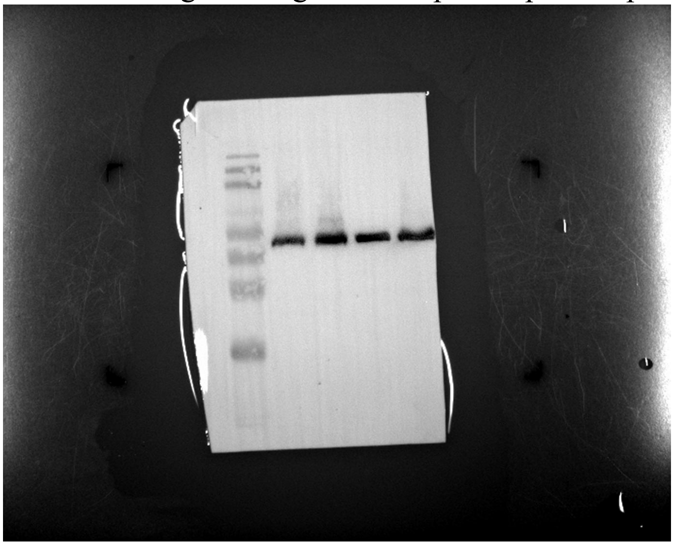

Full unedited gel for fig .6H sicaspase-8 RIPK1

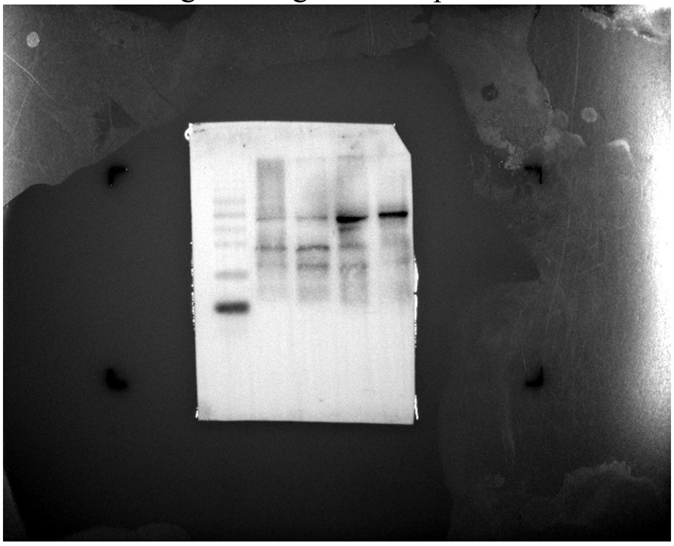

Full unedited gel for fig .6H sicaspase-8 RIPK3

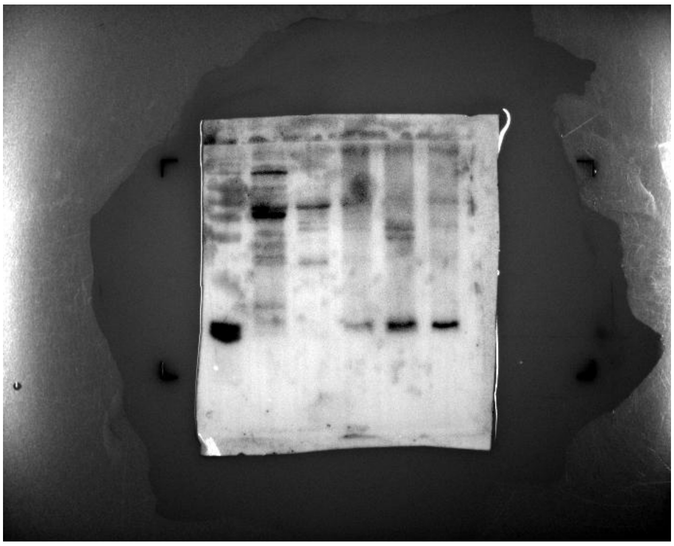

Full unedited gel for fig.7C CHX CER caspase-8

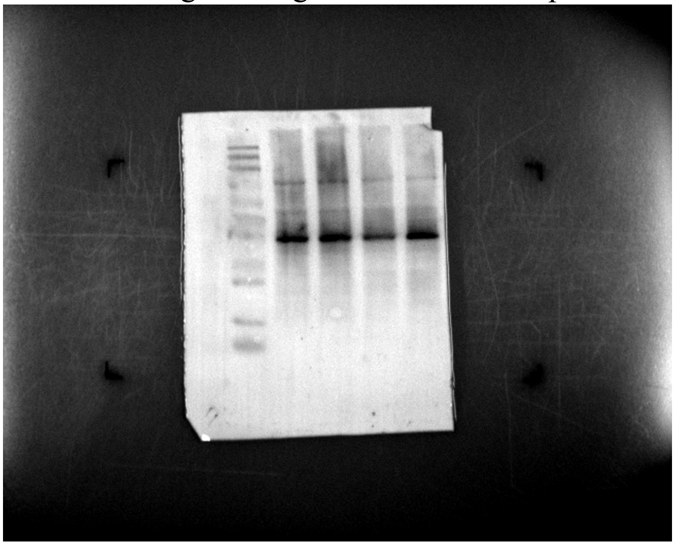

Full unedited gel for fig.7C CHX CER GAPDH

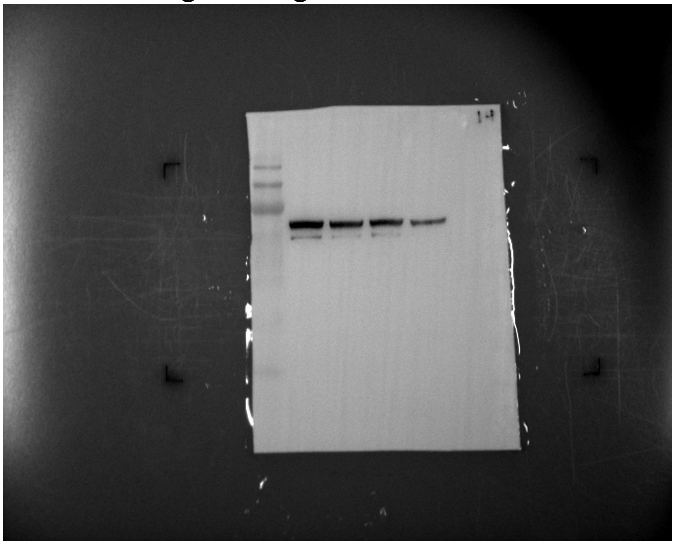

Full unedited gel for fig.7C CHX CER+AICAR caspase-8

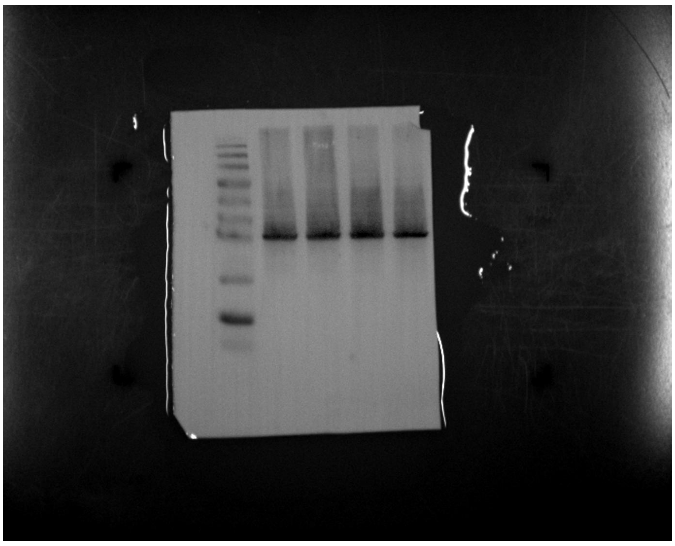

Full unedited gel for fig.7C CHX CER+AICAR GAPDH

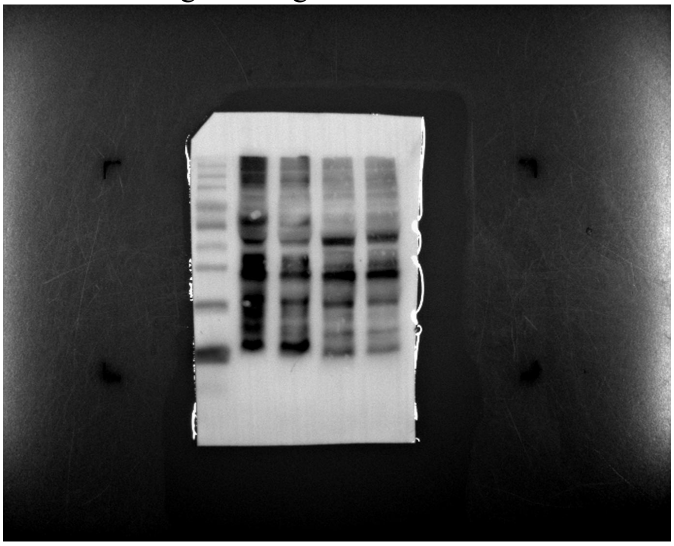

Full unedited gel for fig.7E MG132 caspase-8

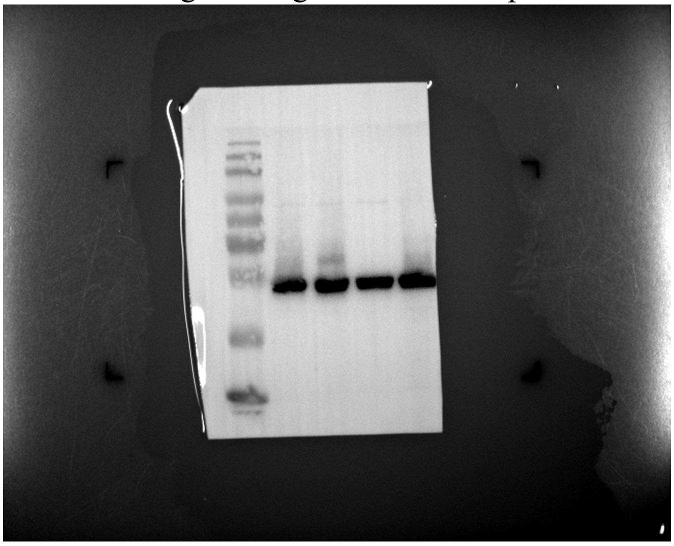

Full unedited gel for fig.7E MG132 GAPDH

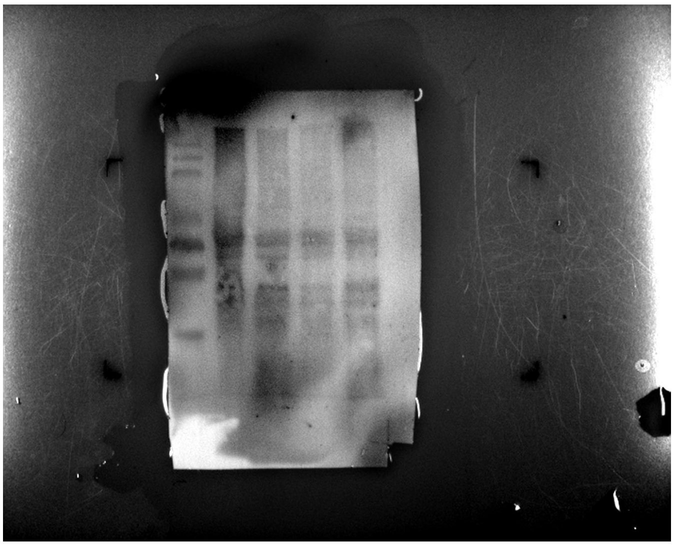

Full unedited gel for fig.7B IgG caspase-8

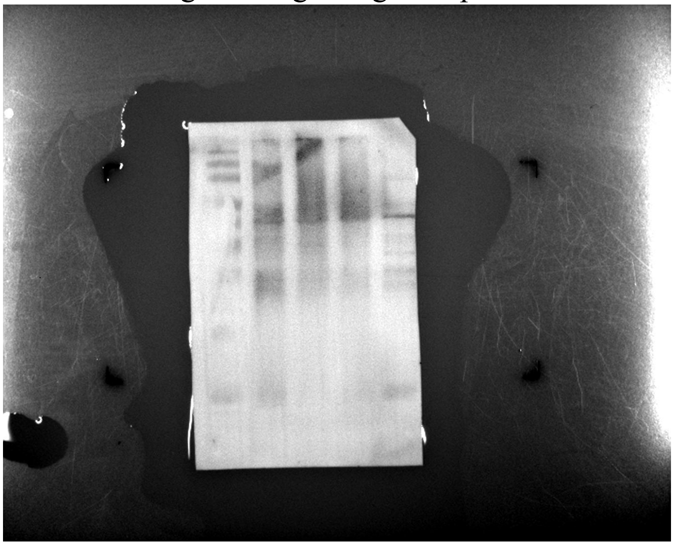

Full unedited gel for fig.7B IgG p-AMPK

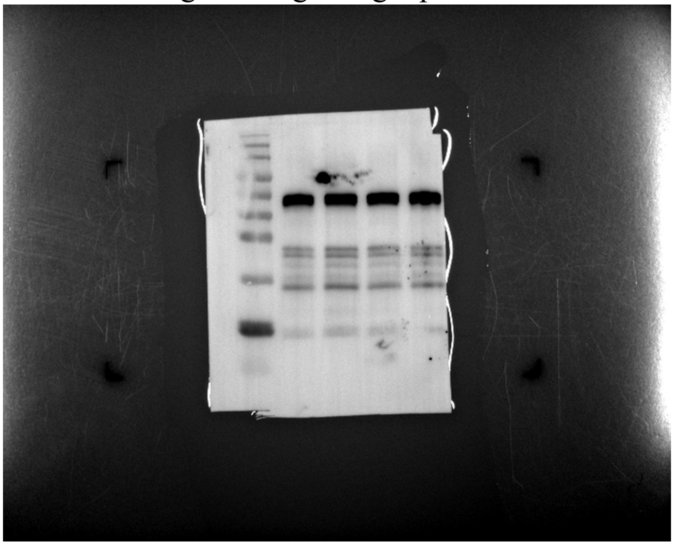

Full unedited gel for fig.7B Input caspae-8

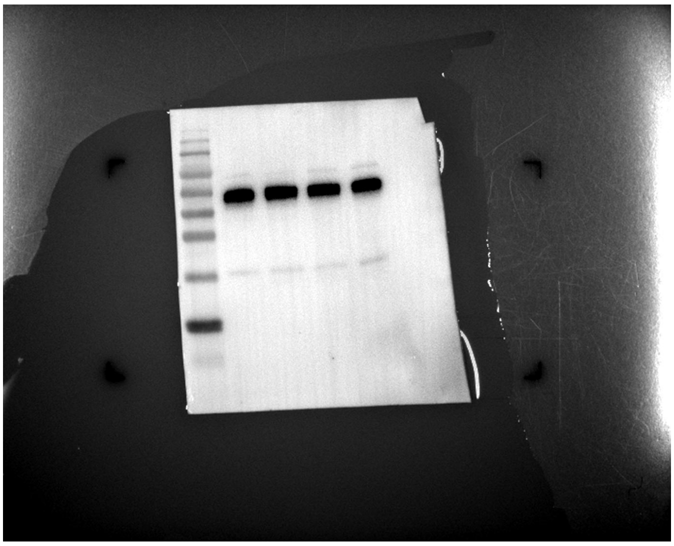

Full unedited gel for fig.7B Input p-AMPK

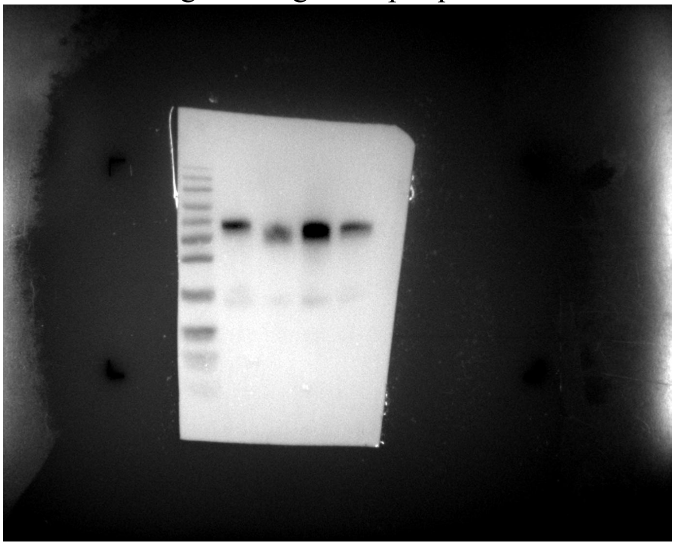

Full unedited gel for fig.7B IP caspase-8

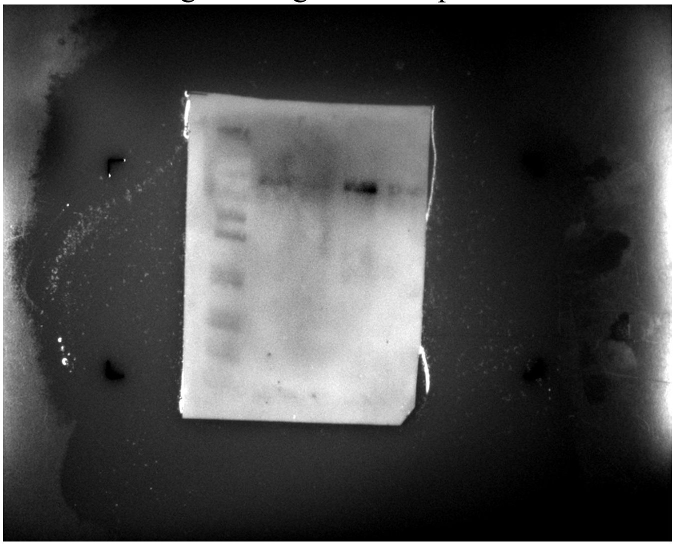

Full unedited gel for fig.7B IP p-AMPK

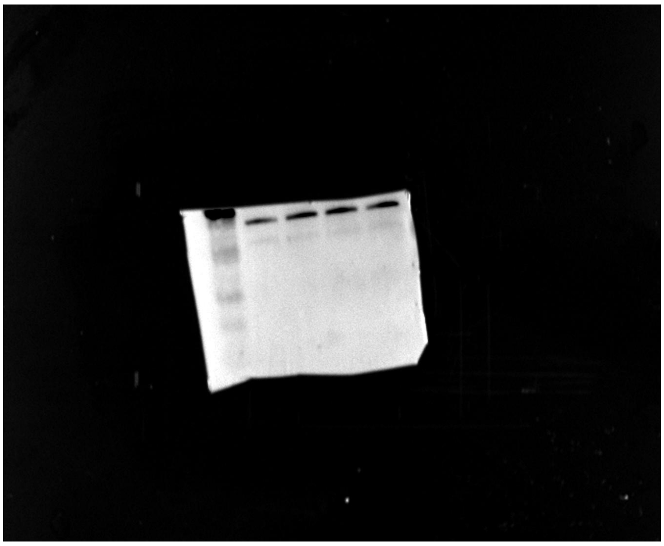

R2 Full unedited gel for fig.2A C57 and DIO mcie GAPDH

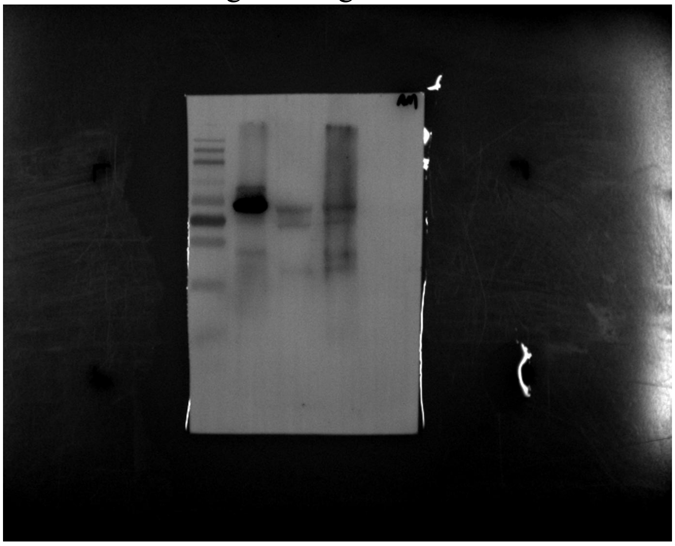

R2 Full unedited gel for fig.2A C57 and DIO mcie p-AMPK

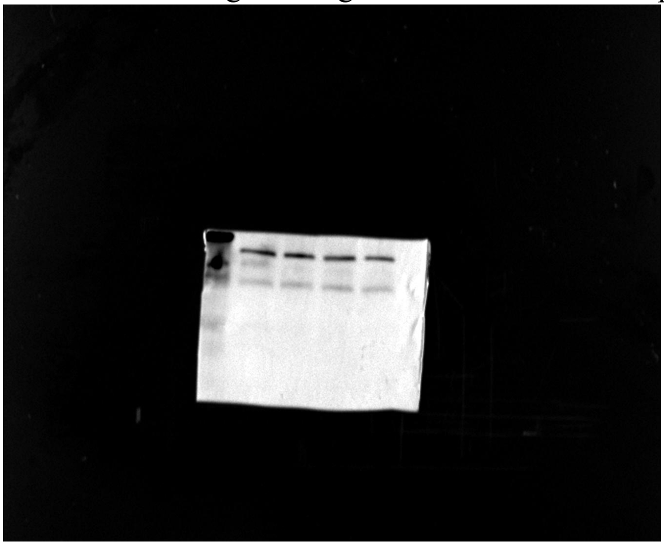

R3 Full unedited gel for fig.2A C57 and DIO mcie GAPDH

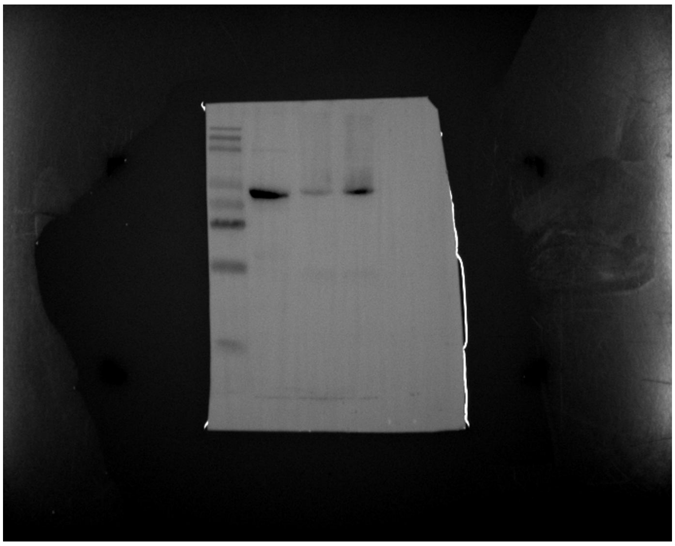

R3 Full unedited gel for fig.2A C57 and DIO mcie p-AMPK

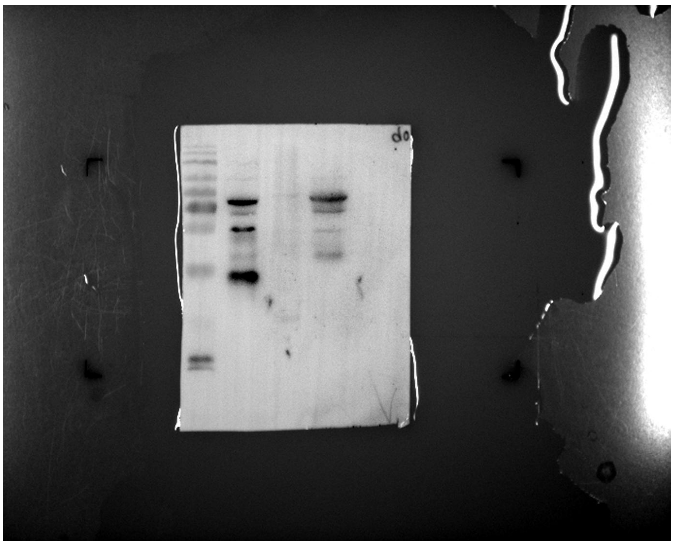

R2 Full unedited gel for fig.2A C57 and ob mice p-AMPK

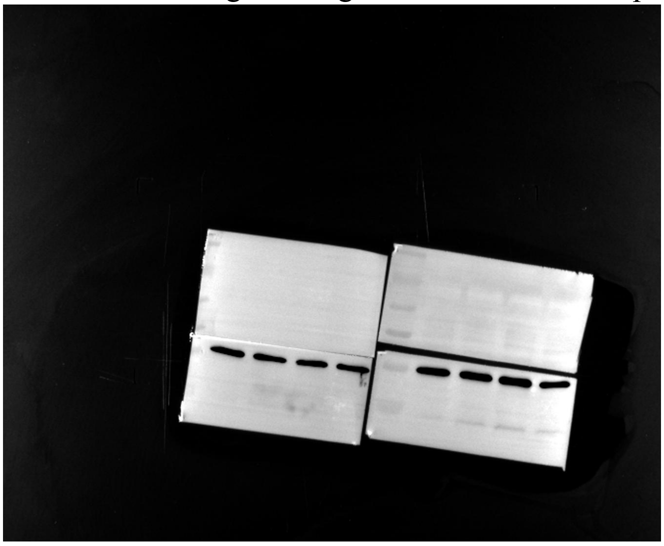

R2+R3 Full unedited gel for fig.2A C57 and ob mice GAPDH

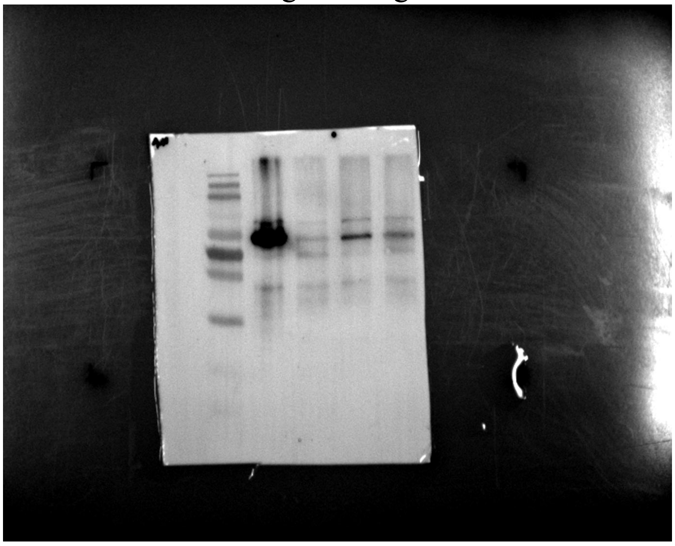

R3 Full unedited gel for fig.2A C57 and ob mice p-AMPK

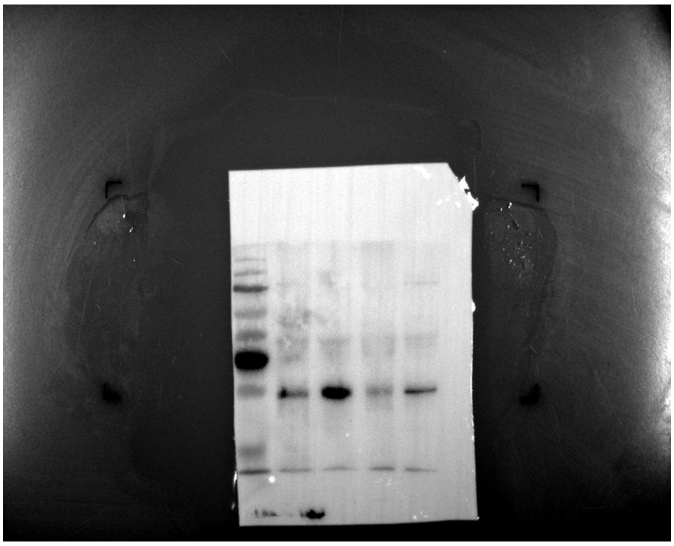

R2 Full unedited gel for fig .5A   cell caspase-3

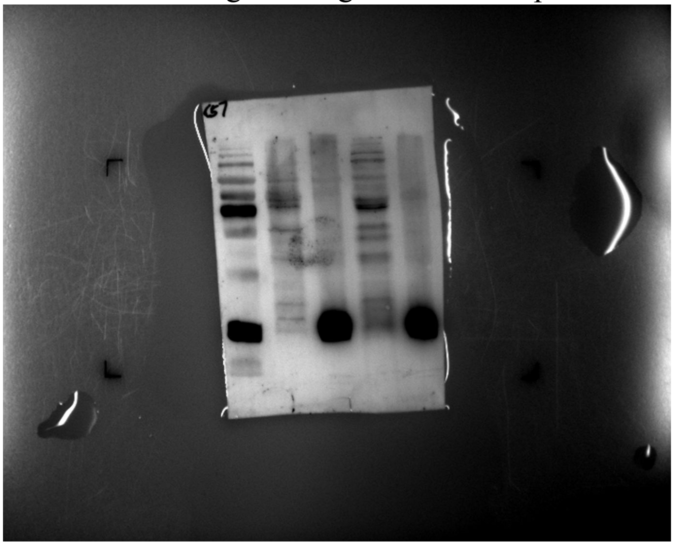

R2 Full unedited gel for fig .5A   cell caspase-8

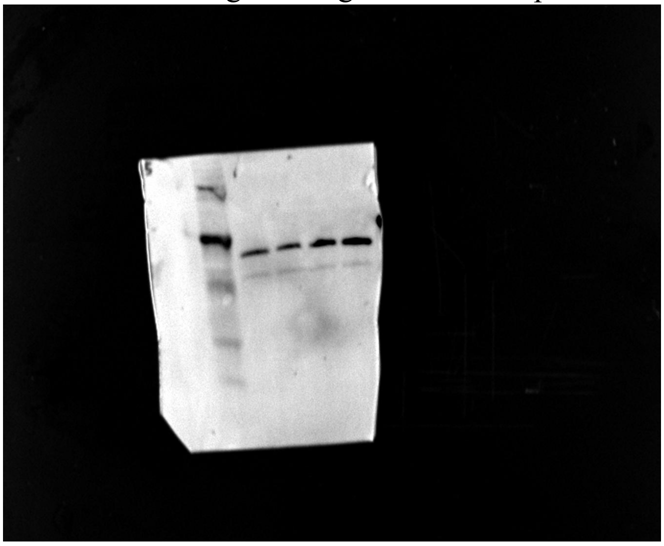

R2 Full unedited gel for fig .5A   cell GAPDH

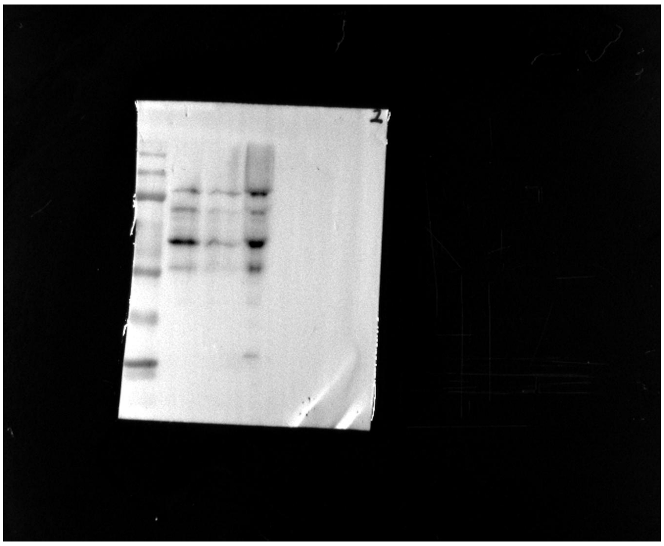

R2 Full unedited gel for fig .5A   cell p-AMPK

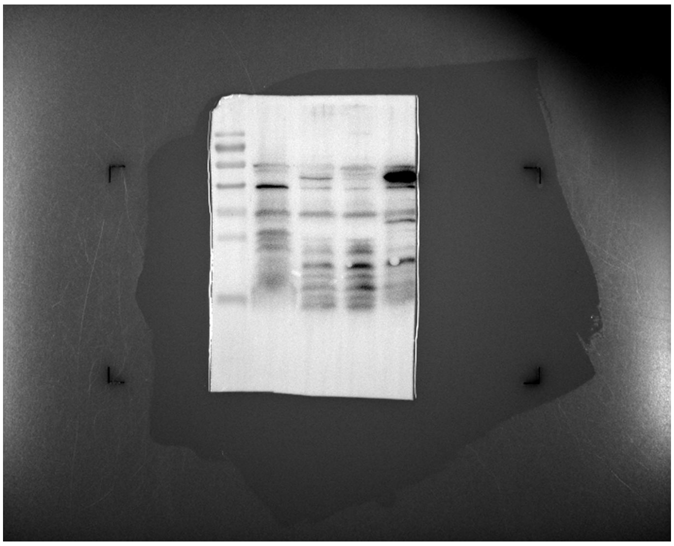

R2 Full unedited gel for fig .5A   cell p-MLKL

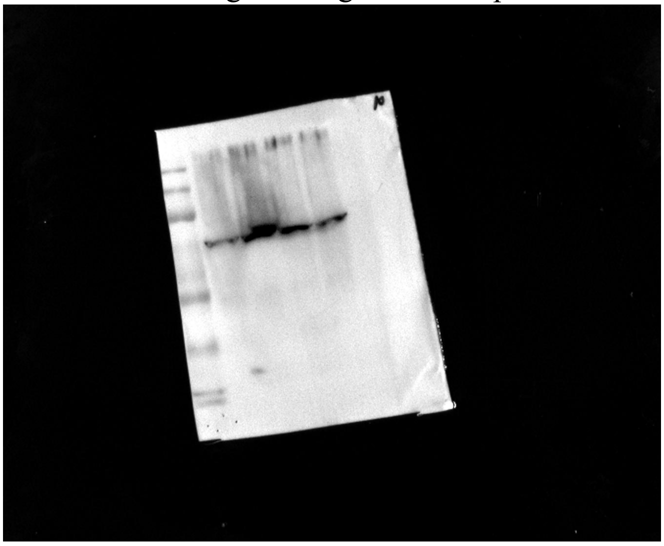

R2 Full unedited gel for fig .5A   cell p-RIPK1

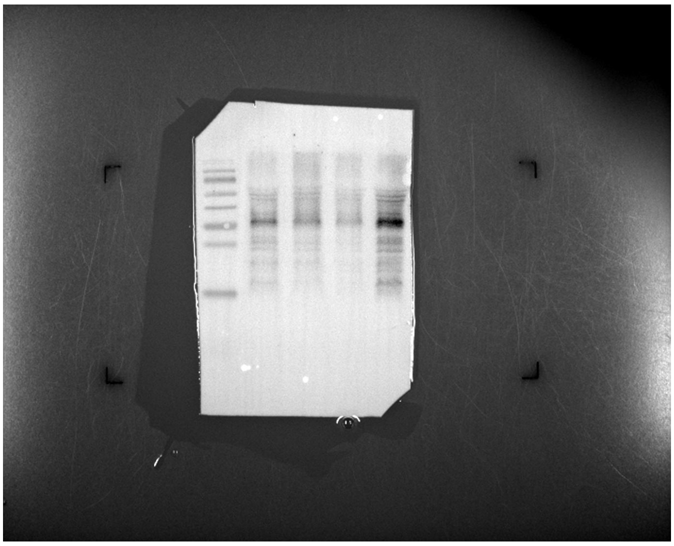

R2 Full unedited gel for fig .5A cell p-RIPK3

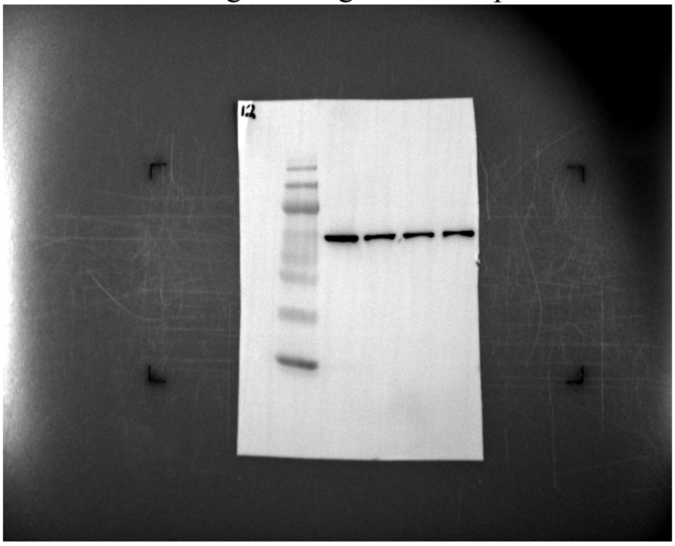

R2 Full unedited gel for fig .5A cell RIPK1

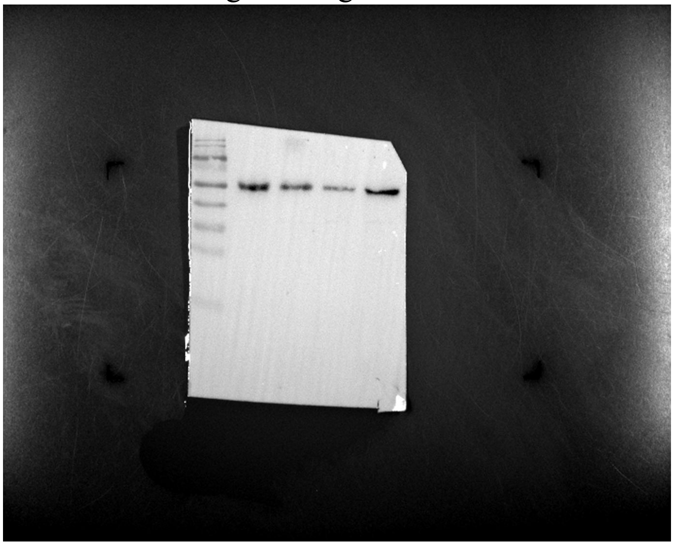

R2 Full unedited gel for fig .5A cell RIPK3

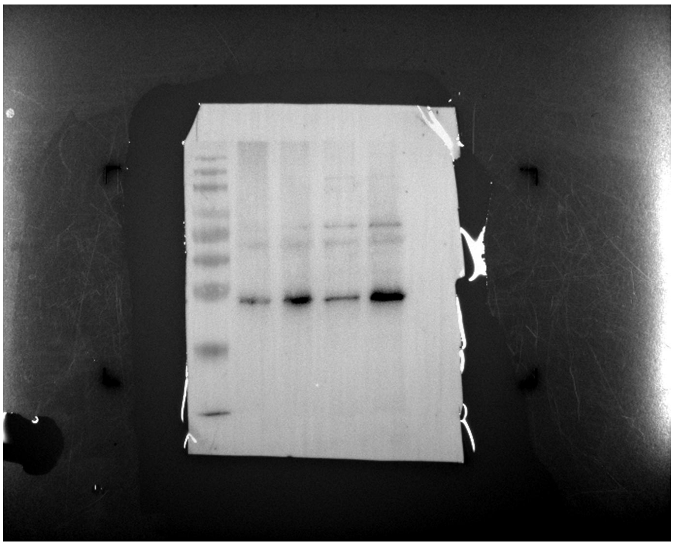

R3 Full unedited gel for fig .5A   cell caspase-3

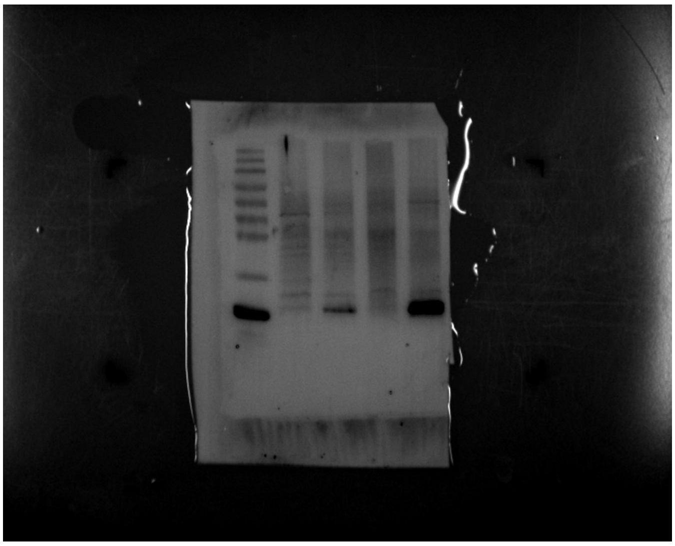

R3 Full unedited gel for fig .5A   cell c-Caspase-8

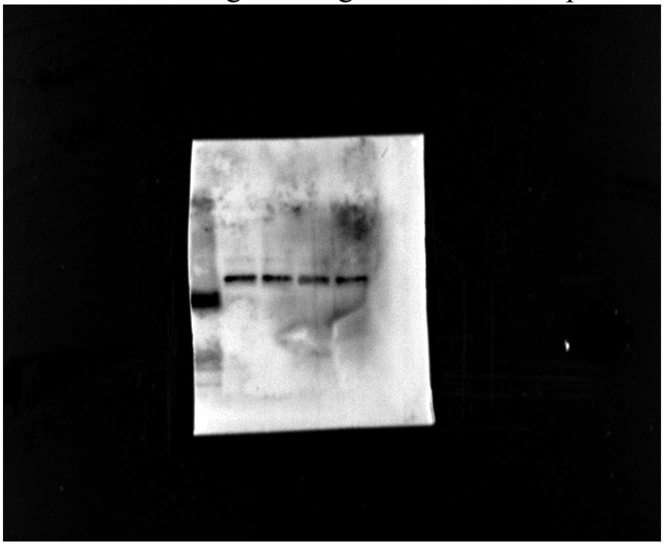

R3 Full unedited gel for fig .5A   cell GAPDH

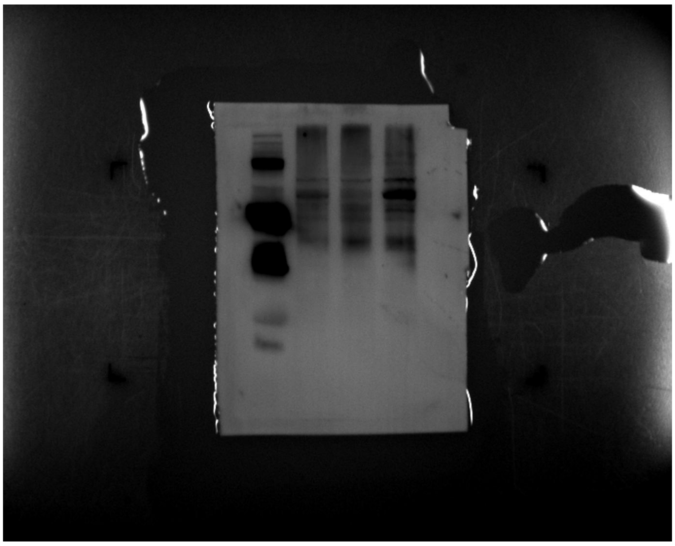

R3 Full unedited gel for fig .5A   cell p-AMPK

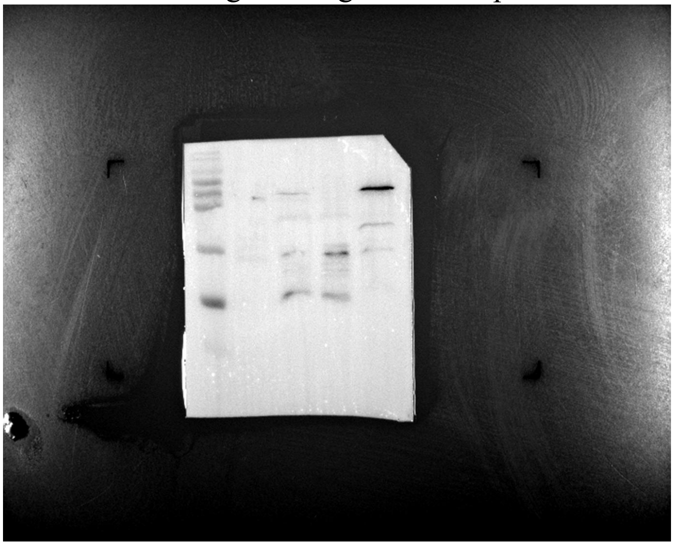

R3 Full unedited gel for fig .5A   cell p-MLKL

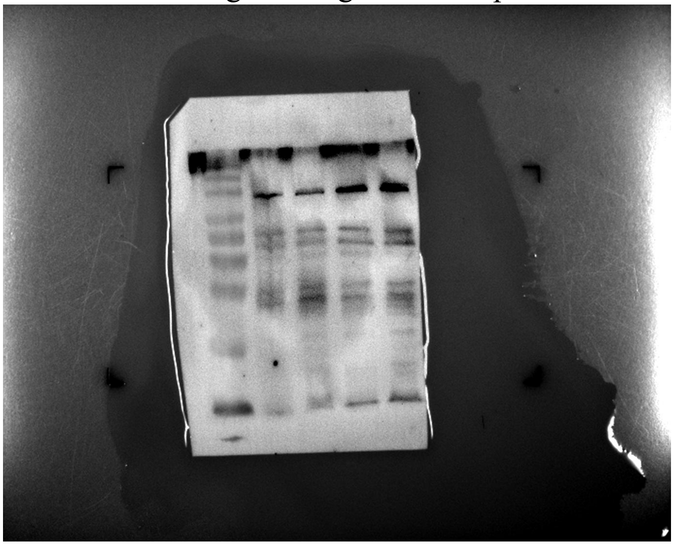

R3 Full unedited gel for fig .5A   cell p-RIPK1

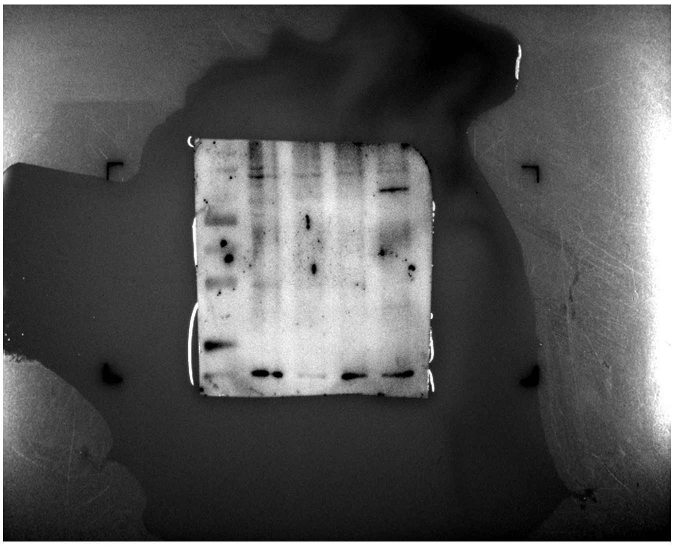

R3 Full unedited gel for fig .5A   cell p-RIPK3

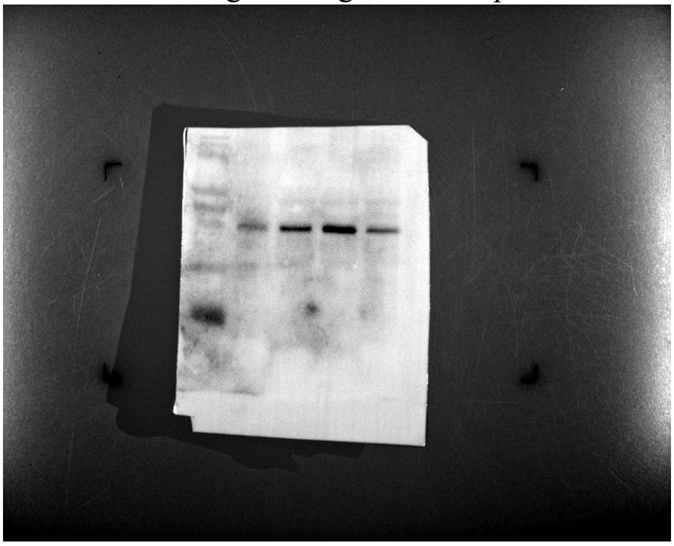

R3 Full unedited gel for fig .5A   cell pro-Caspase-8

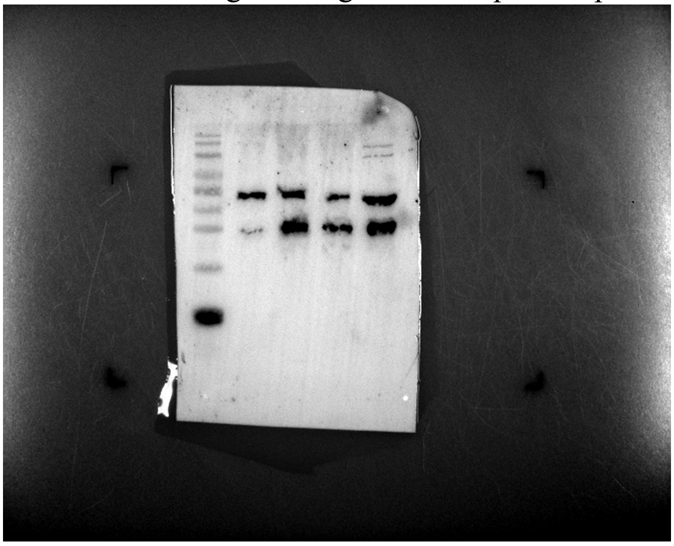

R3 Full unedited gel for fig .5A   cell RIPK1

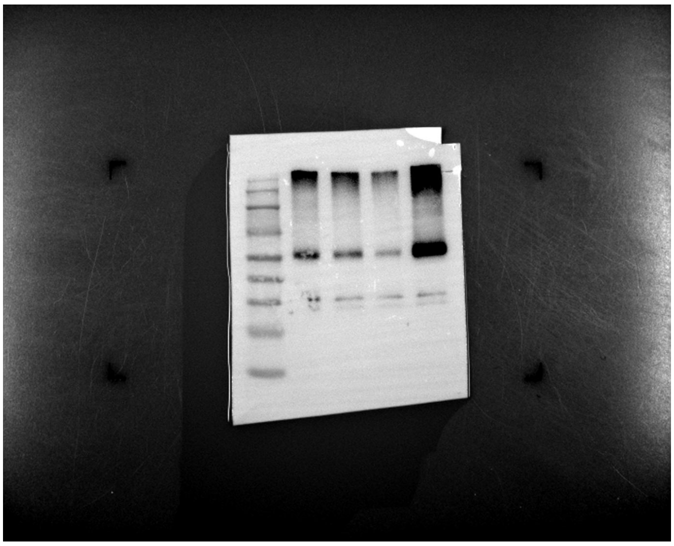

R3 Full unedited gel for fig .5A   cell RIPK3

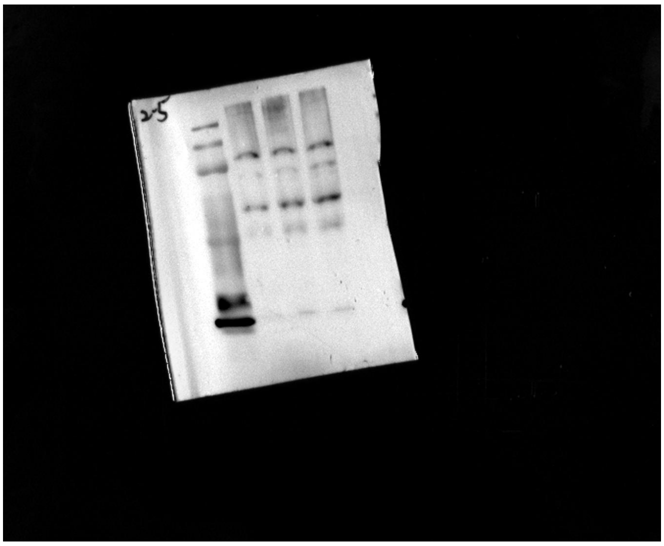

R2 Full unedited gel for fig .5A   ob mcie RIPK1

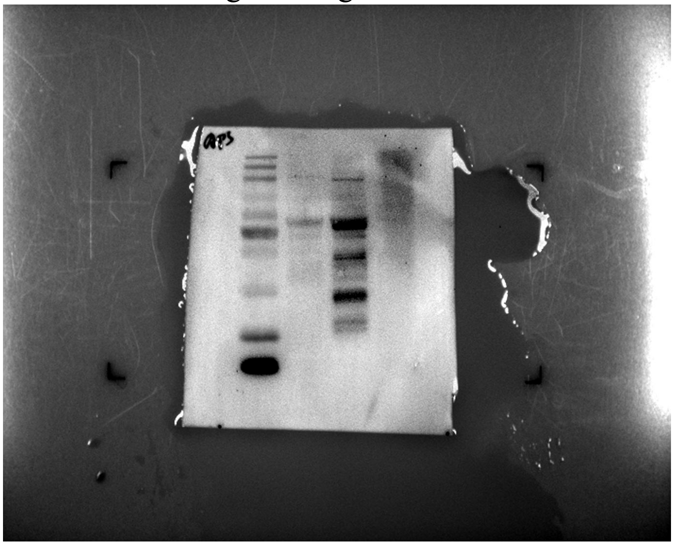

R2 Full unedited gel for fig .5A   ob mice Caspase-3(10s)

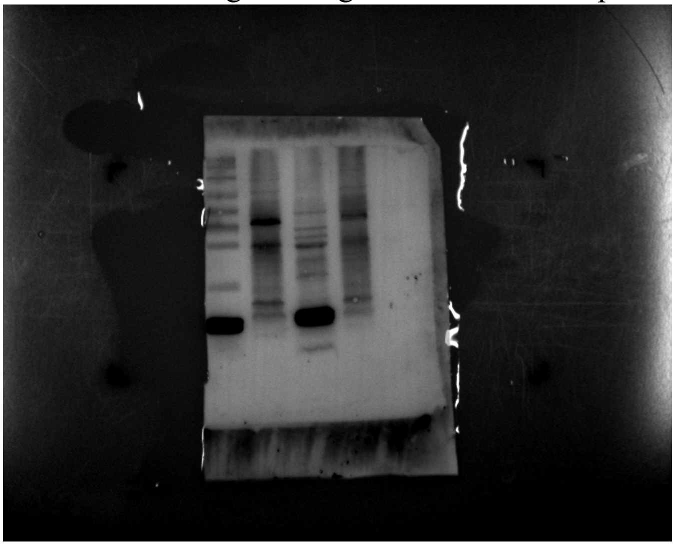

R2 Full unedited gel for fig .5A   ob mice Caspase-8

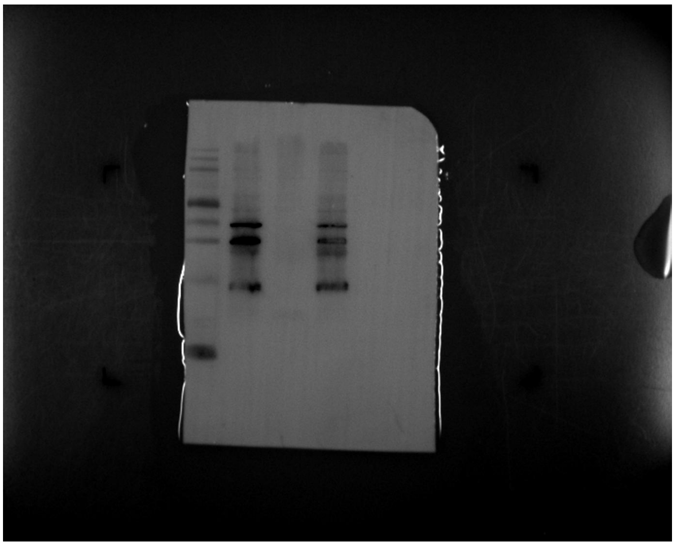

R2 Full unedited gel for fig .5A   ob mice p-AMPK

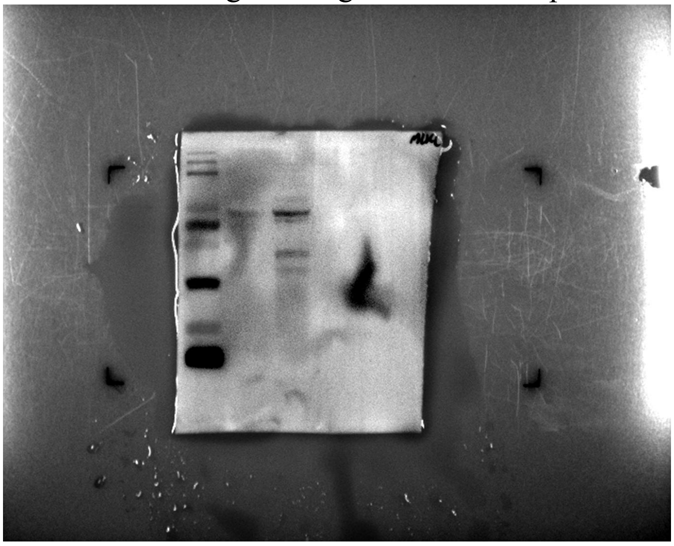

R2 Full unedited gel for fig .5A   ob mice p-MLKL

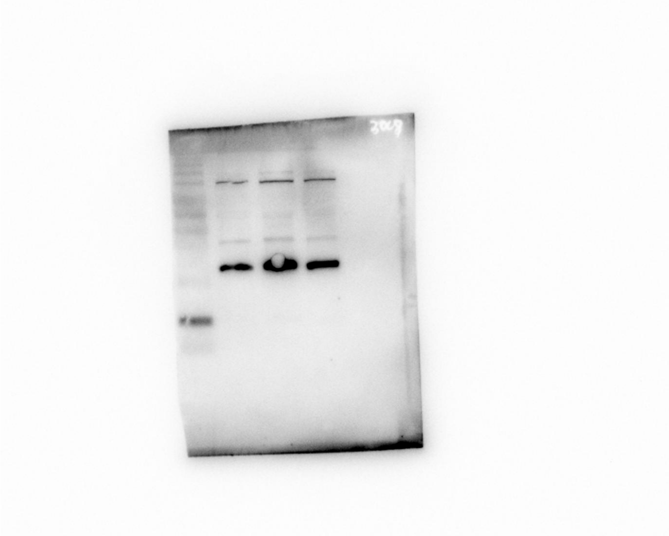

R2 Full unedited gel for fig .5A   ob mice p-RIPK1

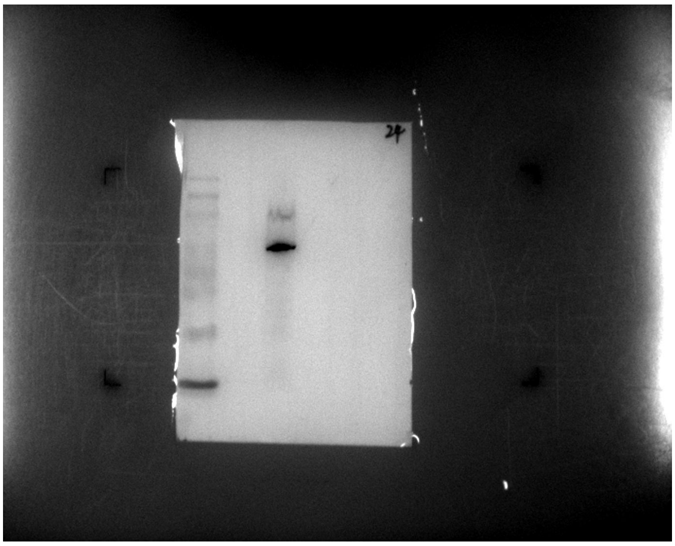

R2 Full unedited gel for fig .5A   ob mice p-RIPK3

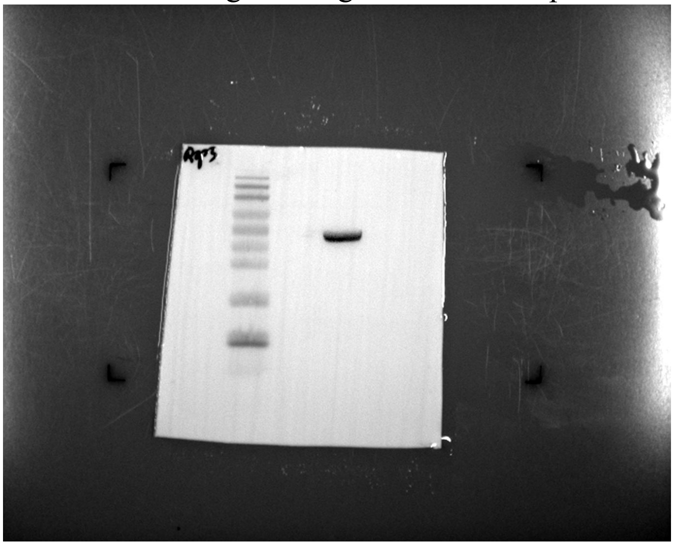

R2 Full unedited gel for fig .5A   ob mice RIPK3

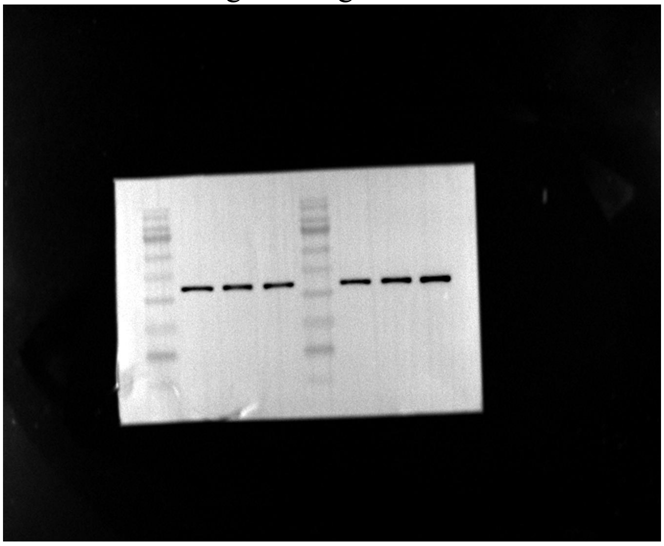

R2+R3 Full unedited gel for fig .5A   ob mcie GAPDH

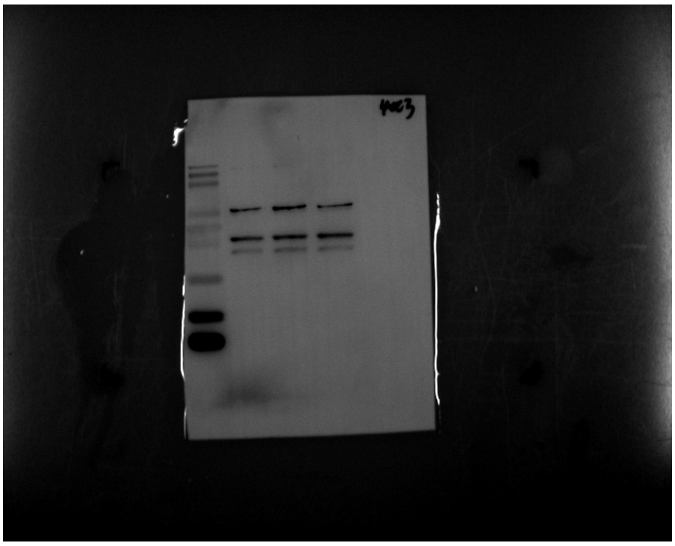

R3 Full unedited gel for fig .5A   ob mcie RIPK1

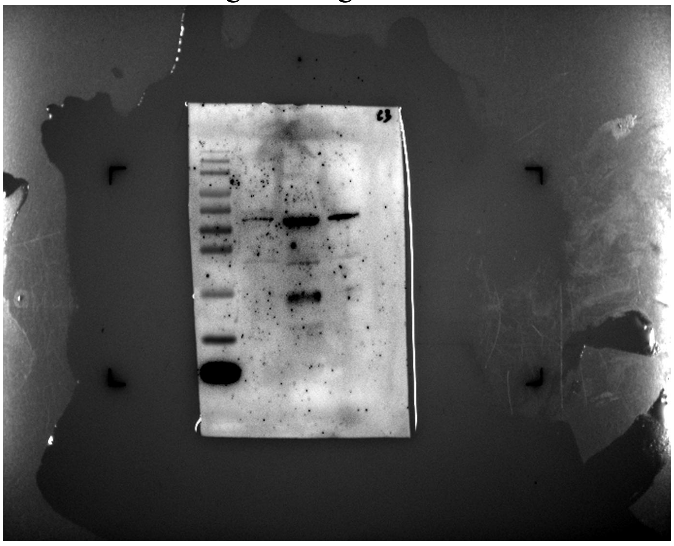

R3 Full unedited gel for fig .5A   ob mice Caspase-3(10s)

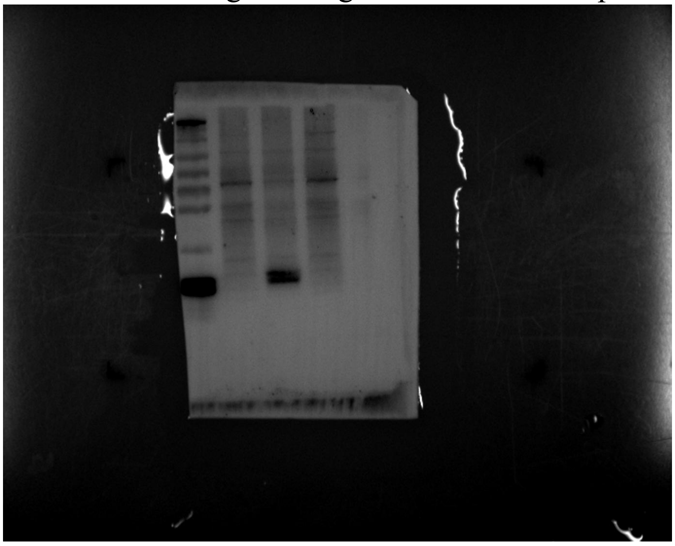

R3 Full unedited gel for fig .5A   ob mice Caspase-8

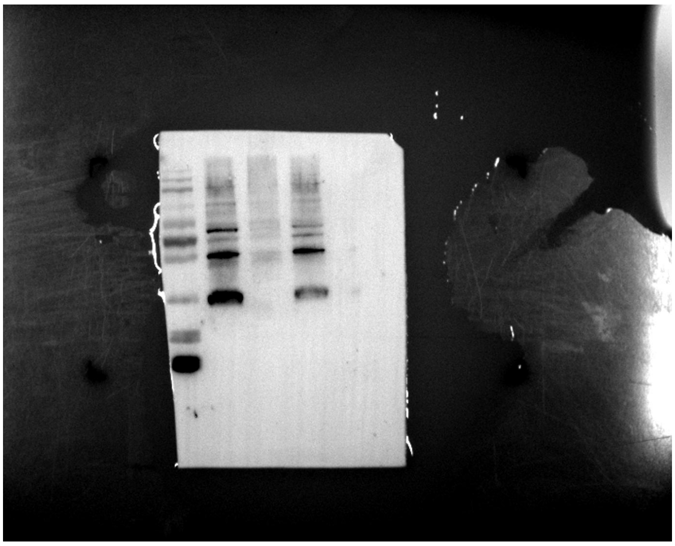

R3 Full unedited gel for fig .5A   ob mice p-AMPK

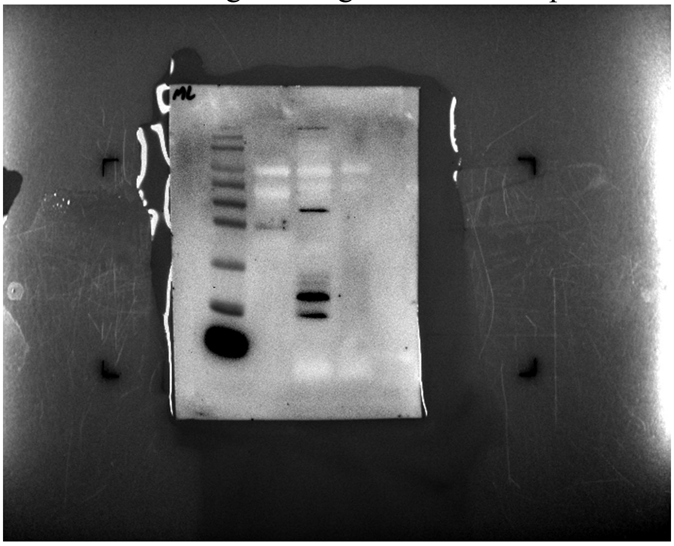

R3 Full unedited gel for fig .5A   ob mice p-MLKL

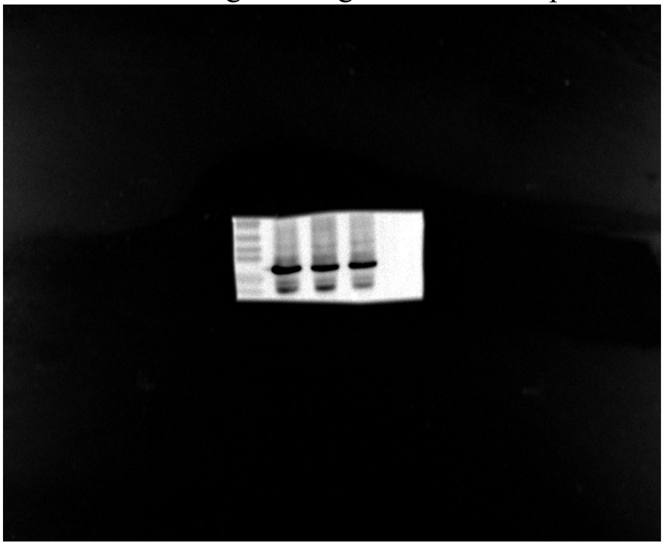

R3 Full unedited gel for fig .5A   ob mice p-RIPK1

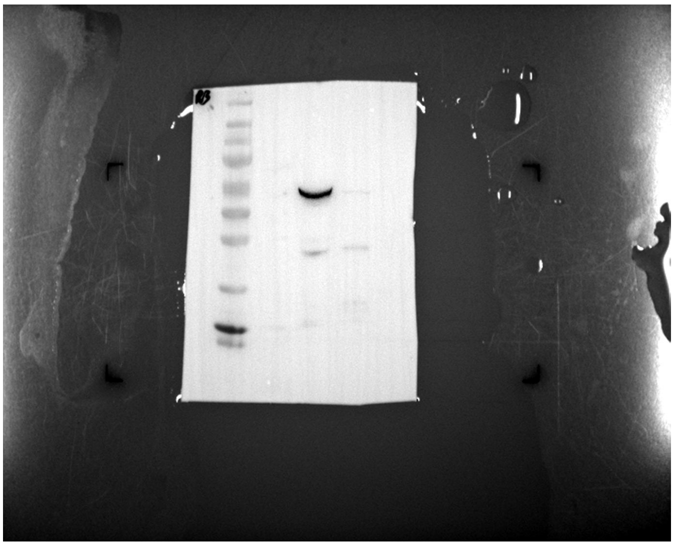

R3 Full unedited gel for fig .5A   ob mice p-RIPK3

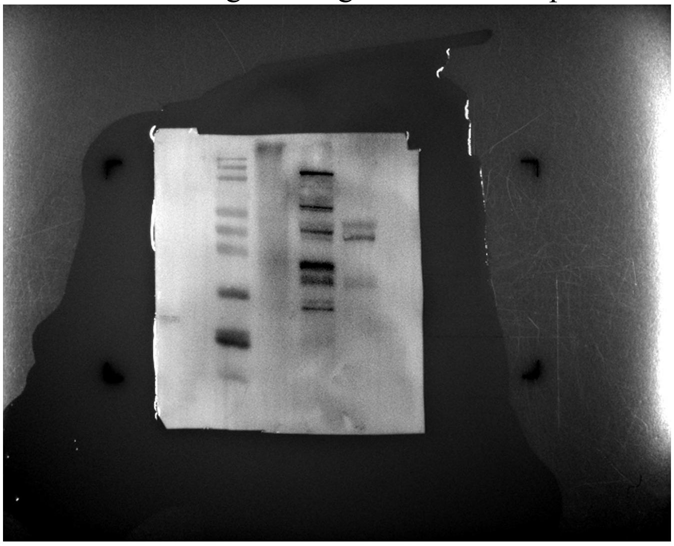

R3 Full unedited gel for fig .5A   ob mice RIPK3

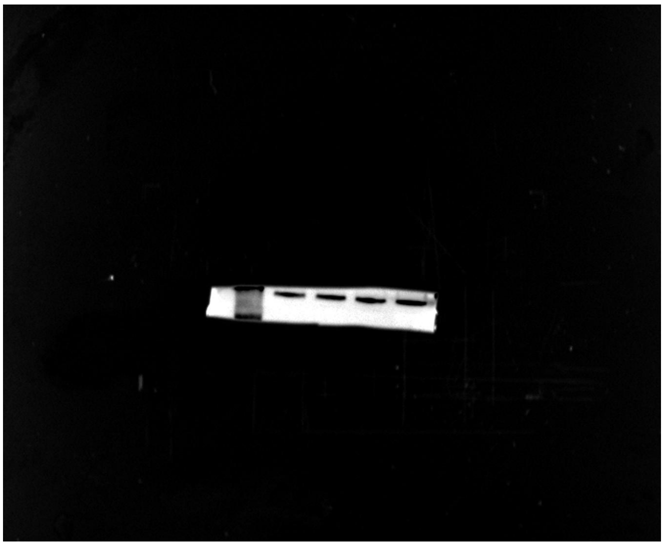

R2 Full unedited gel for fig .6A sicaspase-8： GAPDH

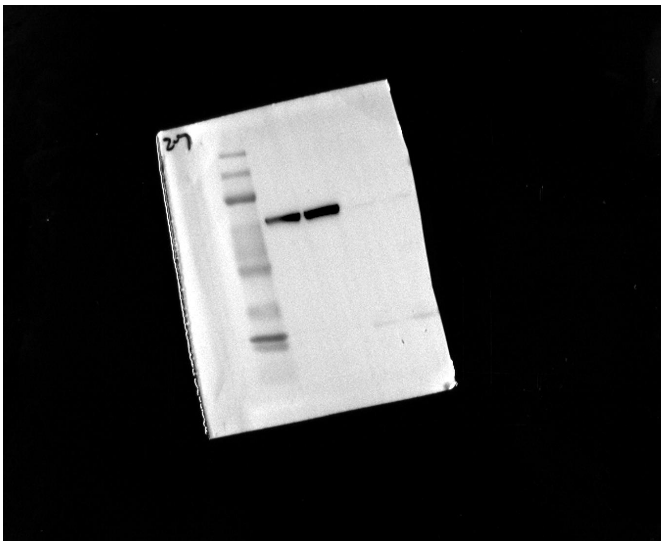

R2 Full unedited gel for fig .6A sicaspase-8： pro-Caspase8

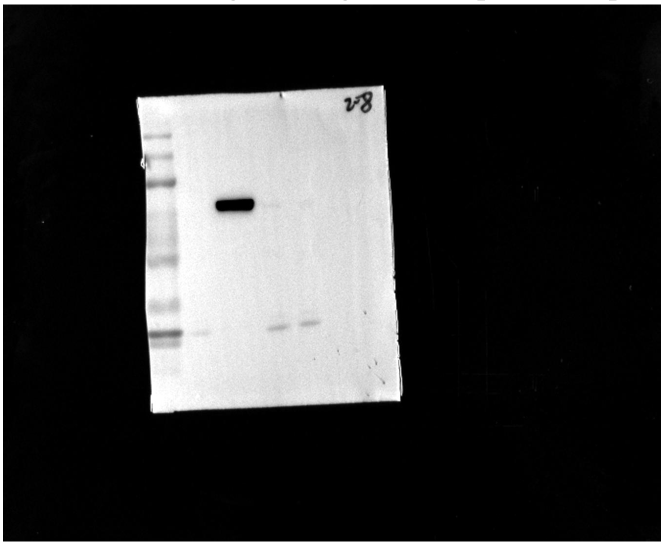

R2 Full unedited gel for fig .6H sicaspase-8 Caspase-8

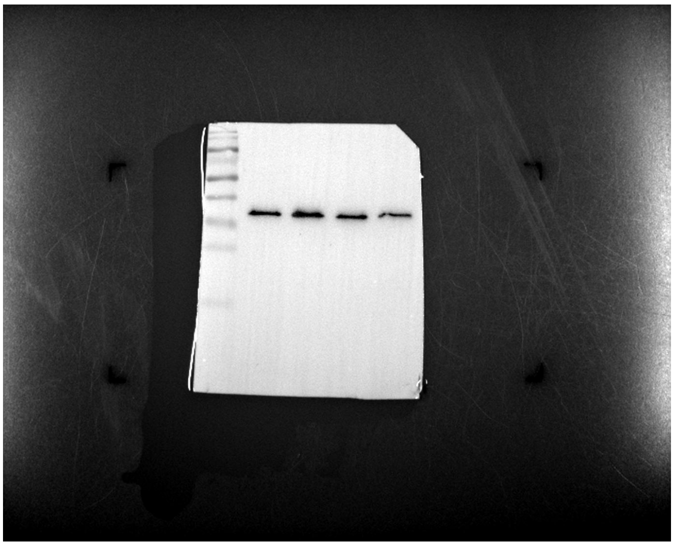

R2 Full unedited gel for fig .6H sicaspase-8 GAPDH

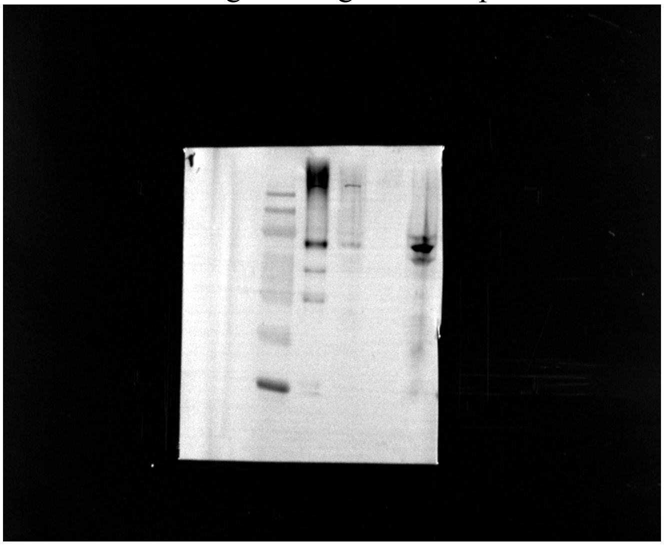

R2 Full unedited gel for fig .6H sicaspase-8 p-AMPK

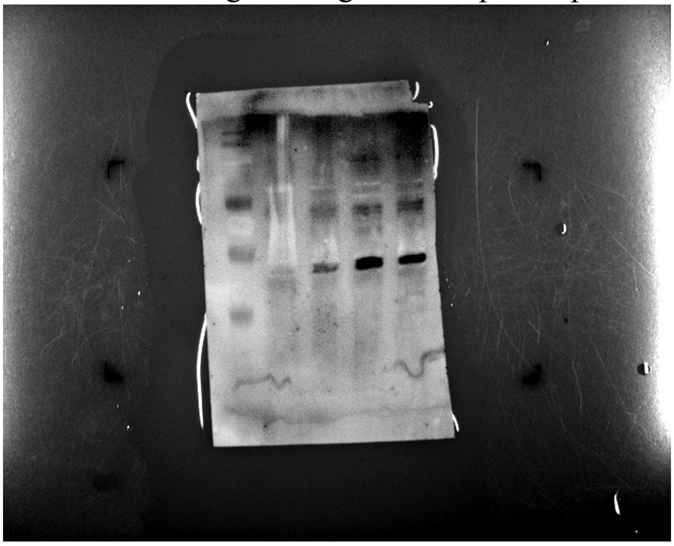

R2 Full unedited gel for fig .6H sicaspase-8 p-MLKL

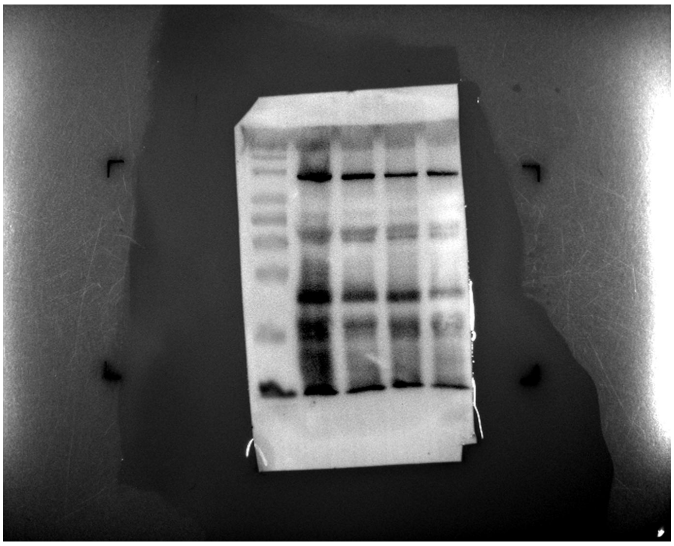

R2 Full unedited gel for fig .6H sicaspase-8 p-RIPK1

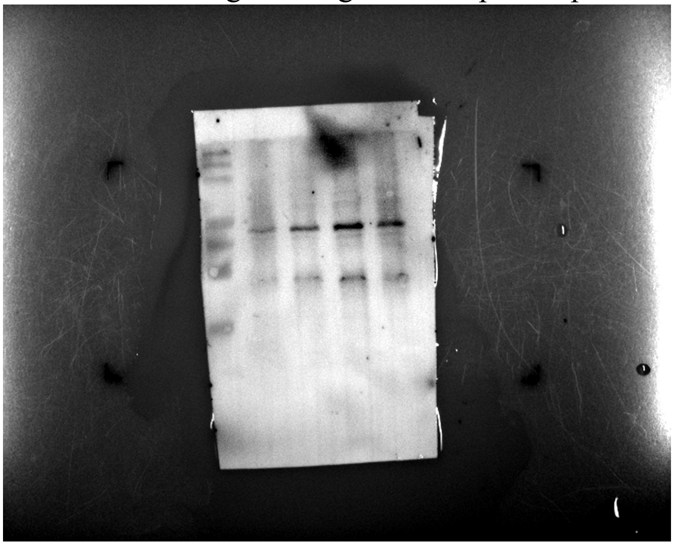

R2 Full unedited gel for fig .6H sicaspase-8 p-RIPK3

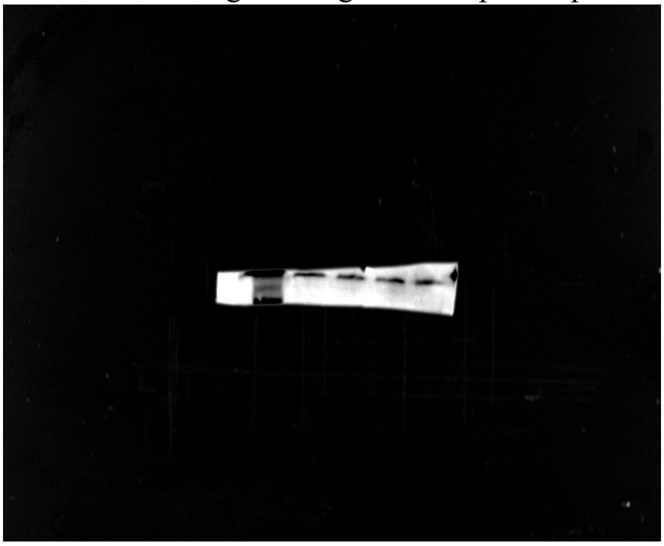

R3 Full unedited gel for fig .6A sicaspase-8： GAPDH

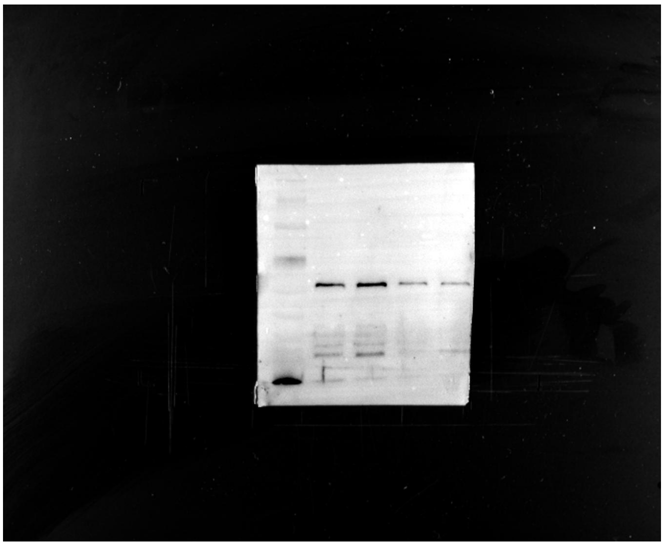

R3 Full unedited gel for fig .6A sicaspase-8： pro-Caspase8

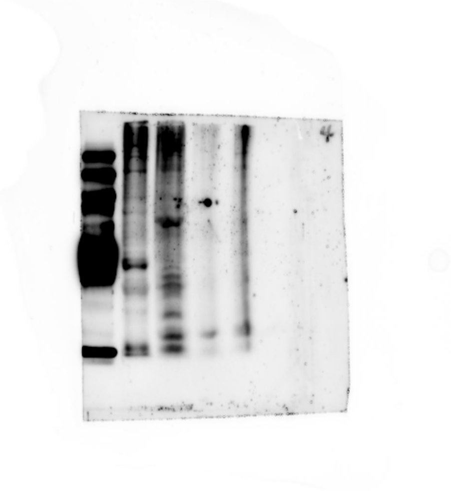

R3 Full unedited gel for fig .6H sicaspase-8 Caspase-8

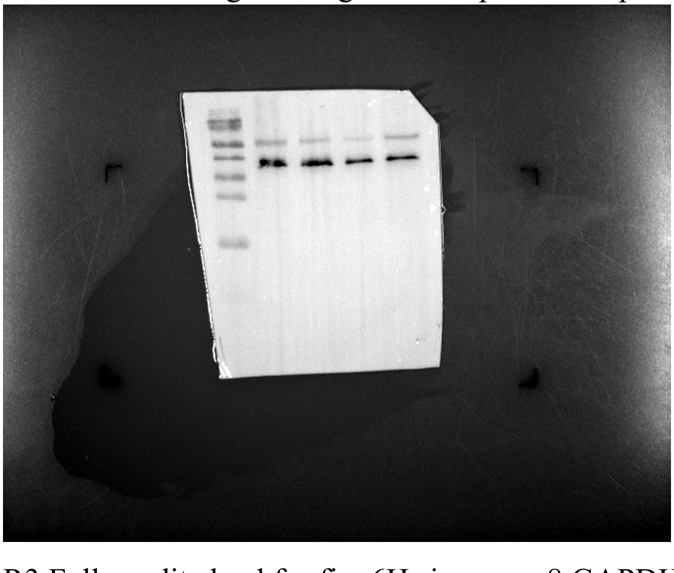

R3 Full unedited gel for fig .6H sicaspase-8 GAPDH

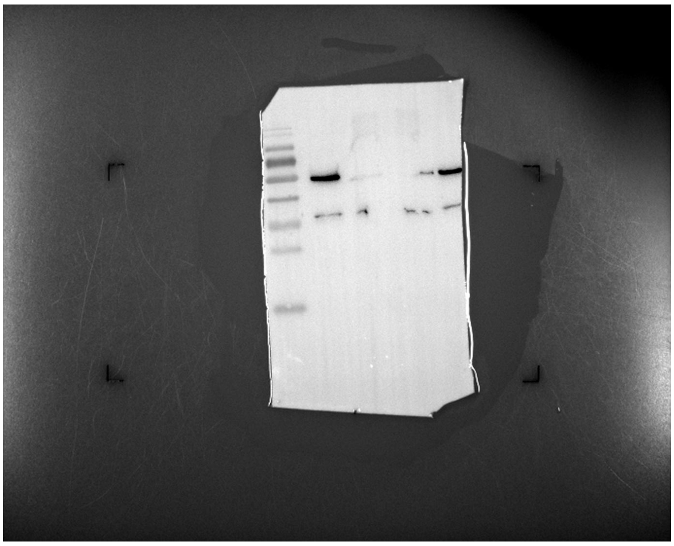

R3 Full unedited gel for fig .6H sicaspase-8 p-AMPK

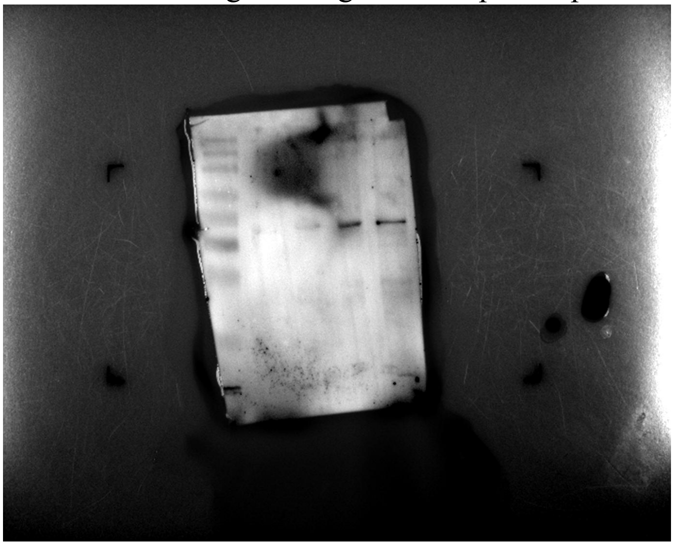

R3 Full unedited gel for fig .6H sicaspase-8 p-MLKL

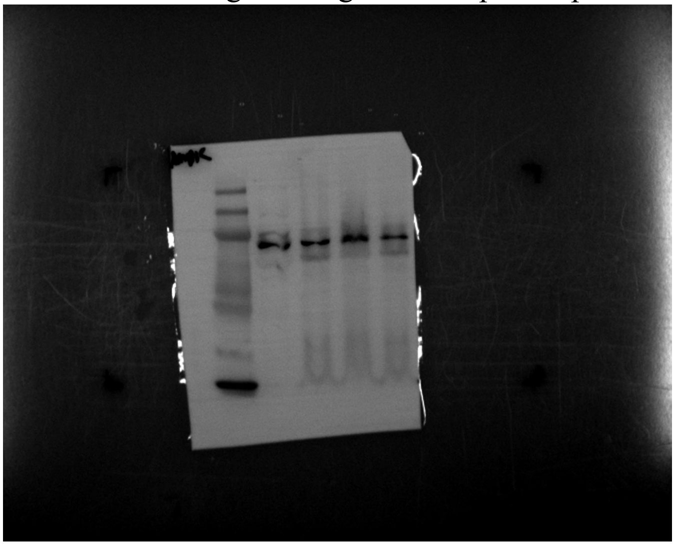

R3 Full unedited gel for fig .6H sicaspase-8 p-RIPK1

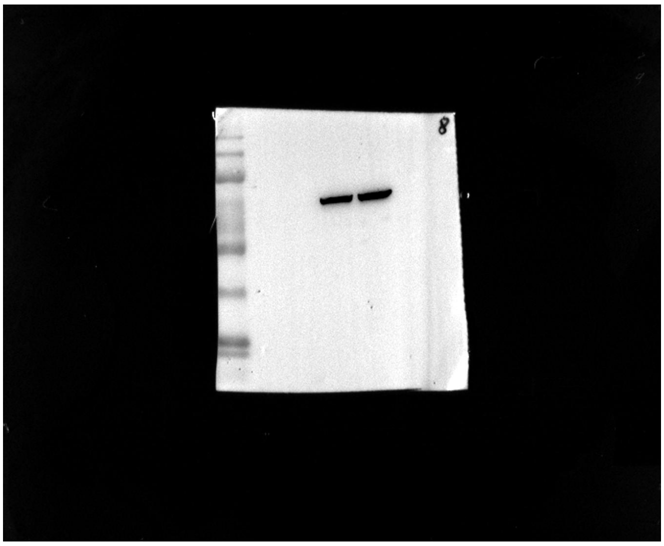

R3 Full unedited gel for fig .6H sicaspase-8 p-RIPK3

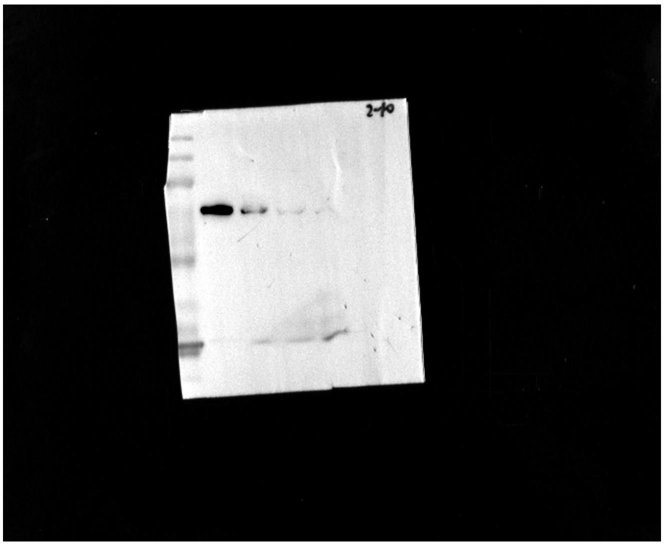

R2 Full unedited gel for fig.7C CHX CER caspase-8

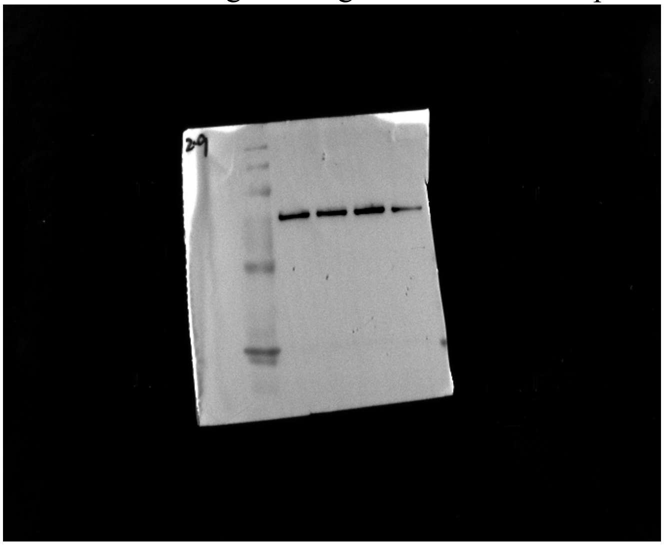

R2 Full unedited gel for fig.7C CHX CER+AICAR caspase-8

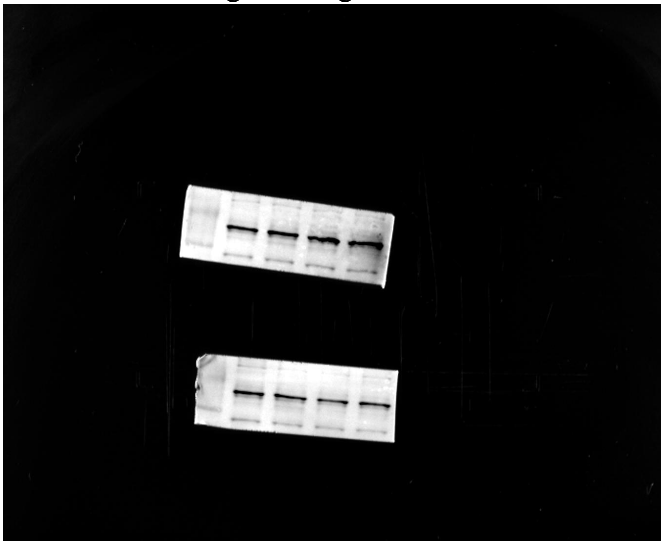

R2+R3 Full unedited gel for fig.7C CHX CER GAPDH

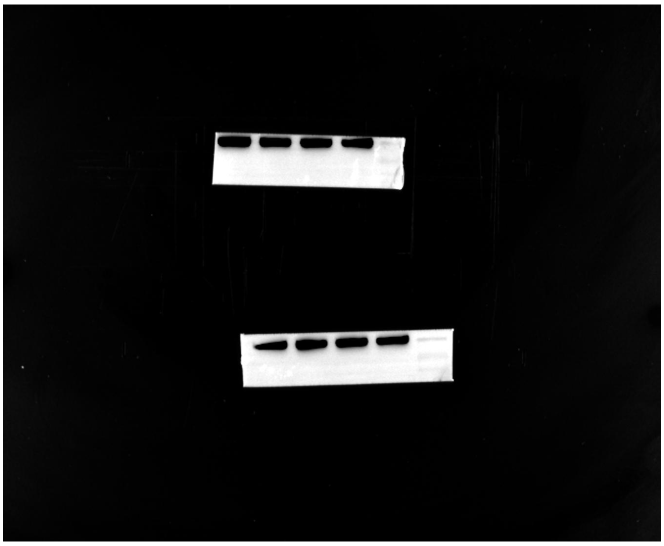

R2+R3 Full unedited gel for fig.7C CHX CER+AICAR GAPDH

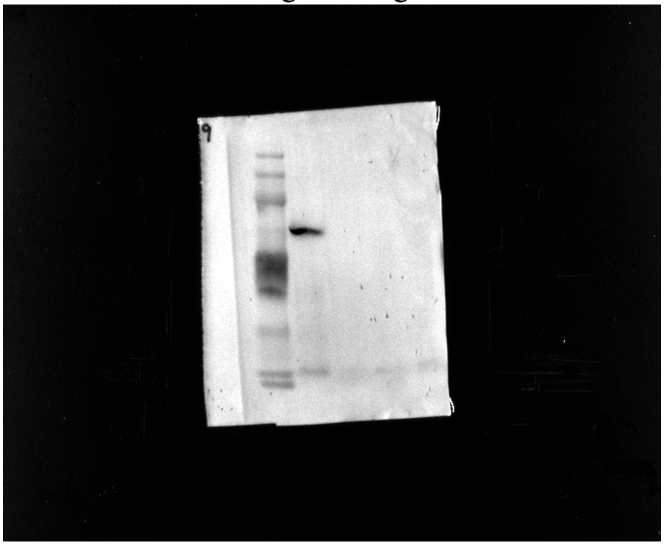

R3 Full unedited gel for fig.7C CHX CER caspase-8

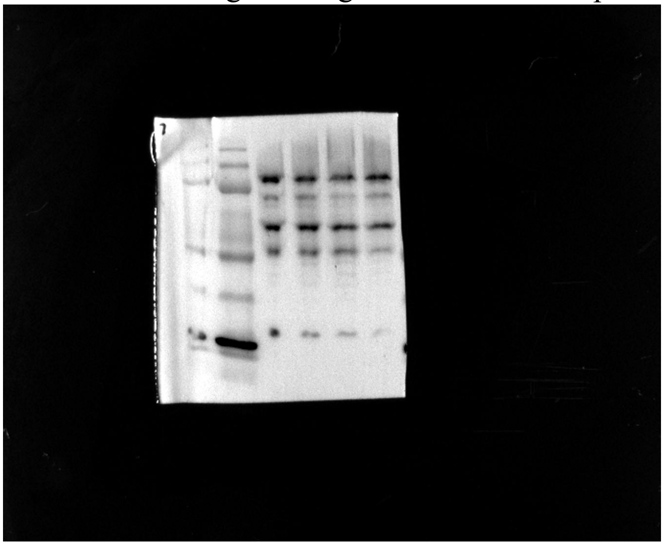

R3 Full unedited gel for fig.7C CHX CER+AICAR caspase-8

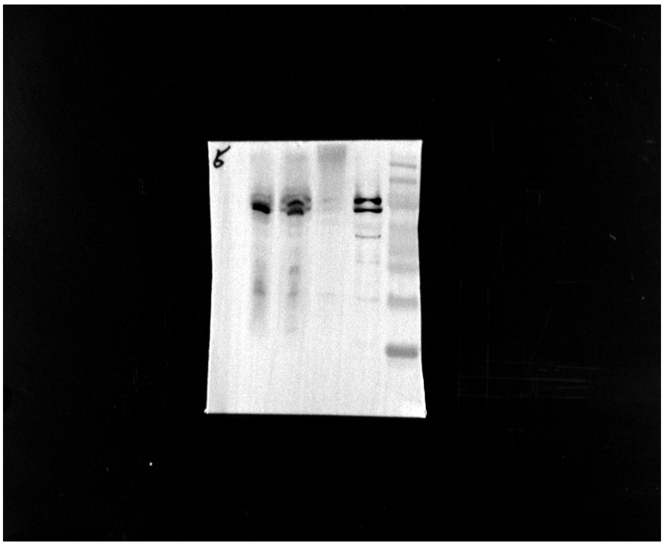

R2 Full unedited gel for fig.7E MG132 caspase-8

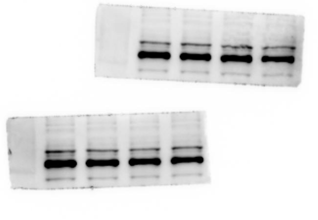

R2+R3 Full unedited gel for fig.7E MG132 GAPDH

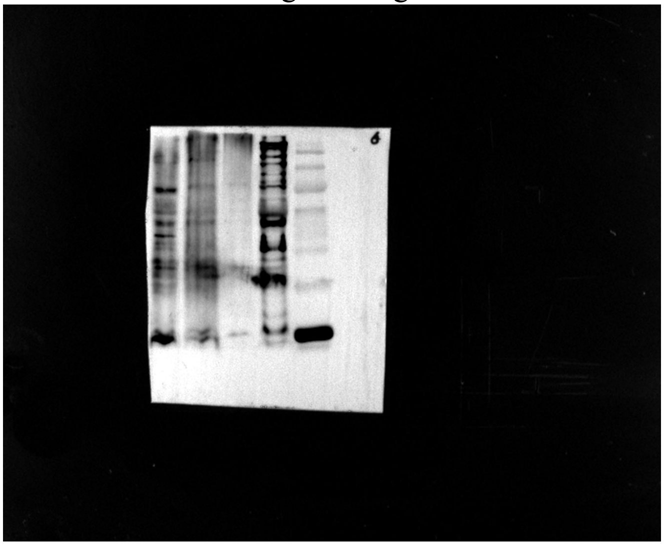

R3 Full unedited gel for fig.7E MG132 caspase-8

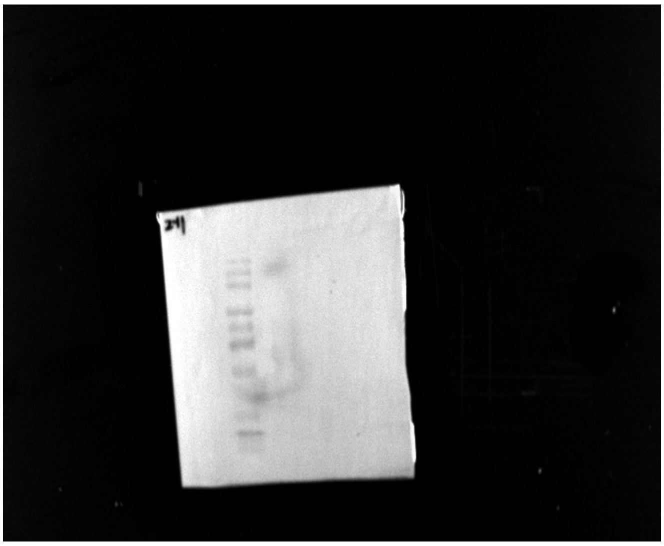

R2 Full unedited gel for fig.7B IgG caspase-8

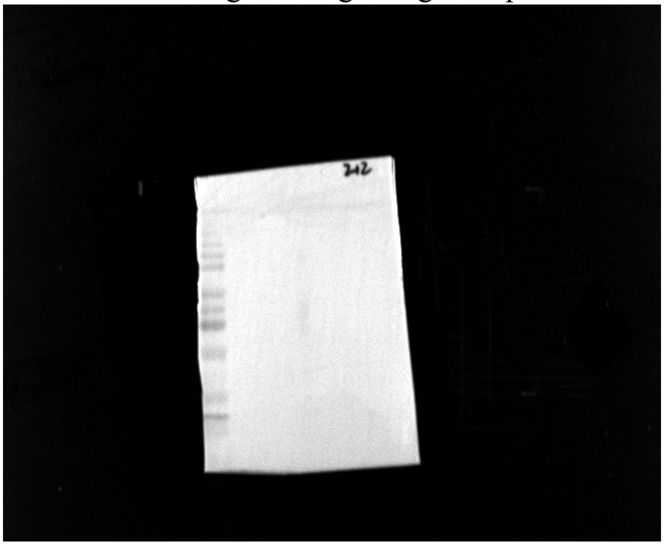

R2 Full unedited gel for fig.7B IgG p-AMPK

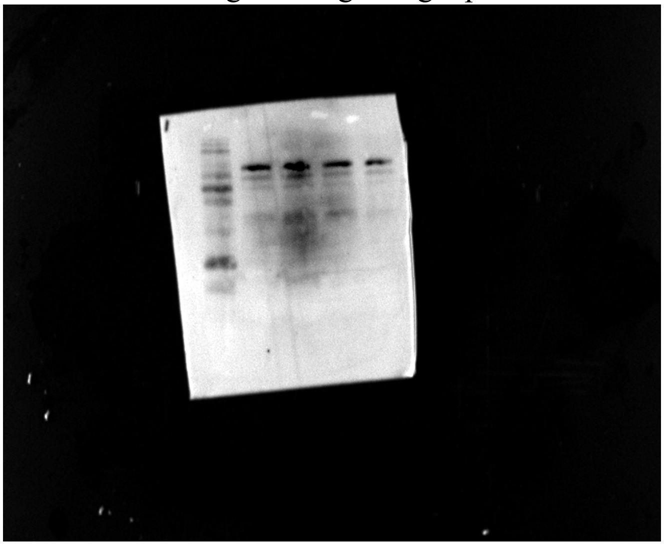

R2 Full unedited gel for fig.7B Input caspae-8

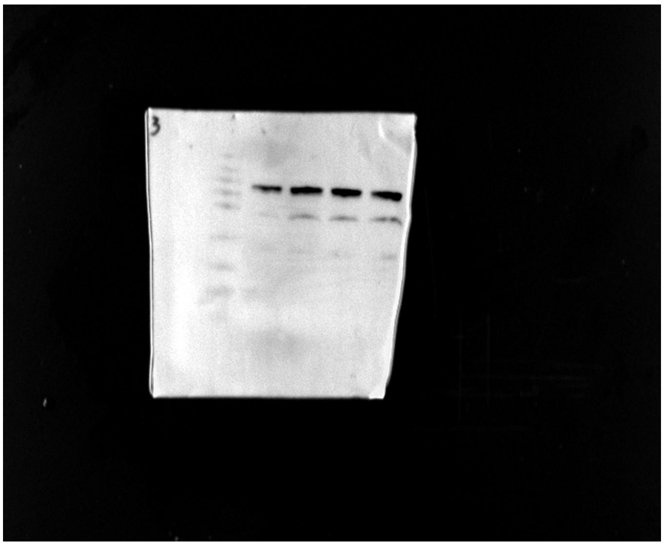
R2 Full unedited gel for fig.7B Input p-AMPK
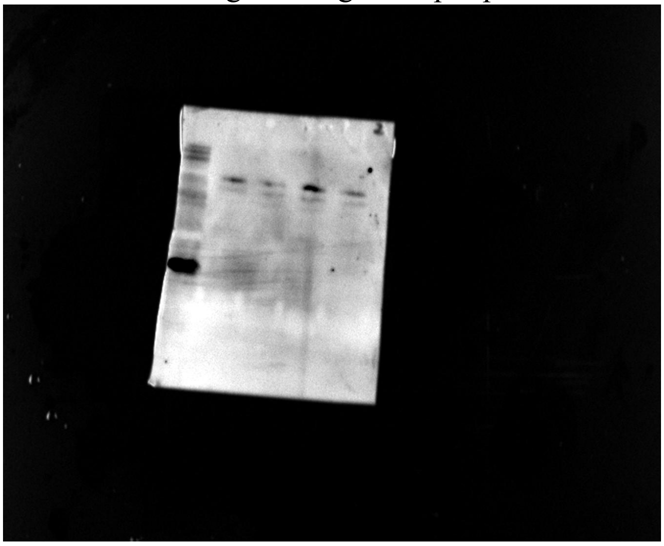

R2 Full unedited gel for fig.7B IP caspase-8
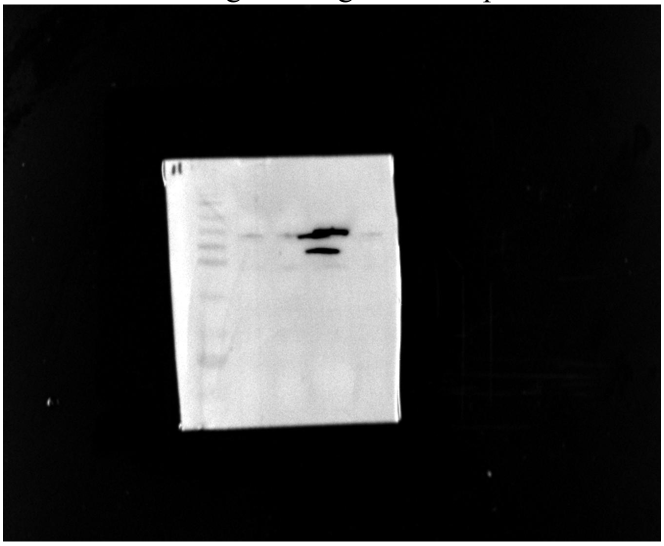

R2 Full unedited gel for fig.7B IP p-AMPK

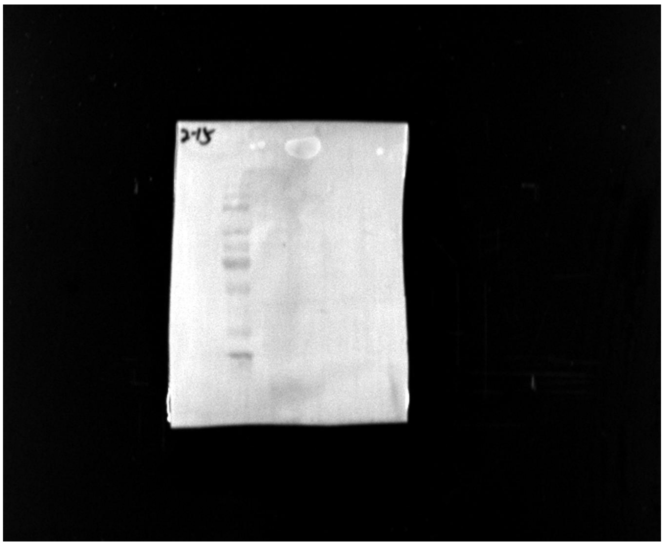

R3 Full unedited gel for fig.7B IgG caspase-8

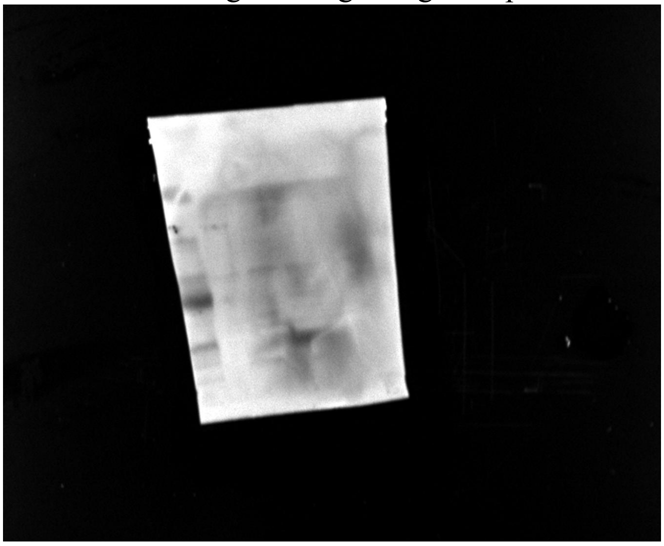

R3 Full unedited gel for fig.7B IgG p-AMPK

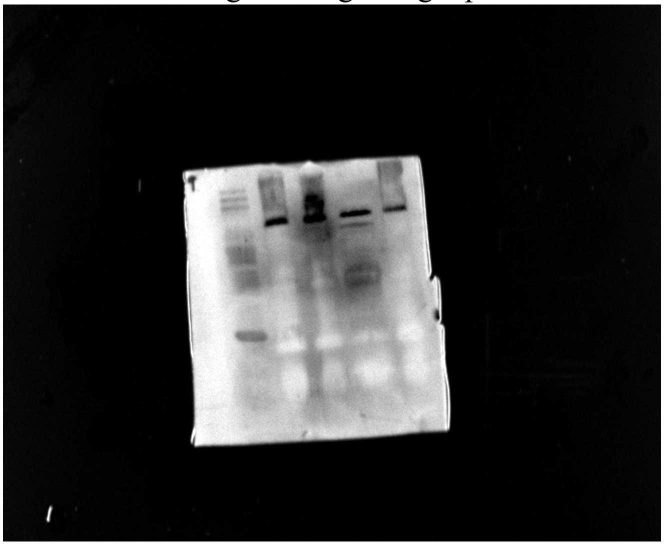

R3 Full unedited gel for fig.7B Input caspae-8

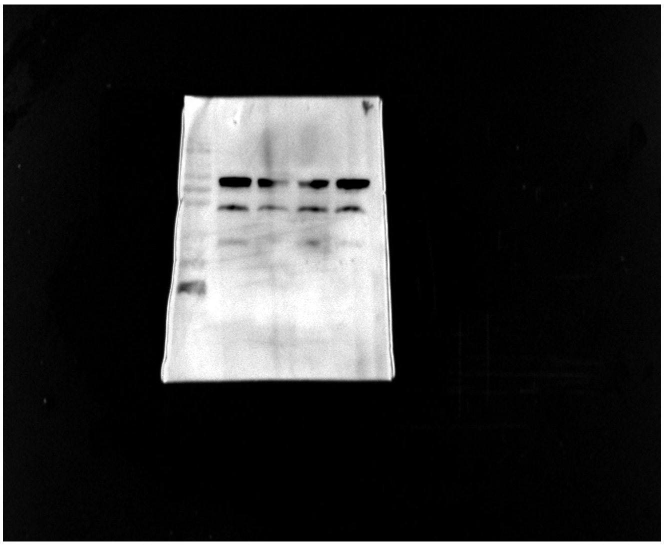

R3 Full unedited gel for fig.7B Input p-AMPK

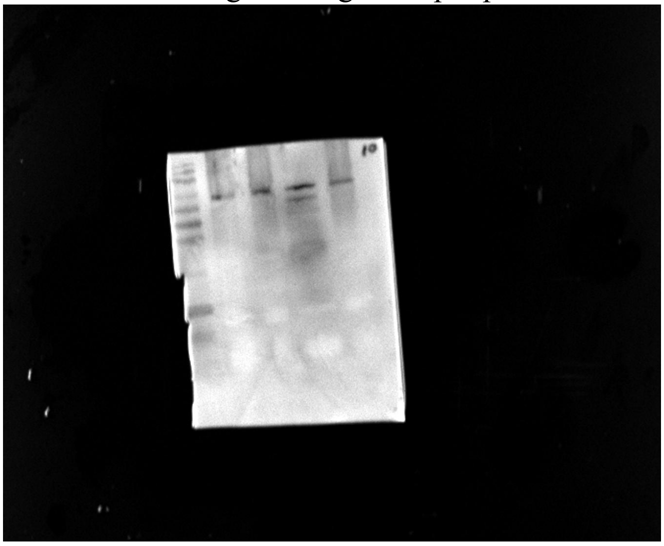

R3 Full unedited gel for fig.7B IP caspase-8

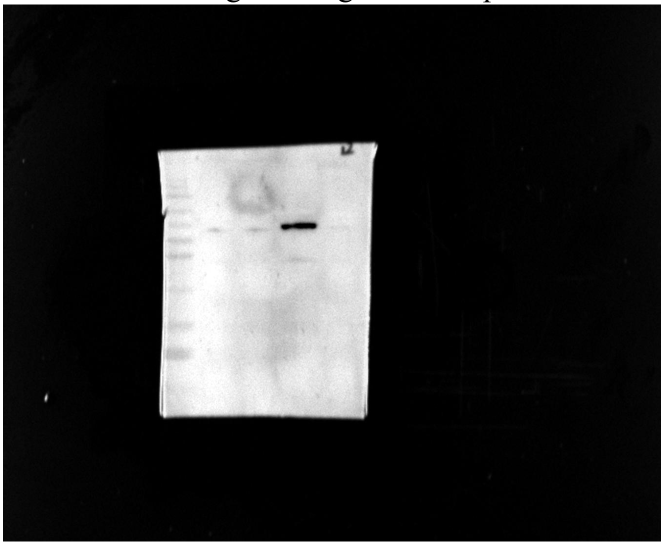

R3 Full unedited gel for fig.7B IP p-AMPK
