# Supplementary material for: Activation of AMPK ameliorates acute severe pancreatitis by suppressing pancreatic acinar cell necroptosis in obese mice models
Source: Cell Death Discov. 2023 Sep 30;9:363. doi: 10.1038/s41420-023-01655-z (PMC10542799; doi:10.1038/s41420-023-01655-z)
Supplement: Supplementary file 2 — Supplemental Materials and legends [file 41420_2023_1655_MOESM2_ESM.docx]

**Supporting Information for:**

Activation of AMPK Ameliorates Acute Severe Pancreatitis by Suppressing Pancreatic Acinar Cell Necroptosis in Obese Mice Models.

Kunlei Wang1,2,3, Anbang Zhao1,2,3, Dilinigeer Tayier1,2,3, Kai Tan1,2,3, Wenjing Song1,2,3, Qian Cheng1,2,3, Xinyin Li1,2,3, Zhinan Chen1,2,3, Qifeng Wei1,2,3, Yufeng Yuan1,3*, Zhiyong Yang1,2,3*

1Department of Hepatobiliary and Pancreatic Surgery, Zhongnan Hospital of Wuhan University, Wuhan, China.

2Pancreatic Surgery Center, Zhongnan Hospital of Wuhan University, Wuhan, China.

3Clinical Medicine Research Center for Minimally Invasive Procedure of Hepatobiliary & Pancreatic Diseases of Hubei Province, Hubei, China.

*Corresponding Authors:

Yufeng Yuan: yuanyf1971@whu.edu.cn

Zhiyong Yang: [yangzhiyong@whu.edu.cn](mailto:yangzhiyong@whu.edu.cn)

**This PDF file includes:**

Supporting Materials and Methods

Figures S1 to S5

**Supporting Materials and Methods**

**Cell culture**

3T3-L1 (CL-0006, Procell, China) were kindly provided by Procell Life Sciences & Technology Co. Ltd and cultured in DMEM medium (Procell) supplemented with 10% fetal bovine serum (10099-141, Gibco, USA). 3T3-L1 cells were plated into 6 well plates or 96 well plates and cultured for 48h after the confluence reached about 100%. 3T3-L1 pre-adipocytes were induced to differentiate by treated with ADP I (PD-004, Procell, China) for 48h and treated with ADP II for 48h, then were further maintained in the DMEM medium supplemented with 10% fetal bovine serum for 10 to 12 days.

AR42J cells were treated with 1μmol/L CER for 6h. Then, culture supernatants were obtained for further research. Differentiated 3T3-L1 cells were treated with 1μmol/L CER or added 100μL culture supernatants from AR42J cells which were treated with 1μmol/L CER (CER-S). Then, culture supernatants and cells were obtained at 24h after CER or culture supernatants treatment for further analyses. At 1h prior to CER or culture supernatants from AR42J cells treatment, the culture medium of differentiated 3T3-L1 cells was supplemented with 1mmol/L AICAR (CER+AICAR group and CER-S+AICAR group) or 10μmol/L CC (CER+CC group and CER-S+CC group).

**Oil Red O staining**

Differentiated 3T3-L1 cells were washed three times using 1× PBS and fixed in 4% buffered formaldehyde for 30min. Cells were stained in the Oil Red O (PD-004, Procell, China)) working solution for 30min and washed three times with water. Images were acquired by fluorescence microscopy (Olympus, Tokyo, Japan).

**Cell viability**

Cell viability was detected by using the CCK-8 assay (CA1210, Solarbio, China). Differentiated 3T3-L1 cells were cultured until the confluence reached about 100% and treat with 1μmol/L CER or added 100μL culture supernatants from AR42J cells which were treated with 1μmol/L CER for 6h. After 20h, differentiated 3T3-L1 cells were added wih 10μL CCK-8 reagent for another 4h, and then, the absorbance at 450nm was measured with a microplate reader.

#### **Biochemical Measurements**

supernatant of differentiated 3T3-L1 cell was measured using glycerol kits (Applygen, Beijing, China).

**Statistical Analysis**

Data are expressed as means±SEM and were analyzed using Graphpad prism 8 (GraphPad Software Inc., La Jolla, CA, USA). Between group comparisons of means was performed using the Student's t-test. Significant levels were set as: ns: p > 0.0.5; *: p < 0.05; **: p < 0.01; ***: p < 0.001.


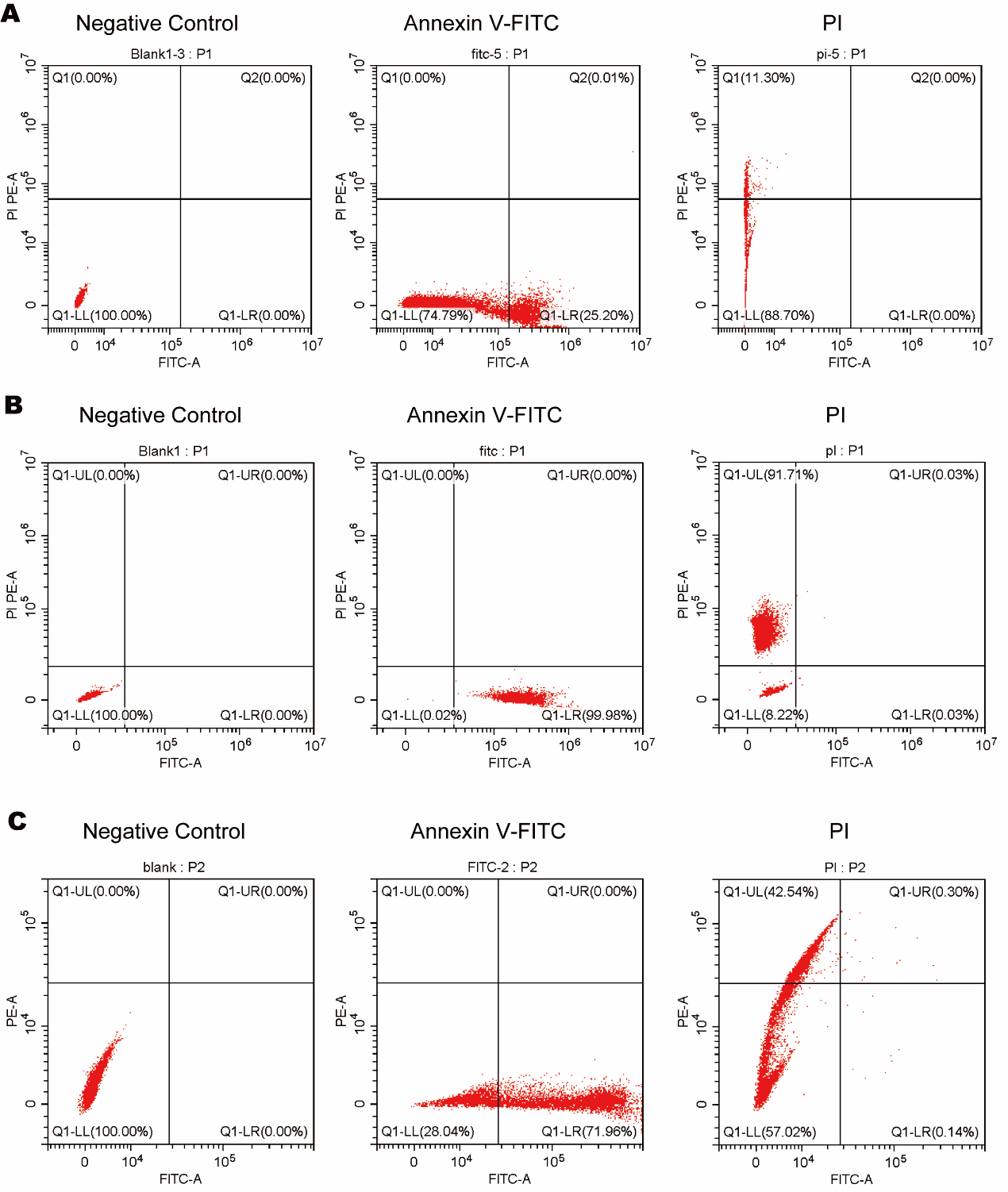


**Fig S1 Negative control gating.** **A** Negative control gating for Fig. 4D. **B** Negative control gating for Fig. 6C. **C** Negative control gating for Fig. S4B and Fig. S5A.


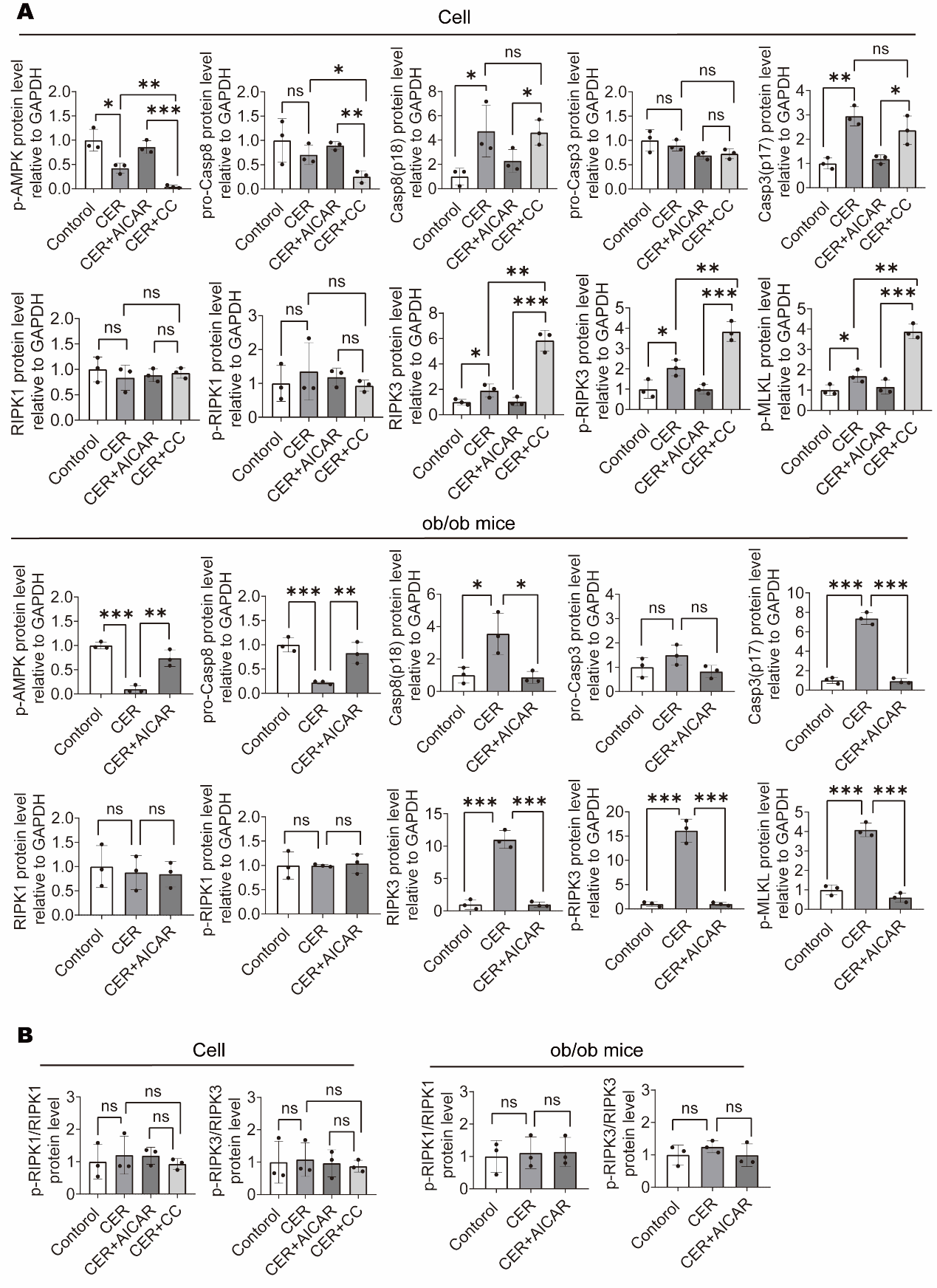


**Fig S2 Quantitative analysis of WB.** **A** Quantification of WB for Fig. 5A. **B** Quantitative analysis of phospo/ total protein for Fig. 5A.


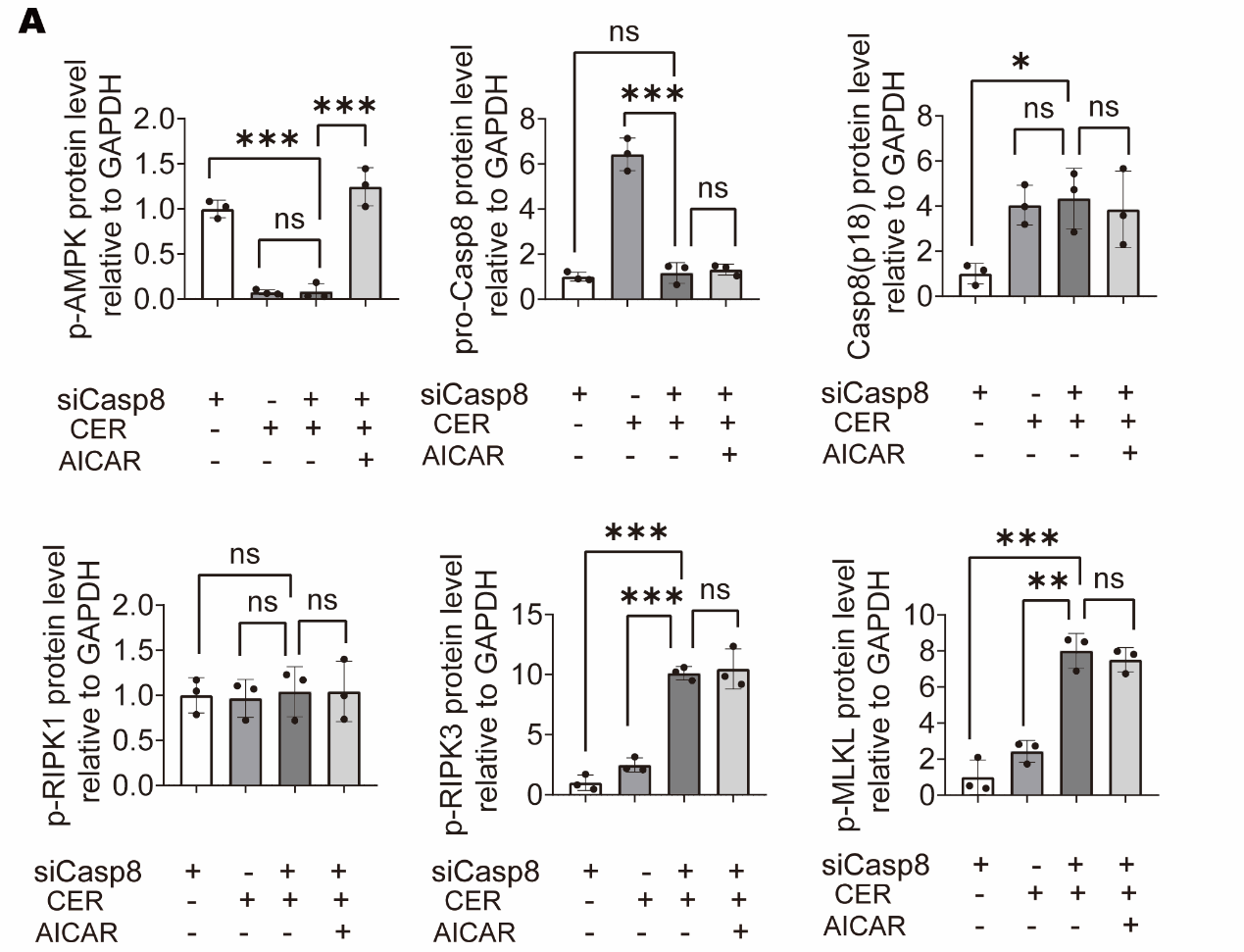


**Fig S3 Quantitative analysis of WB.** **A** Quantification of WB for Fig. 6H.


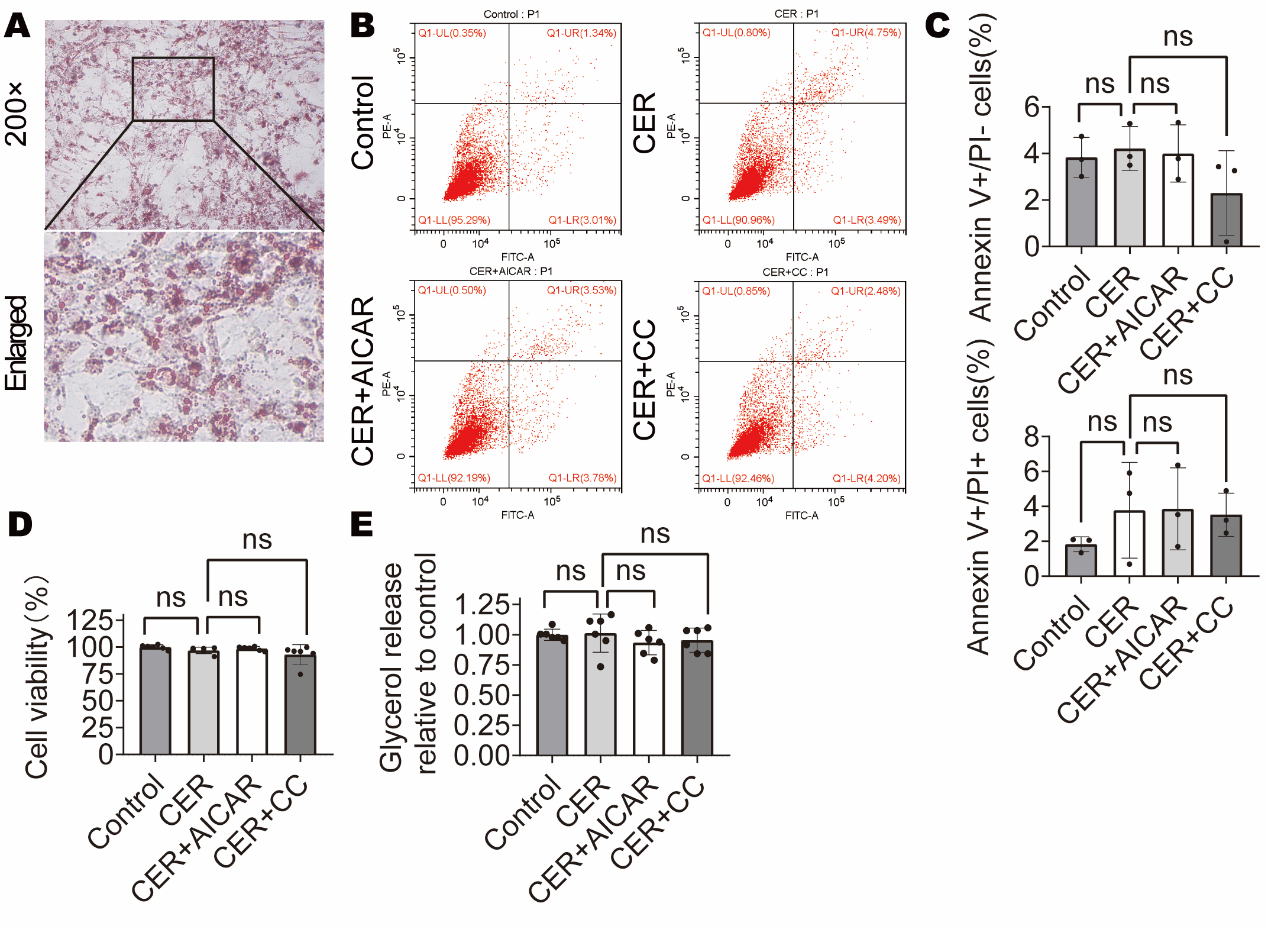


**Fig S4 Caerulein has no direct damage to adipocytes.** Differentiated 3T3-L1 cells were treated with 1μmol/L CER for 24h. **A** Oil Red O staining for differentiated 3T3-L1 cells (200× and 600×) **B, C** Flow cytometry and quantification of apoptotic or necrotic cells (n=3) **D** Cell viability for differentiated 3T3-L1 cells (n=6). **E** Glycerol in supernatants of differentiated 3T3-L1 cells (n=6).


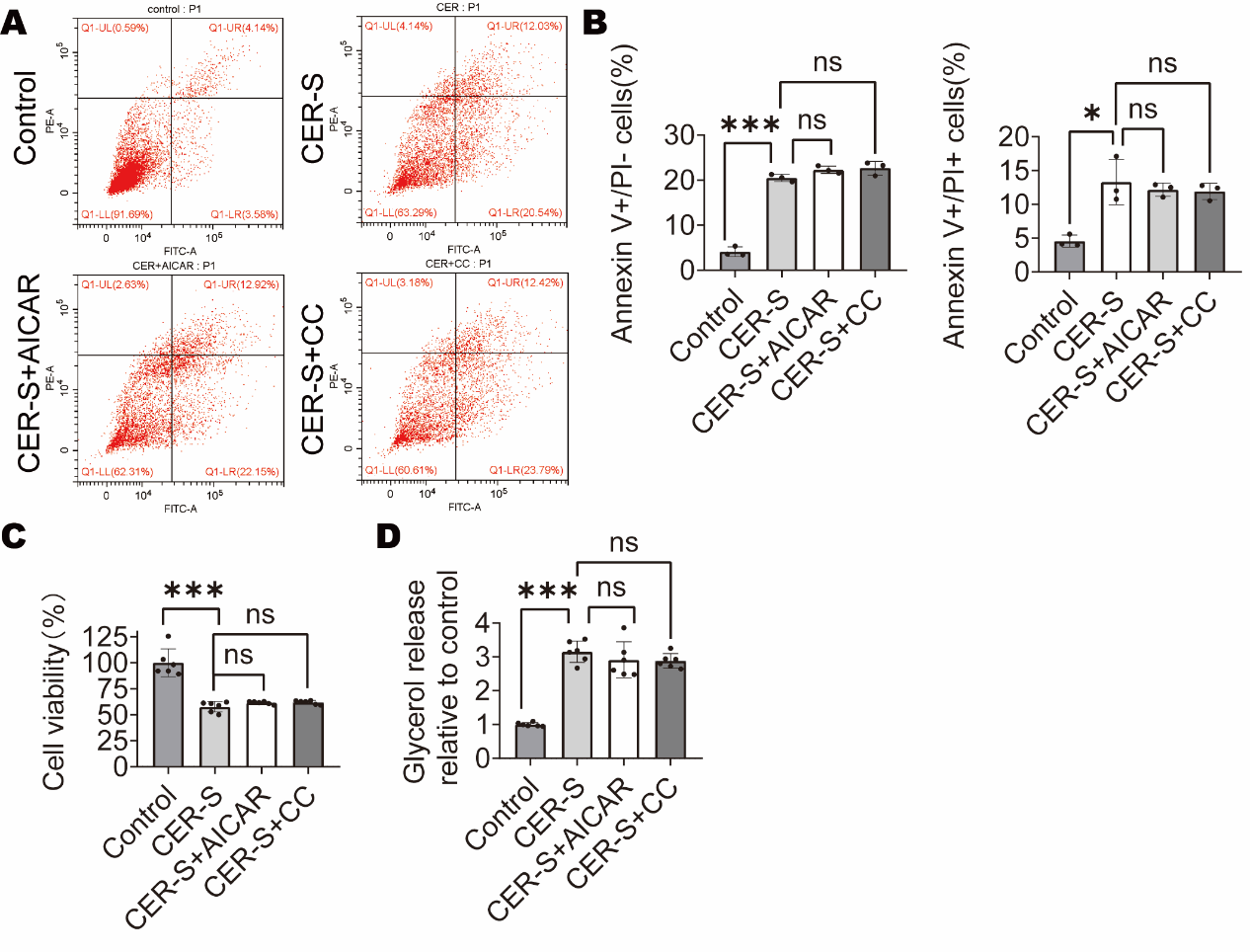


**Fig S5 AICAR/CC has no protective effects/direct damage to adipocytes.** Differentiated 3T3-L1 cells were treated with culture supernatants from AR42J cells (CER-S) for 24h. **A, B** Flow cytometry and quantification of apoptotic or necrotic cells (n=3) **C** Cell viability for differentiated 3T3-L1 cells (n=6). **D** Glycerol in supernatants of AR42J cells (n=6).
